# Role of *Rubus chingii* BBX gene family in anthocyanin accumulation during fruit ripening

**DOI:** 10.3389/fpls.2024.1427359

**Published:** 2024-08-02

**Authors:** Zhangting Xu, Guihua Zhang, Junyu Chen, Yuxin Ying, Lingtiao Yao, Xiaoxian Li, Jaime A. Teixeira da Silva, Zhenming Yu

**Affiliations:** ^1^ School of Pharmaceutical Sciences, Academy of Chinese Medical Sciences, Zhejiang Chinese Medical University, Hangzhou, China; ^2^ Zhejiang Academy of Forestry, Hangzhou, China; ^3^ College of Food and Health, Zhejiang A & F University, Hangzhou, China; ^4^ Independent Researcher, Miki, Kagawa, Japan; ^5^ Songyang Institute of Zhejiang Chinese Medical University, Lishui, China

**Keywords:** *Rubus chingii*, BBX, anthocyanidin, expression analysis, fruit ripening

## Abstract

The B-box (BBX) family, which is a class of zinc finger transcription factors, exhibits special roles in plant growth and development as well as in plants’ ability to cope with various stresses. Even though *Rubus chingii* is an important traditional medicinally edible plant in east Asia, there are no comprehensive studies of BBX members in *R. chingii*. In this study, 32 RcBBX members were identified, and these were divided into five groups. A collinearity analysis showed that gene duplication events were common, and when combined with a motif analysis of the *RcBBX* genes, it was concluded that group V genes might have undergone deletion of gene fragments or mutations. Analysis of *cis*-acting elements revealed that each *RcBBX* gene contained hormone-, light-, and stress-related elements. Expression patterns of the 32 *RcBBX* genes during fruit ripening revealed that highest expression occurred at the small green fruit stage. Of note, the expression of several *RcBBX* genes increased rapidly as fruit developed. These findings, combined with the expression profiles of anthocyanin biosynthetic genes during fruit ripening, allowed us to identify the nuclear-targeted RcBBX26, which positively promoted anthocyanin production in *R. chingii*. The collective findings of this study shed light on the function of *RcBBX* genes in different tissues, developmental stages, and in response to two abiotic stresses.

## 1 Introduction

Transcription factors (TFs) are a class of proteins that are able to bind to specific genes, allowing for gene expression to be regulated (Spitz and Furlong, 2012). They can act as communication hubs by networking with other interacting proteins, synergizing with them, and resulting in gene transcription (Stortz et al., 2024). Among a wealth of plant TFs, the zinc finger protein is a specialized TF that folds upon itself to form a finger structure, and is composed of Zn (II) and different amounts of histidine (His) and cysteine (Cys). Different zinc finger proteins play varying roles in plants (Laity et al., 2001). For instance, zinc finger proteins are involved in mRNA recognition, DNA packing, transcription activation, and protein-protein interactions, and can thus impact plant growth and development (Noman et al., 2019), while playing an integral role in plants’ responses to stresses (Cao et al., 2023).

The B-box domain protein, or BBX, which is one type of zinc finger protein, has attracted widespread attention. In recent years, BBX families have been identified and functionally characterized in the genomes of several plants, such as *Dioscorea opposita* (Chang et al., 2023), *Vaccinium corymbosum* (Liu et al., 2023), *Dendrobium officinale* (Cao et al., 2019), *Glycine max* (Shan et al., 2022), and *Vitis vinifera* (Wei et al., 2020). The BBX family is well-studied in the model plant *Arabidopsis thaliana* at physiological, molecular, and biochemical levels (Khanna et al., 2009). All BBX proteins contain one or two highly conserved B-box domains, B-box1 (C-X_2_-C-X_7-8_-C-X_2_-D-X-A-X-L-C-X_2_-C-D-X_3_-HB) and B-box2 (C-X_2_-C-X_3_-P-X_4_-C-X_2_-D-X_3_-L-C-X_2_-C-D-X_3_-H), with about 40 amino acids at the N-terminus, and some of them may possess a conserved CCT (CONSTANS, CO-like and TOC1) domain with 42-43 amino acids at the C-terminus (Cao et al., 2023). Based on their structures and functions in *A. thaliana*, the BBX genes have been divided into five subfamilies (groups I, II, III, IV, and V) that are closely associated with a number of B-box and CCT conserved domains (Gangappa and Botto, 2014). Groups I and II contain two B-box domains (B-box1 and B-box2) and one CCT domain, group III contains a B-box1 domain and a CCT domain, group IV contains two B-box domains but no CCT domain, whereas group V only contains a B-box1 domain (Khanna et al., 2009). Hence, addressing the diversity of the BBX family may be conducive to understand its roles.

Increasing evidence has demonstrated that BBX is involved in various developmental processes and in response to environmental stresses (Cao et al., 2023). In Chinese yam (*D. opposita*), overexpression of *DoBBX2* and *DoBBX8* accelerated tuber formation under short-day conditions (8-h photoperiod; light intensity: 38 μmol m^-2^ s^-1^) while overexpression of *DoBBX8* alone promoted tuber formation in the dark (Chang et al., 2023). In tomato (*Solanum lycopersicum*), SlBBX17 interacted with SlHY5 and stimulated transcription of the *SlHY5* gene, positively enhanced CBF-dependent cold tolerance, whereas silencing of *SlBBX17* promoted susceptibility to low-temperature stress (Song et al., 2023). The expression of 68.4% of *D. officinale BBX* genes increased to varying degrees after treatment with methyl jasmonate (MeJA), especially *DoBBX17*, which was strongly up-regulated (by 65-fold) after 24 h (Cao et al., 2019). In apple (*Malus domestica*), MdBBX20, induced by UV-B and low temperatures, interacted with MdHY5 to activate *MdMYB1* expression, binding directly to the promoters of *MdDFR* and *MdANS* genes, and promoting the accumulation of anthocyanins (Fang et al., 2019). In pear (*Pyrus pyrifolia*), the expression of *PpBBX16* was significantly enhanced under constant white light (light intensity: 13.19 µmol m^-2^ s^-1^) induction for 10 d, and strongly interacted with PpHY5 to induce the promoter activity of *PpMYB10*, while integrating the well-characterized COP1-HY5-MYB10 regulatory complex, which is involved in the determination of red coloration and anthocyanin biosynthesis (Bai et al., 2019a). Despite these studies in other plants, no information regarding the BBX family is available for the medicinally edible plant, *Rubus chingii* Hu (Rosaceae).

*R. chingii*, commonly known as hanging hooks in English, acquired its name due to the shape of ripe fruits, which resemble an upside-down hanging bowl on branches. *R. chingii* is used in the Chinese pharmacopoeia, and is widely cultivated in Zhejiang and Jiangxi provinces of China. Not only is it consumed as a fresh fruit when ripe, its unripe dried fruit serves as a medicinal herb (Wang et al., 2022). Ripening *R. chingii* fruit undergoes a change in color from green to red, and this is dependent on relevant TFs that can regulate the biosynthesis and accumulation of anthocyanins (Li et al., 2021). For instance, BBX can directly or indirectly regulate anthocyanin biosynthetic genes at transcriptional and post-transcriptional levels, thereby adjusting the levels of anthocyanin production (Wang et al., 2023). In *A. thaliana* seedlings, overexpression of *BBX21*, *BBX22* and *BBX23* accumulated dramatically more anthocyanin than control seedlings when induced by light, while *BBX24*, *BBX25* and *BBX32* negatively regulated the accumulation of anthocyanins (Job et al., 2018; Bai et al., 2019b; Podolec et al., 2022). Furthermore, *R. chingii* fruit has an abundance of flavonoids, terpenoids, alkaloids, and phenolics (Wang et al., 2021). Collectively, these exhibit a wealth of pharmacological properties, including antibacterial, antioxidant, anti-tumor, and anti-inflammatory (Chen, 2023). The accumulation of these secondary metabolites is potentially related to induction by phytohormones, and may be transcriptionally regulated by the BBX family (Gangappa and Botto, 2014; Song et al., 2020). The expression profiles of the BBX family genes in *R. chingii* are still unknown.

The objective of the present study was to identify the BBX family from *R. chingii* at the genome-wide level. The physicochemical properties, chromosome location, gene structure, phylogenetic relationships, analysis of *cis*-acting elements (CAEs), conserved motifs, gene replication, and protein-protein interactions were systematically evaluated. Furthermore, the expression levels of BBX members in different organs (roots, stems, leaves, flowers, and fruits), at different developmental stages, and following exposure to abscisic acid (ABA), were comparatively evaluated. Consequently, these findings will allow candidate genes to be identified while providing a theoretical foundation for understanding the role of BBX family genes during the ripening of *R. chingii* fruit.

## 2 Materials and methods

### 2.1 Plant materials and hormonal treatment

The erect and medium-sized shrub *R. chingii* Hu, which was identified as such by Professor Xiaoxia Shen (Zhejiang Chinese Medical University), was cultivated in a greenhouse using sandy and clay soil (4:1, v/v) and irrigated every fortnight with 1000-times diluted Hyponex 20-20-20 fertilizer (Hyponex Co., Tokyo, Japan) under natural conditions at the medicinal herb garden (*E* 119.96°, *N* 30.05°) of Zhejiang Chinese Medical University, in Zhejiang Province, China. To detect the organ-specific expression of *RcBBX* genes, roots, stems, leaves, flowers, and unripe fruits of one-year-old *R. chingii* plants were collected in May 2023, quick-frozen in liquid nitrogen, and stored in a -80°; refrigerator (Thermo Scientific, Waltham, MA, USA). To investigate the dynamic expression of *RcBBX* genes during fruit ripening, small green (5-6 mm in diameter), big green (11-13 mm in diameter), yellow and red fruits were harvested. Their surfaces were wiped clean with a dry cloth, then stored in a -80°; refrigerator. To understand the effect of ABA on the transcript levels of *RcBBX* genes, ten leaves of one-year-old *R. chingii* plants from top to bottom were sampled after treatment with 100 μM ABA (Sigma-Aldrich, St. Louis, MO, USA) 10 mL for each plant over 6 h, then stored in a -80°; refrigerator. Triplicate samples were prepared.

### 2.2 Identification of *R. chingii* BBX family members at the genome-wide level

The chromosome-scale genome of *R. chingii* was retrieved from the Rosaceae Genome Database (https://www.rosaceae.org) with accession number tfGDR1051. A BLASTP search (score value ≥ 100, e-value ≤ 1e-10) was employed to identify all possible *R. chingii* BBX members by utilizing the 32 published *A. thaliana* BBX proteins (Khanna et al., 2009) as queries. Each candidate BBX was subsequently verified with the Conserved Domain Database (CDD, www.ncbi.nlm.nih.gov/cdd) and Simple Modular Architecture Research Tool (SMART, https://smart.embl-heidelberg.de/). Redundant sequences or incomplete sequences, excluding the conserved BBX domain (PF00643) which was retrieved from Pfam (https://pfam-legacy.xfam.org/), were excluded. Consequently, all 32 RcBBX members with complete BBX domains were identified (**Supplementary Table 1**). In addition, physical and chemical properties of all RcBBX proteins, including their molecular weight, theoretical isoelectric point (pI), instability index, aliphatic index, and grand average of hydrophobicity, were investigated with the ExPASy server (https://www.expasy.org). The subcellular localization of all RcBBX members was predicted using the WoLF server (https://wolfpsort.hgc.jp/) and the Plant-mPLoc predictor (Chou and Shen, 2010).

### 2.3 Phylogenetic analysis of RcBBX members and multiple sequence alignment

The *A. thaliana* BBX sequences were identified at the *Arabidopsis* Information Resource (https://www.arabidopsis.org/). The full-length amino acid sequences of the BBX proteins from *A. thaliana* and *R. chingii* were subjected to multiple sequence alignment using ClustalX 2.1 (www.clustal.org/). A phylogenetic tree was established using Molecular Evolutionary Genetics Analysis (MEGA) software (Tamura et al., 2021) applying a neighbor-joining (NJ) algorithm (Saitou and Nei, 1987) with 1000 bootstrap replicates, and visualized using the iTOL online tool (https://itol.embl.de).

### 2.4 *Cis*-acting elements, gene structure, and conserved motifs of RcBBX members

The 2000-bp long upstream sequences of the 32 *RcBBX* genes were mined from the *R. chingii* genome (Wang et al., 2021), sorted in numerical order and inputted into the PlantCARE platform (Lescot et al., 2002) to predict the potential CAEs. According to the annotation information of the *R. chingii* reference genome (Wang et al., 2021), the structure of *RcBBX* genes was revisualized with the TBtools program (Chen et al., 2020). To better understand the structure of these RcBBX members, the Multiple Expectation Maximization for Motif Elicitation website (MEME, https://meme-suite.org/) was employed to determine their conserved motifs (motif width: 6-50; max number: 10), and visualized with TBtools software (Chen et al., 2020).

### 2.5 Chromosome distribution, duplications and synteny analysis of RcBBX members

Based on the relative positions of the 52 characterized *RcBBX* genes on the seven *R. chingii* chromosomes, the TBtools program (Chen et al., 2020) was applied to map them on the chromosomes. To explore gene replication events among *RcBBX* genes, collinearity analysis of RcBBX members was performed using the multiple collinear scanning toolkit MCScanX (Wang et al., 2012) with default parameters. The Dual Synteny Plotter in TBtools tool (Chen et al., 2020) was used to carry out a syntenic analysis of three BBX gene families in *R. chingii*, *A. thaliana*, and rice (*O. sativa*).

### 2.6 Identification of enzyme-encoding genes related to anthocyanidin biosynthesis

The anthocyanidin biosynthetic pathway in *R. chingii* was putatively implicated based on an assessment of the Kyoto Encyclopedia of Genes and Genomes (KEGG) pathway database (Kanehisa et al., 2023) and previously published reports regarding flavonoid biosynthesis (Li et al., 2021; Lei et al., 2023). Finally, information about the transcriptome (NCBI accession no. PRJNA671545) of *R. chingii* fruits during different developmental stages (small green, big green, yellow, and red fruits) were retrieved to appreciate the enzyme-encoding genes involved in the biosynthesis of flavonoids. The TBLASTN server (https://blast.ncbi.nlm.nih.gov/Blast.cgi) was employed to align homologous genes against the enzyme-encoding genes. Redundant and incomplete sequences without functional domains were removed. Finally, enzyme-encoding genes were selected from the *R. chingii* transcriptome database when their FPKM values exceeded 5.0.

### 2.7 GO and KEGG enrichment analysis of RcBBX sequences

All RcBBX sequences were uploaded into the eggNOG-mapper website (https://eggnog-mapper.embl.de/). The functionally annotated results were downloaded, selected with a *P*-value less than 0.05, and the GO and KEGG enrichment information was visualized on the Chiplot server (https://www.chiplot.online/).

### 2.8 Expression profiles of *RcBBX* genes in different organs, at different growth stages, and following treatment with ABA

Total RNA was isolated from the lyophilized powder of different organs (roots, stems, leaves, flowers, and fruits), different developmental fruits (small green, big green, yellow and red fruits), and ABA-treated *R. chingii* leaves, using the Quick RNA Isolation Kit (Huayueyang Biotechnology Co., Beijing, China) according to the company’s instruction manual (Yu et al., 2021a). Thereafter, the quality and integrity of RNA were estimated using agarose gel electrophoresis (Bio-Rad Laboratories, Hercules, CA, USA) and a NanoDrop 2000 spectrophotometer (Thermo Scientific). The acquired 1 μg of RNA was reverse-transcribed to first-strand cDNA using the PrimeScript Reagent Kit with gDNA Eraser (Takara, Dalian, China). Real-time quantitative polymerase chain reaction (qRT-PCR) was implemented on an ABI 7500 (Applied Biosystems, Foster City, CA, USA) at the following conditions: 95°C for 30 s, followed by 35 cycles of 95°C for 2 s, and 60°C for 30 s. A total of 10 μL detection solution included 5 μL of 2× iTaq™ Universal SYBR^®^ Green Supermix (Bio-Rad Laboratories), 50 ng of obtained cDNA, 500 nM of forward/reverse primer, and the remaining volume with deionized water. Relative transcript abundance was assessed based on the 2^-ΔΔCt^ protocol (Livak and Schmittgen, 2001), and the elongation factor-1 alpha gene (*EF-1α*) was employed as the house-keeping gene (Wang et al., 2021). All primers that were used are indicated in **Supplementary Table 1**.

### 2.9 Molecular cloning, sequence analysis and subcellular localization of RcBBX26

The sequences encoded by *RcBBX26* were propagated and purified from *R. chingii* fruits using the ApexHF HS DNA Polymerase FS (Accurate Biotechnology (Hunan) Co., Ltd., Changsha, China). The secondary structure of RcBBX26 was visualized at the SOPM webserver (https://npsa-pbil.ibcp.fr). The subcellular localization of RcBBX26 was assessed as described previously (Yu et al., 2021a), NLS-mCherry was used as a nuclear localization marker, and YFP fluorescence was observed and images were captured under a TCS SP8 STED microscope (Leica Camera AG, Solms, Germany).

### 2.10 Transient overexpression of *RcBBX26* in *R. chingii* leaves

To induce gene overexpression, a 765-bp coding sequence of *RcBBX26* without a stop codon (TGA) was introduced into the pCAMBIA1301 vector (CAMBIA, Canberra, Australia) at the *EcoR*I and *BamH*I sites using the In-Fusion solution (Takara). After verification by sequencing (Zhejiang SUNYA Co., Hangzhou, China), the pCAMBIA1301-RcBBX26 recombinant was introduced into *Agrobacterium tumefaciens* GV3101 (pSoup-p19; Weidi Biotechnology Co., Shanghai, China) using a previously published freeze-thaw protocol (Yu et al., 2021b). Infiltration solution, containing 10 mM 2-morpholinoethanesulfonic acid (MES; Sigma-Aldrich), 10 mM MgCl_2_ (Sigma-Aldrich), and 20 mM acetosyringone (Sigma-Aldrich) (pH = 5.6) was injected into the third leaves from the terminus of one-year-old *R. chingii* plants. Total RNA was isolated from MOCK and overexpressing *RcBBX26* leaves as mentioned above. Semi-quantitative RT-PCR was performed following thermocycling as initially published (Yu et al., 2021b). Eventually, positive leaves overexpressing *RcBBX26* were verified by semi-quantitative RT-PCR and qRT-PCR as indicated above.

### 2.11 Determination of total anthocyanidin content during fruit ripening

To investigate total anthocyanidin content during fruit ripening, four developmental fruits (small green, big green, yellow and red fruits) of one-year-old greenhouse *R. chingii* were harvested in May 2023. Anthocyanin content in different *R. chingii* fruits was determined by a pH differential protocol (Yu et al., 2018). Dried powder (1 g) derived from a vertical-type pulverizer (Xinda Machinery Co., Jiangyin, China) was mixed with 2 mL methanol (Sigma-Aldrich), mixed with 1.0% formic acid (Sigma-Aldrich), and extracted by ultrasonication with a 40 Hz ultrasonic homogenizer (Ningbo Scientz Biotechnology Co., Ningbo, China) in the mixture of ice and water for 15 min. The supernatant was obtained after centrifugation (4000 ×*g*) at 25°. Anthocyanidin content was spectroscopically evaluated at 510 and 700 nm in separate acidic buffers at pH 1.0 and 4.5, respectively. The formula utilized to quantify anthocyanin was: A = (A_510nm_ – A_700nm_) _pH1.0_ – (A_510nm_ – A_700nm_) _pH4.5_. Cyanidin 3-*O*-rutinose (CAS no. 18719-76-1; Sigma-Aldrich) was used as the reference standard to establish a standard curve (A = 2.3016C + 0.021, *R²*=0.9991; C indicates the concentration of cyanidin 3-*O*-rutinose). Total anthocyanidin content of different *R. chingii* fruits was expressed as mg of cyanidin 3-*O*-rutinose equivalents per gram of dry weight (DW).

### 2.12 Statistical analysis

All utilized data are presented as the mean ± standard deviation (SD) of no less than three independent replicates. Statistical analysis was executed using SPSS Statistics version 22.0 (IBM Corp., Armonk, NY, USA). In graphs, asterisks above columns indicate statistical differences in expression abundance between CK and ABA, as assessed by a student’s *t*-test at *P* < 0.01. Furthermore, different lowercase letters above columns indicate significant differences of anthocyanin content among different fruit ripening stages (small green, big green, yellow and red fruits), as assessed by Duncan’s multiple range test at *P* < 0.01. Significant differences between control and treated group were evaluated by student’s *t*-test (*P* < 0.01). Heatmaps of the expression abundance of all *RcBBX* genes at different tissues, or at different ripening stages, were charted using TBtools software (Chen et al., 2020), and color scales indicate the log2-transformation of average expression levels, with high expression noted by red/orange and low expression indicated by blue/cyan. A correlation of expression abundance between all *RcBBX* genes and anthocyanidin biosynthetic genes was implemented using Pearson’s correlation coefficient (*r*) at *P* < 0.05.

## 3 Results

### 3.1 Identification and characterization of RcBBX protein family members

To mine BBX members on the seven chromosomes in the *R. chingii* genome, local Hidden Markov Model searches with the BBX core domain (PF00643) and a BLAST with all 32 A*. thaliana* BBX proteins as queries were carried out. The obtained BBX proteins were confirmed using the Simple Modular Architecture Research Tool and an NCBI CD-Search. Thereafter, redundant or erroneous sequences without PF00643 were removed. A total of 32 BBX members were finally identified from the *R. chingii* genome and sequentially named RcBBX1-RcBBX32 according to their chromosomal positions (**Table 1**; **Supplementary Table 2**).

**Table 1:** Physicochemical properties of RcBBX proteins in *R. chingii*.

Name	Length (aa)	Molecular weight (kD)	pI	Instability index	Aliphatic index	Grand average of hydropathicity	Subcellular localization
RcBBX1	687	75.16	5.32	53.25	60.36	-0.669	Nucleus
RcBBX2	686	75.04	5.32	54.25	62.16	-0.648	Nucleus
RcBBX3	186	20.63	6.49	53.27	70.27	-0.559	Cytosol
RcBBX4	763	82.15	8.61	67.41	59.34	-0.617	Nucleus
RcBBX5	255	29.10	8.68	59.00	72.98	-0.552	Nucleus
RcBBX6	809	88.52	6.61	58.20	58.92	-0.662	Nucleus
RcBBX7	385	42.58	5.62	54.36	57.06	-0.679	Cytosol
RcBBX8	339	37.73	5.82	43.34	70.47	-0,463	Nucleus
RcBBX9	302	32.91	5.83	51.42	69.47	-0.344	Nucleus
RcBBX10	215	24.07	5.98	44.61	70.28	-0,470	Nucleus
RcBBX11	265	30.03	7.10	66.47	73.51	-0.450	Nucleus
RcBBX12	285	30.29	4.72	52.52	64.04	-0.235	Nucleus
RcBBX13	248	27.78	4.40	58.52	58.19	-0.955	Nucleus
RcBBX14	198	21.88	4.06	70.49	38.38	-1.221	Nucleus
RcBBX15	223	25.39	8.69	75.07	68.61	-0.638	Nucleus
RcBBX16	866	95.32	7.57	37.66	89.36	-0.344	Nucleus
RcBBX17	239	26.38	4.75	47.99	75.98	-0.307	Nucleus
RcBBX18	239	26.32	4.76	48.93	76.82	-0.286	Nucleus
RcBBX19	197	23.29	9.45	56.63	70.25	-0.650	Chloroplast
RcBBX20	348	38.16	6.70	47.09	71.47	-0.331	Chloroplast
RcBBX21	432	47.96	5.41	54.68	58.08	-0.749	Chloroplast
RcBBX22	501	56.05	5.92	62.28	65.19	-0.577	Nucleus
RcBBX23	610	68.01	6.10	47.35	69.77	-0.610	Nucleus
RcBBX24	417	45.18	5.43	55.83	58.99	-0.509	Nucleus
RcBBX25	173	18.89	7.04	37.34	83.99	-0.054	Cytosol
RcBBX26	254	27.40	8.61	61.05	74.17	-0.158	Chloroplast
RcBBX27	211	23.85	8.72	62.78	65.12	-0.609	Nucleus
RcBBX28	251	28.14	8.55	54.10	82.03	-0.301	Nucleus
RcBBX29	400	43.98	5.52	36.80	59.80	-0.605	Nucleus
RcBBX30	265	28.99	4.57	57.31	56.04	-0.985	Nucleus
RcBBX31	434	47.38	5.10	58.27	67.19	-0.506	Chloroplast
RcBBX32	243	27.06	8.85	61.86	82.26	-0.190	Chloroplast

The RcBBX proteins ranged from 173 aa (RcBBX25) to 866 aa (RcBBX16) in length, and their molecular weight ranged from 18.89 to 95.32 kDa. The pI ranged from 4.06 (RcBBX14) to 9.45 (RcBBX19), similar to the pIs of BBX proteins from soybean (Shan et al., 2022) and grapevine (Wei et al., 2020). The number of unstable proteins was high (29/32), suggesting that these RcBBX proteins, with an instability index > 40, exhibited low overall stability. The grand average of hydropathicity of RcBBX proteins was less than 0, suggesting that they are hydrophilic. Most of the RcBBX proteins (23/32) were located in the nucleus, although some of them were predicted to be located in the cytosol or chloroplast, indicating that these proteins might function as TFs.

### 3.2 Phylogenetic analysis of RcBBX protein family members

**Figure 1:**
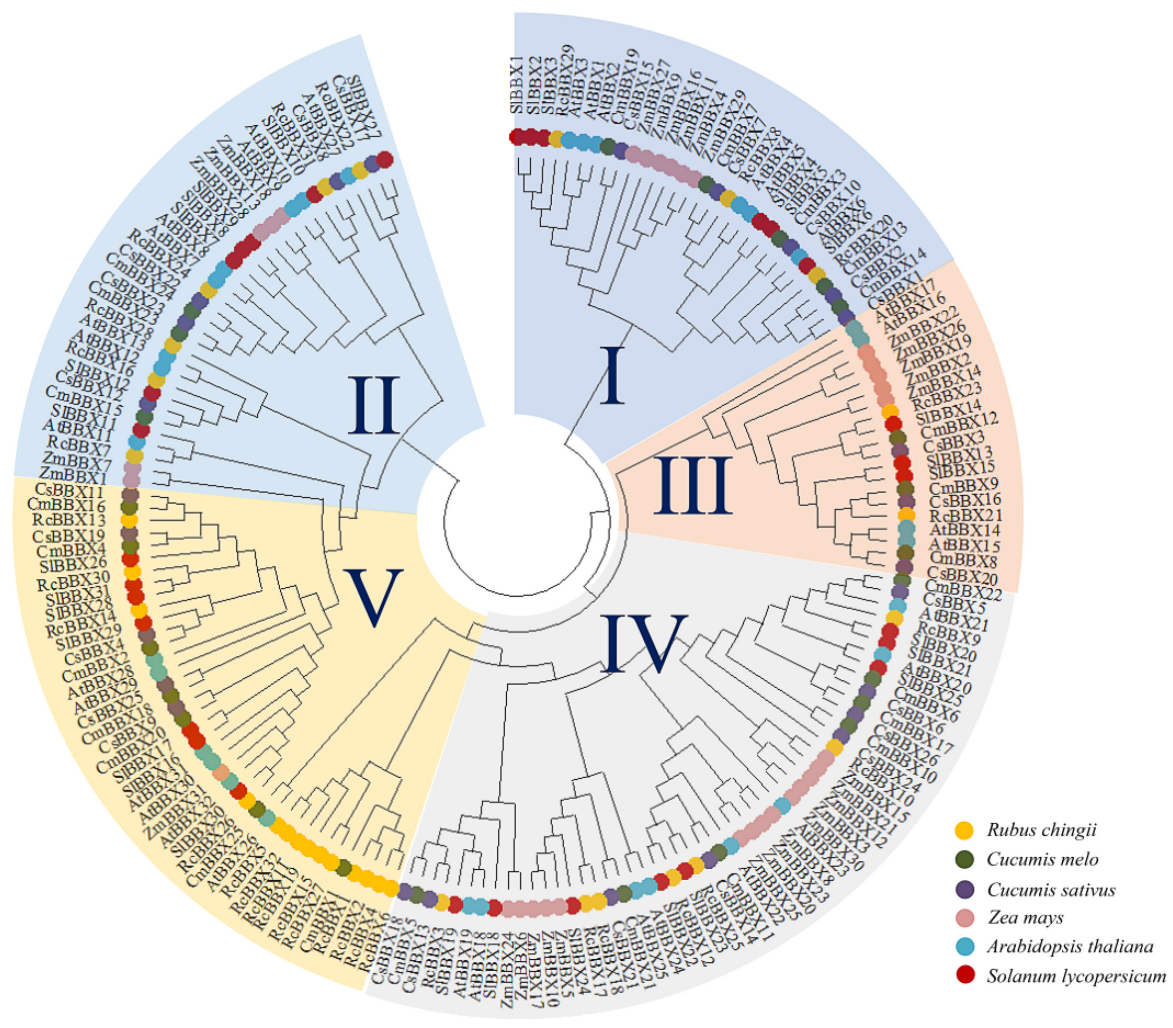
Phylogenetic tree of 177 BBX protein family members in *A. thaliana*, *C. melo*, *C. sativus*, *R. chingii*, *S. lycopersicum*, and *Z. mays*. The phylogenetic tree was constructed with MEGA software using a maximum likelihood approach with 1000 bootstrap replications.

To explore the classification and evolution of these RcBBX family members, the 32 identified RcBBX proteins, together with 32 A*. thaliana* AtBBX proteins, 26 *Cucumis melo* CmBBX proteins, 26 C*. sativus* CsBBX proteins, 30 *Solanum lycopersicum* SlBBX proteins, and 31 *Zea mays* ZmBBX proteins (**Supplementary Table 3**), were sequentially aligned to assess their phylogenetic relationships (**Figure 1**). According to the phylogenetic analysis, and confirming a prior classification of BBX members into five subfamilies (Gangappa and Botto, 2014), these BBX members were divided into five groups (groups I, II, III, IV, and V), including 3, 6, 2, 7, and 14 RcBBX members (**Supplementary Table 4**), respectively. Among them, group II was the smallest subgroup, including 20 BBX proteins, whereas group IV had the most BBX proteins (52).

### 3.3 Chromosomal location and gene duplication analysis of all *RcBBX* genes

**Figure 2:**
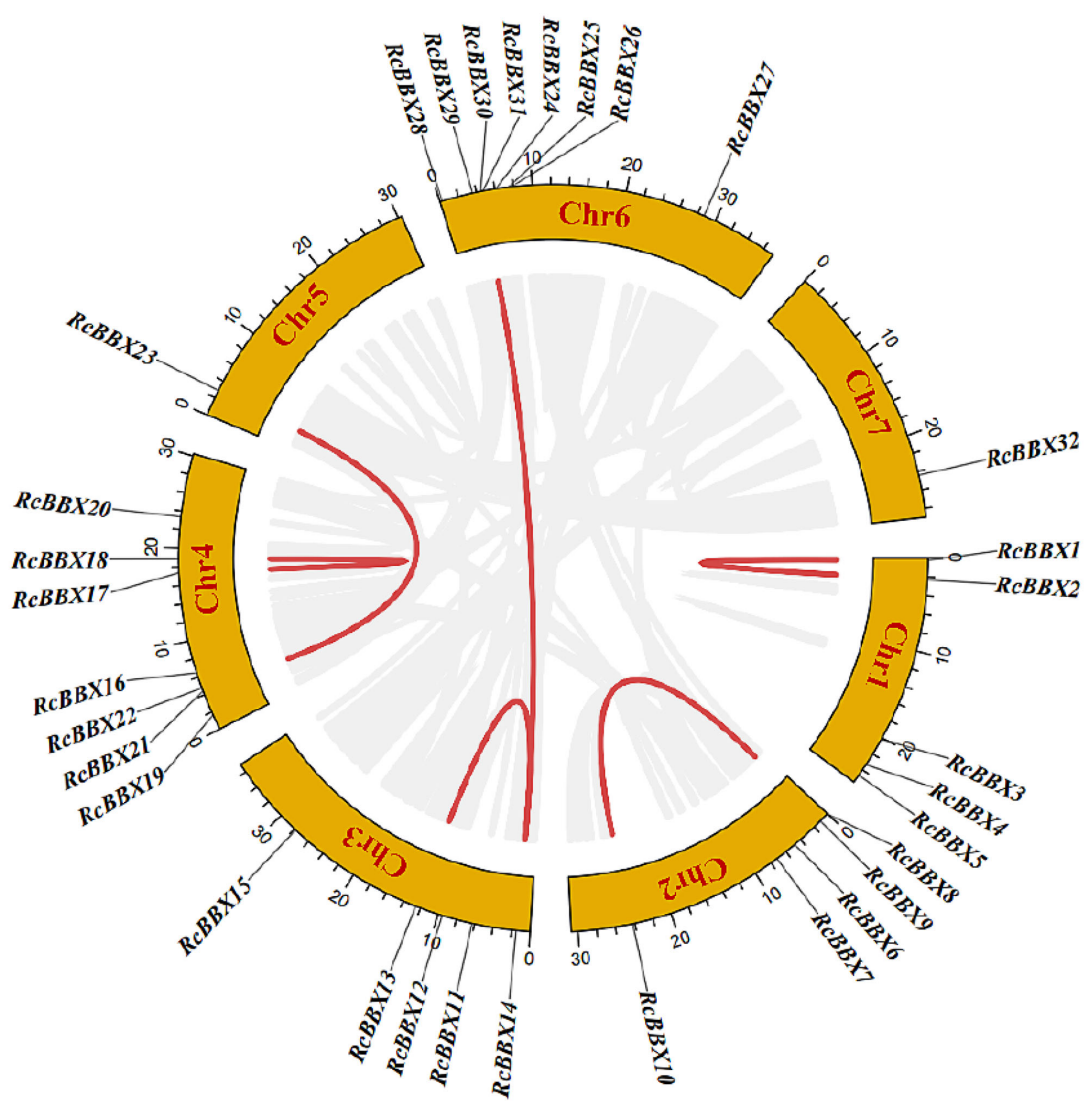
Chromosomal location and collinearity analysis of the *RcBBX* genes.

The genomic location of the 32 *RcBBX* genes revealed that they were unevenly mapped across all seven chromosomes (**Figure 2**). Eight *RcBBX* genes were distributed on chromosome 6, exhibiting the highest number of *RcBBX* members. In addition, seven genes were distributed on chromosome 4, while chromosomes 1, 2, and 3 included five *RcBBX* genes each. Chromosomes 5 and 7 contained only one gene each, *RcBBX23* and *RcBBX32*, respectively.

**Figure 3:**
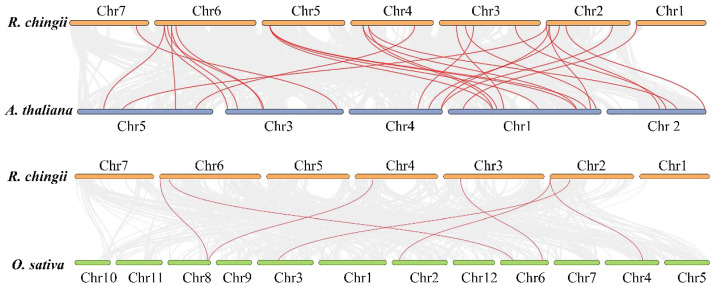
Collinearity analysis of *BBX* genes among *A. thaliana*, *O. sativa*, and *R. chingii*.

Duplication analysis demonstrated that six gene pairs (*RcBBX1*-*RcBBX2*, *RcBBX9*-*RcBBX10*, *RcBBX13*-*RcBBX14*, *RcBBX17*-*RcBBX18*, *RcBBX21*-*RcBBX23*, *RcBBX14*-*RcBBX29*) were defined as tandemly-duplicated segments at the chromosome level (**Figure 2**). Furthermore, four pairs of gene duplications occurred within the same chromosomes (1, 2, 3, and 4) while another two (*RcBBX14*-*RcBBX29*, *RcBBX21*-*RcBBX23*) occurred on two different chromosomes (3 and 6, 4 and 5). In addition, collinearity analysis of *BBX* genes among *R. chingii*, *A. thaliana*, and *O. sativa* was sequentially executed (**Figure 3**). Comparative syntenic maps of the *R. chingii* genome with the genomes of two model plants *A. thaliana* and *O. sativa*, indicated 28 homologous gene pairs between *R. chingii* and *A. thaliana* on 7 chromosomes, and seven homologous gene pairs between *R. chingii* and *O. sativa* on 4 chromosomes.

### 3.4 Conserved domains, motifs and gene structure analysis of RcBBX protein family members

**Figure 4:**
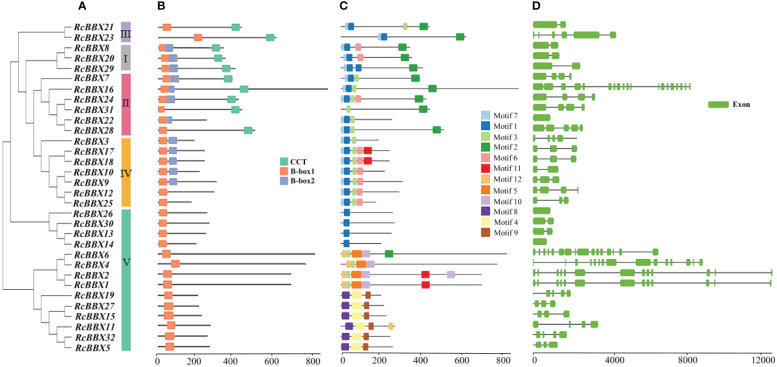
Phylogenetic tree, structural domains, conserved motifs, and gene structure of the 32 *RcBBX* members. **(A)** Phylogenetic classification of the 32 RcBBX proteins. **(B)** Structural domains of the 32 RcBBX proteins. **(C)** Conserved motifs of the 32 RcBBX proteins. **(D)** Gene structure of the 32 *RcBBX* genes.

Apart from RcBBX28 and RcBBX31, nine RcBBX proteins in groups I and II included two B-box structural domains and one CCT structural domain (**Figure 4A**). Two RcBBX proteins in group III included one B-box and one CCT domain. Most RcBBX proteins (5/7) in group IV contained two B-box structural domains. All RcBBX proteins in group V included only one B-box structural domain (**Figure 4B**). Therefore, RcBBX proteins within the same subgroup exhibited similar domain compositions.

Furthermore, 12 motifs were observed and named as motifs 1-12 (**Figure 4C**). Except for 10 proteins in group V, motif 1 was widely distributed in all other RcBBX members. Motif 7 was distributed in groups I, II, III, and IV. Of note, the distribution of motifs in group V was divided into three types: type 1 (4 members), which only included motif 1; type 2 (4 members), which mainly included motifs 2, 3, 5, and 10; type 3 (6 members), which included motifs 4, 8, and 9. Overall, the number and types of motifs within the same group were relatively similar, although there were considerable differences among different groups.

The CDS/intron patterns of *RcBBX* genes were also analyzed (**Figure 4D**). The number of exons in groups I, II, III, and IV was 1-6 (except for 19 exons in *RcBBX16*). Most *RcBBX* genes harbored 1-6 exons in group V whereas five genes (*RcBBX1*, *RcBBX2*, *RcBBX4*, *RcBBX6*, *RcBBX1*6) possessed 13-15 exons. Intriguingly, the number of exons was relatively conserved within the same group, for example, group IV genes contained 2-5 exons.

### 3.5 *Cis*-acting element analysis of the promoter region of *RcBBX* genes

**Figure 5:**
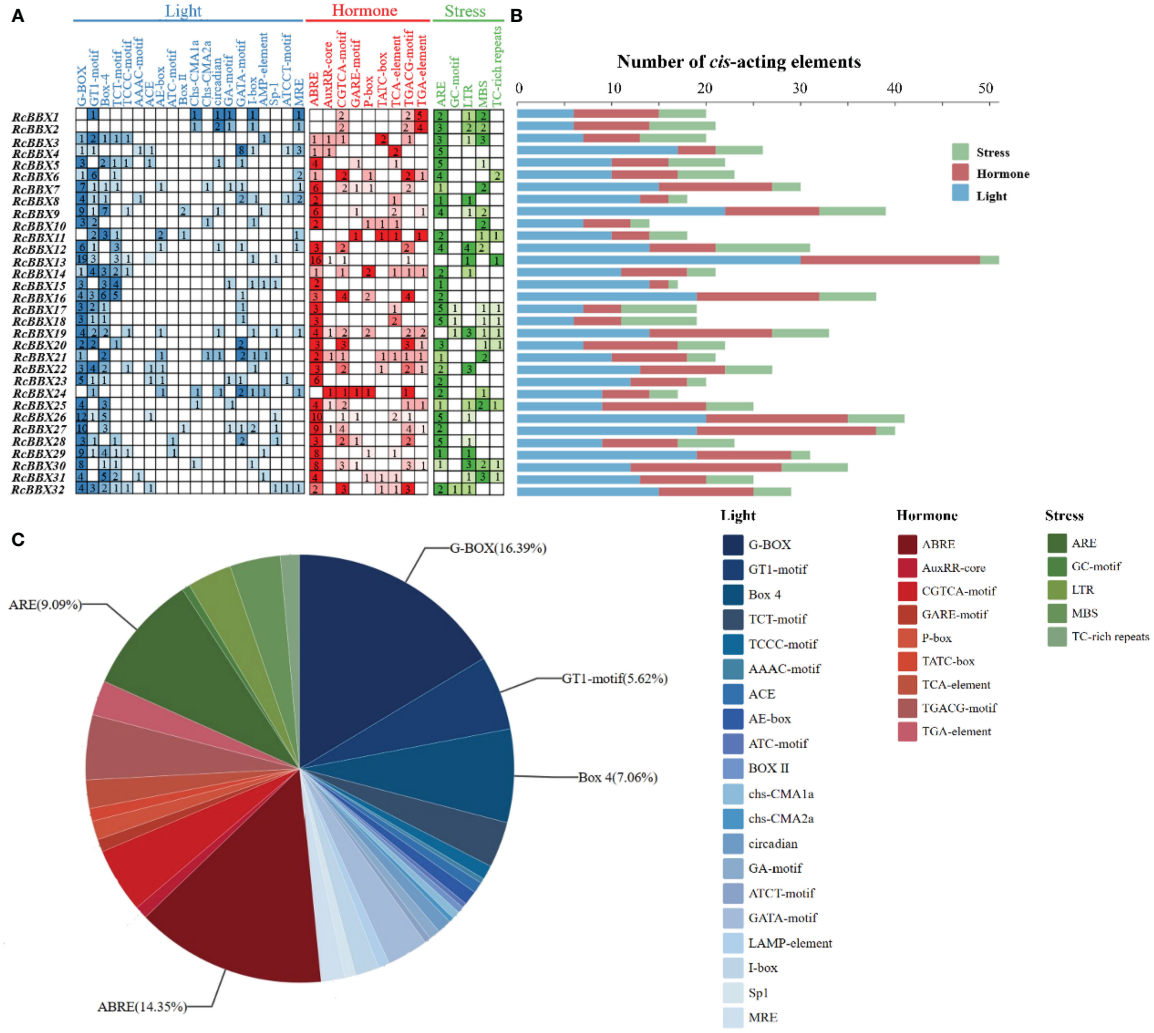
Analysis of *cis*-acting elements in the *RcBBX* genes. **(A)** All *cis*-acting elements were classified into three categories, namely those associated with light, hormone, and stress, and their number is presented as a heatmap. **(B)** Number of *cis*-acting elements in each *RcBBX* gene. **(C)** Histogram of the number of *cis*-elements in each category.

The 2000-bp upstream promoter of the 32 *RcBBX* genes was utilized to analyze the CAEs, which were divided into three categories: those associated with light, hormone, and stress (**Figure 5**). A total of 836 CAEs were identified, and 405 of them were associated with light responsiveness. For instance, the G-box, a common CAE in plants exposed to external light stimulation, was the most widely distributed CAE, accounting for 33.83% of all light-responsive CAEs. *RcBBX13* contained the most G-boxes (19), followed by *RcBBX26* (12) and *RcBBX27* (10). The I-box was found in 3.7% of all light-responsive CAEs, and together with the G-box, formed a key component with conserved modular array 5 (CMA5) for the regulation of photosensitive pigments. A total of 278 hormone-responsive CAEs were discovered. ABRE, an ABA-responsive element, was the most widely distributed and accounted for 43.17% of all hormone-responsive CAEs. Most *RcBBX* genes (23/32) contained an ABRE, indicating that they were potentially induced by ABA. Similar to light responsiveness, *RcBBX13* contained the most ABREs (16), followed by *RcBBX26* (10) and *RcBBX27* (9). CGTCA and TGACG were MeJA-responsive elements, accounting for 29.5% of all hormone-responsive CAEs. In addition, there were 153 CAEs involved in stress response. ARE and GC motifs, two anaerobic inducible CAEs, were the most common, accounting for 52.94% of all stress-responsive elements. Multiple CAEs were associated with stress responsiveness, such as MBS and LTR, which were involved in drought and low temperature responsiveness (Yu et al., 2021b; Nian et al., 2022), respectively. Hence, it is noteworthy that the promoter regions of each *RcBBX* gene exhibited a different number and composition of CAEs, and might play multifarious roles in photomorphogenesis and stress resistance in *R. chingii*.

### 3.6 Protein-protein interaction network of RcBBX proteins

**Figure 6:**
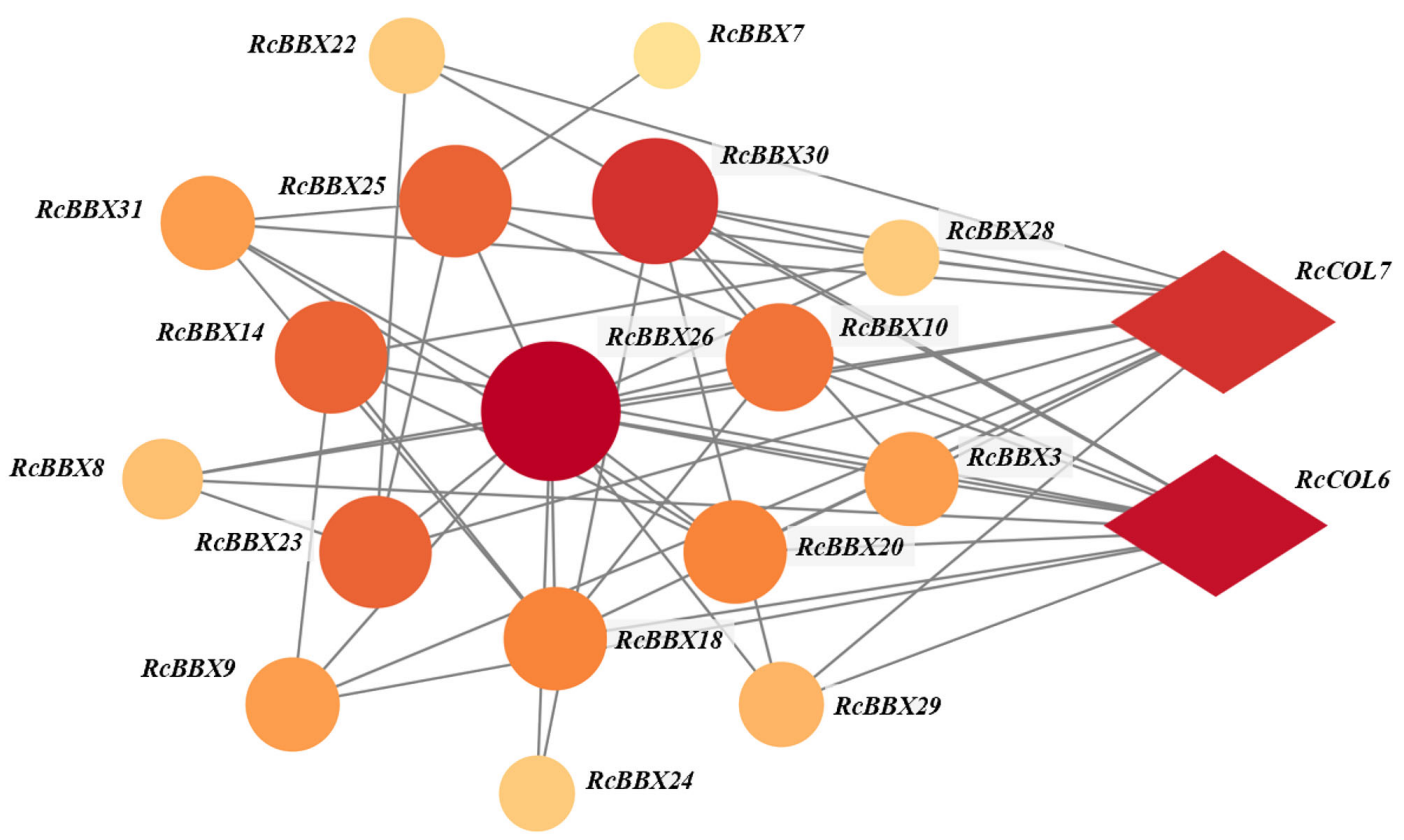
Interactive networks of RcBBX proteins assessed with STRING software.

To depict the protein-protein interaction of all RcBBX proteins, the STRING database was employed. Results demonstrate that 24 RcBBX proteins were predicted as being homologous to *A. thaliana* BBX proteins whereas seven other proteins (RcBBX2, RcBBX4, RcBBX6, RcBBX11, RcBBX19, RcBBX27, and RcBBX32) exhibited relatively low connectivity (**Figure 6**). Among the 17 interacting proteins, the amount of connectivity ranged from 1-12, and the most highly connected protein was RcBBX26, which interacted with 16 other RcBBX proteins. In addition, 2 CONSTANS-like (COL), RcCOL6 and RcCOL7, also interacted with RcBBX proteins.

### 3.7 GO and KEGG enrichment annotation of RcBBX proteins

**Figure 7:**
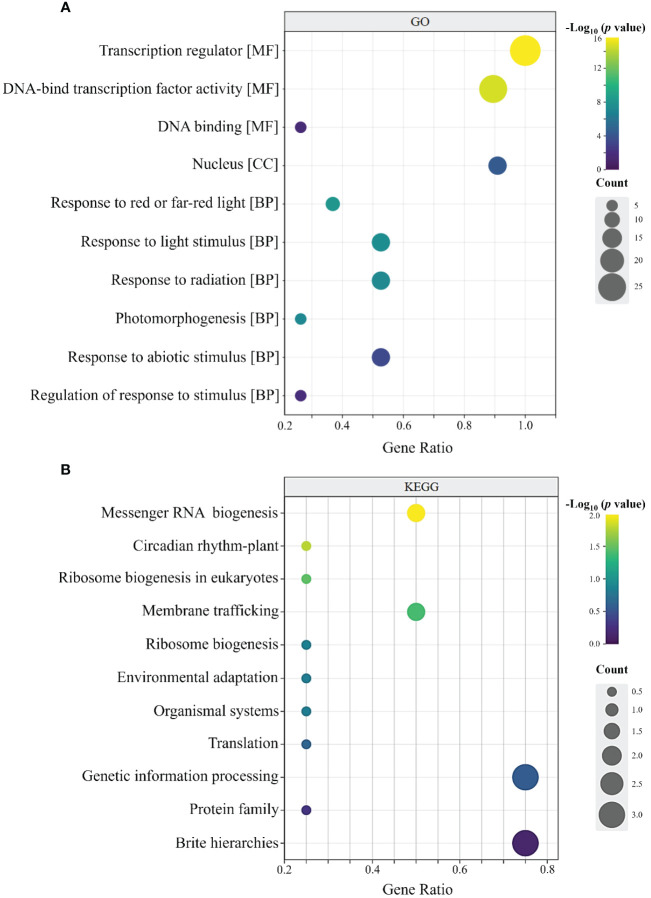
GO and KEGG annotation of *RcBBX* proteins. **(A)** GO enrichment analysis of 32 *RcBBX* proteins. All annotated GO terms including biological processes (BP), cellular components (CC) and molecular functions (MF). **(B)** KEGG enrichment analysis of 32 *RcBBX* proteins.

As shown in **Figure 7A**, the 32 RcBBX members had 74 annotated GO terms, and they were categorized into three types, including 66 molecular functions (MF), five cellular components (CC), and one biological process (BP). In the MF group, RcBBX proteins were mainly associated with the activity of a transcription regulator or a DNA-binding TF, which can activate or repress the expression levels of downstream structural genes, playing an important regulatory role in plant growth, development and defense against stresses. In the CC group, the RcBBX proteins were enriched in the nucleus, inferring that they might be involved in nuclear transcription. In the BP group, RcBBX proteins showed significant enrichment in response to light and abiotic stress stimuli, indicating they were linked to photo- and bio-stimulation.

KEGG annotation revealed that RcBBX proteins were frequently linked to protein families associated with the processing of genetic information (**Figure 7B**). Moreover, RcBBX proteins were associated with environmental adaptations, concurrent with the BP group in GO enrichment.

### 3.8 Tissue-specific expression levels of 32 *RcBBX* genes

**Figure 8:**
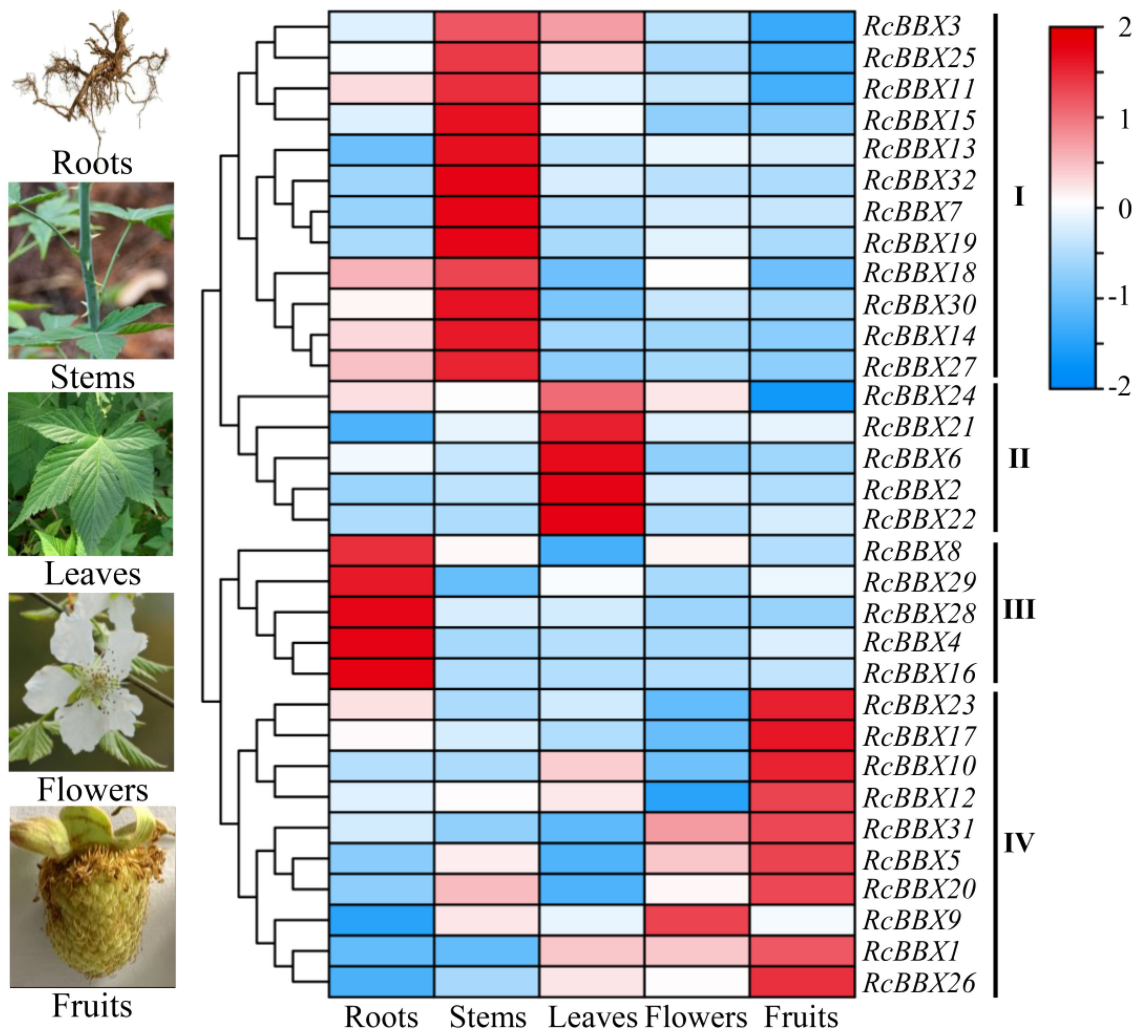
Expression profiles of 32 *RcBBX* genes in flowers, fruits, leaves, roots, and stems of *R. chingii*.

To appreciate the expression patterns of *RcBBX* genes in various tissues, expression levels in flowers, fruits, leaves, roots, and stems were determined (**Figure 8**). *RcBBX* genes exhibited differential expression in these five tissues, divided into four groups (I, II, III, and IV). There were 12 *RcBBX* genes in group I, with the highest expression in stems. Group II contained five *RcBBX* genes, which displayed the highest expression in leaves. Group III also consisted of five *RcBBX* genes, with the highest expression in roots. The remaining 10 genes were clustered into group IV, and most of them (9/10) displayed highest expression in fruits, except for *RcBBX9*, which was highly expressed in flowers. These results suggest that *RcBBX* genes might play different critical roles during the growth and development of *R. chingii*.

### 3.9 Expression levels of *RcBBX* genes following exposure to ABA

**Figure 9:**
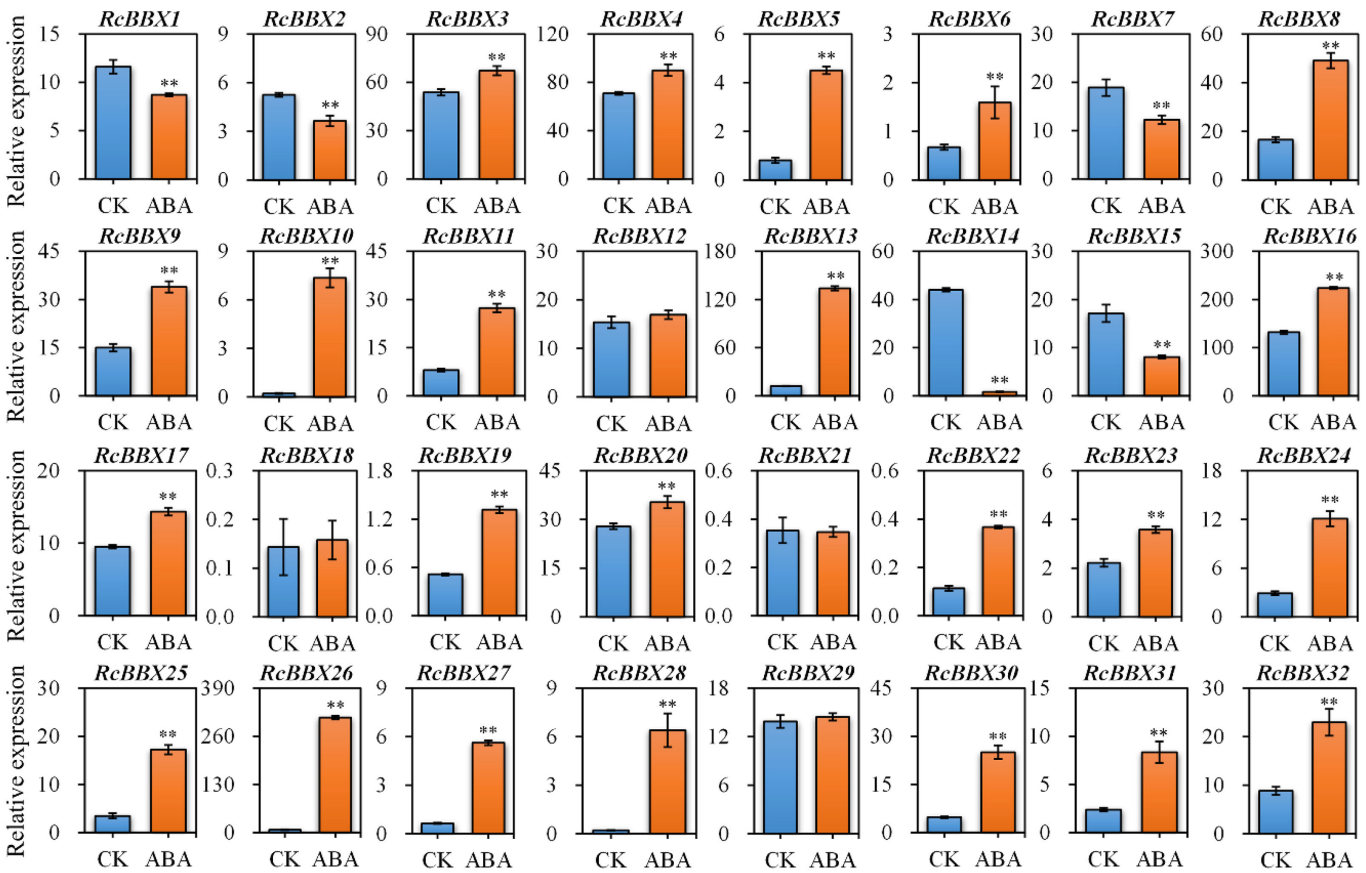
Expression profiles of 32 *RcBBX* genes after treatment with ABA in *R. chingii*. Each bar refers to the mean ± SD (standard deviation) of three independent replicates. Double asterisks indicate the significance between CK and ABA based on the student’s *t*-test at *P* < 0.01. CK, untreated group. ABA, treatment of 100 μM abscisic acid.

Since ABRE, an ABA-responsive CAE, existed widely in *RcBBX* genes (**Figure 5**), the response of *RcBBX* genes to exogenously applied 100 μM ABA was evaluated (**Figure 9**). A total of 23 *RcBBX* genes were upregulated, five genes were downregulated, while four genes showed no significant change in expression. Among them, *RcBBX26* expression increased the most (39.82-fold), followed by *RcBBX10* and *RcBBX28* expression, suggesting that ABA might be one of the most influential factors affecting the growth and development of *R. chingii* and its ability to accumulate secondary metabolites.

### 3.10 Expression levels of 32 *RcBBX* genes during the accumulation of anthocyanin

**Figure 10:**
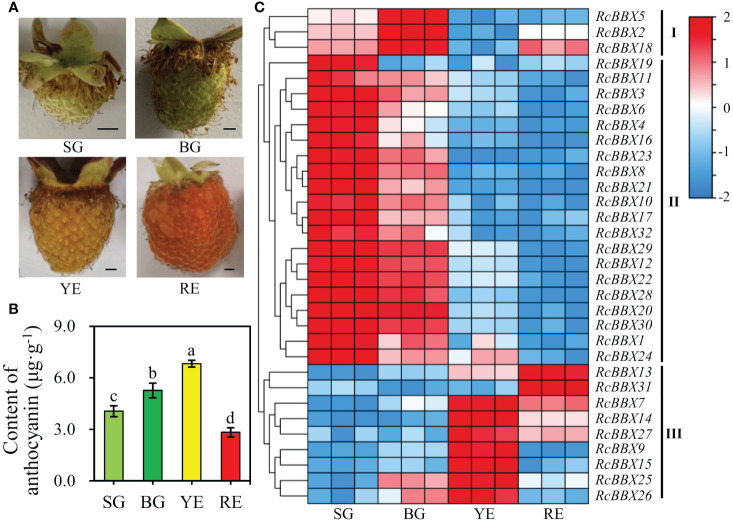
Expression profiles of 32 *RcBBX* genes in *R. chingii* during fruit ripening. **(A)** Phenotype of *R. chingii* fruits. **(B)** Anthocyanin content at four different stages of *R. chingii* fruits. Different lowercase letters indicate significant differences among different fruit ripening stages based on Duncan’s multiple range test at *P* < 0.01. **(C)** Expression of *RcBBX* genes at four stages of *R. chingii* fruits: SG, small green fruits (5-6 mm in diameter); BG, big green fruits (11-13 mm in diameter); YE, yellow fruits (14-15 mm in diameter); RE, red fruits (18-20 mm in diameter). Bar = 2 mm.

*R. chingii* fruits pass through four developmental stages: small green fruits (SG), big green fruits (BG), yellow fruits (YE), and red fruits (RE). As shown in **Figure 10**, the differential anthocyanin accumulation and expression levels of *RcBBX* genes was observed among SG, BG, YE, and RE, and was divided into three groups (I, II, and III). Most *RcBBX* genes (23/32) were highly expressed in SG or BG, and three genes were highly expressed in BG. The *RcBBX* genes displayed different expression levels at all four stages of fruit development. Two genes (*RcBBX13* and *RcBBX31*) had the highest expression in RE. Highest expression of seven genes (*RcBBX7*, *RcBBX9*, *RcBBX14*, *RcBBX15*, *RcBBX25*, *RcBBX26*, *RcBBX27*) was observed in YE. The pH differential approach was used to determine total anthocyanin content in SG, BG, YE, and RE, with YE having the highest content, 6.83 μg g^-1^, 1.68-fold more than SG (4.05 μg g^-1^), and 2.42-fold more than RE (2.82 μg g^-1^) (**Figure 10B**). This result was in agreement with the results of another report, which indicated that total anthocyanin content first increased, then decreased during the ripening of fruits (Lei et al., 2023).

**Figure 11:**
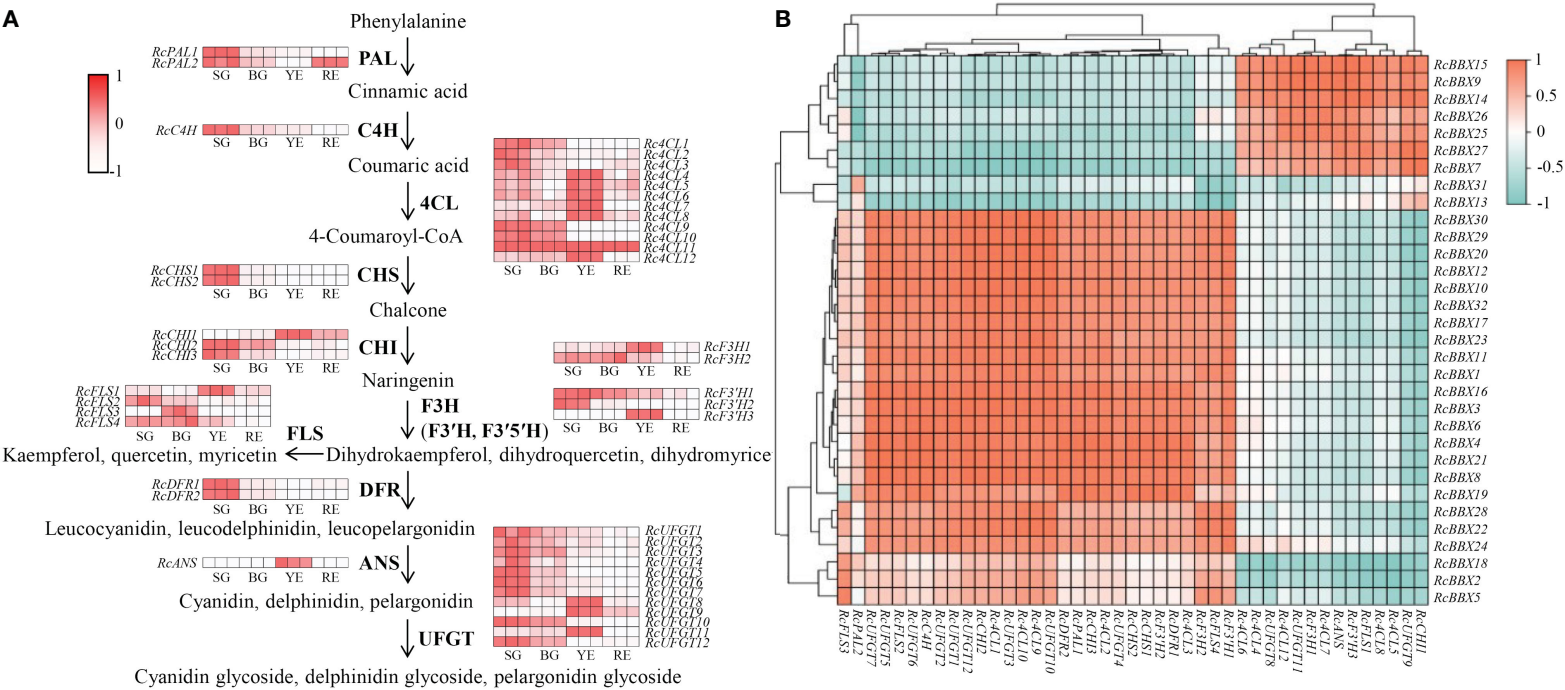
Expression profiles of enzyme-encoding genes involved in anthocyanidin biosynthesis during the fruit ripening stage, and their correlation with 32 *RcBBX* genes. **(A)** Expression profiles of enzyme-encoding genes associated with anthocyanidin biosynthesis during different fruit ripening stages (SG, small green fruits; BG, big green fruits; YE, yellow fruits; and RE, red fruits). PAL, phenylalanine ammonia lyase; C4H, cinnamate 4-hydroxylase; 4CL, 4-coumarate CoA ligase; CHS, chalcone synthase; CHI, chalcone isomerase; F3H, flavanone 3-hydroxylase; F3′H, flavonoid 3′-hydroxylase; F3′5′H, flavonoid 3′5′-hydroxylase; FLS, flavonol synthase; DFR, dihydroflavonol 4-reductase; ANS, anthocyanidin synthase; UFGT, UDP-glucose flavonoid 3-*O*-glucosyl transferase. **(B)** Correlation analysis of the expression between *RcBBX* genes and anthocyanidin biosynthetic genes.

Furthermore, putative anthocyanin biosynthetic enzymes, including phenylalanine ammonia lyase (PAL), cinnamate 4-hydroxylase (C4H), 4-coumarate CoA ligase (4CL), chalcone synthase (CHS), chalcone isomerase (CHI), flavanone 3-hydroxylase (F3H), flavonoid 3′-hydroxylase (F3′H), flavonoid 3′5′-hydroxylase (F3′5′H), flavonol synthase (FLS), dihydroflavonol 4-reductase (DFR), anthocyanidin synthase (ANS), and UDP-glucose flavonoid 3-*O*-glucosyl transferase (UFGT), were identified in *R. chingii* (**Figure 11**; **Supplementary Table 5**) based on the reported genome or transcriptome data (Li et al., 2021; Wang et al., 2021). The expression of 44 enzyme-coding genes at all four stages (SG, BG, YE, and RE) was detected (**Figure 11**). Among them, 14 genes (*Rc4CL4*, *Rc4CL5*, *Rc4CL6*, *Rc4CL7*, *Rc4CL8*, *Rc4CL12*, *RcCHI1*, *RcF3H1*, *RcFLS1*, *RcF3′H3*, *RcANS*, *RcUFGT8*, *RcUFGT9*, and *RcUFGT11*) were highly expressed in YE, which was positively related with the seven *RcBBX* genes that were also highly expressed in YE. Combined with the tissue-specific expression of *RcBBX* genes in fruits (**Figure 8**), *RcBBX26* was potentially related to anthocyanin accumulation in *R. chingii* fruits.

### 3.11 Co-expression network of *RcBBX* genes with anthocyanin biosynthetic genes

**Figure 12:**
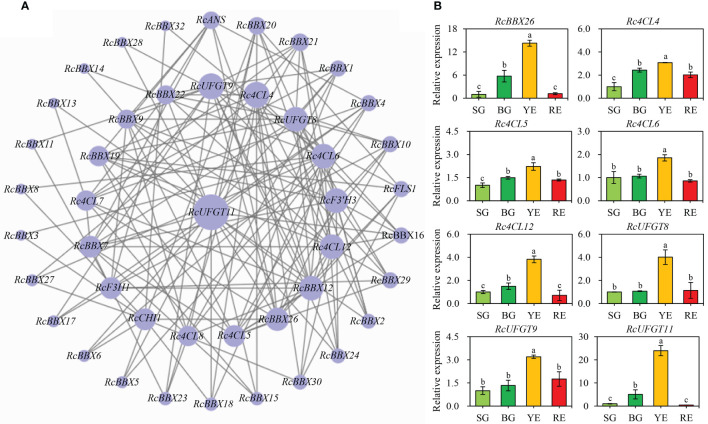
Co-expression network between *RcBBX* genes with anthocyanin biosynthetic genes. **(A)** Co-expression map using Cytoscape. **(B)** qRT-PCR analysis of RcBBX26 and its potential target genes (Pearson correlation coefficient *r* > 0.7, *P* < 0.05) during fruit ripening. The four stages of *R. chingii* fruits are: SG, small green fruits; BG, big green fruits; YE, yellow fruits; RE, red fruits. Different lowercase letters indicate significant differences during fruit ripening based on Duncan’s multiple range test at *P* < 0.01.

Expression levels of *RcBBX* genes and multiple anthocyanin biosynthetic genes at four developmental stages were matched to construct co-expression networks (**Figure 12A**). A consistent trend was observed between *RcBBX26* and seven anthocyanin biosynthetic genes (Pearson’s *r* > 0.7, *P* < 0.05), *Rc4CL4*, *Rc4CL5*, *Rc4CL6*, *Rc4CL12*, *RcUFGT8*, *RcUFGT9*, and *RcUFGT11* (**Figure 12B**), with a correlation coefficient of 0.55 to 0.92 (**Supplementary Table**-**6**), indicating that *Rc4CL* and *RcUFGT* might be the potential target genes of *RcBBX26*.

### 3.12 Cloning, subcellular localization and functional analysis of RcBBX26

**Figure 13:**
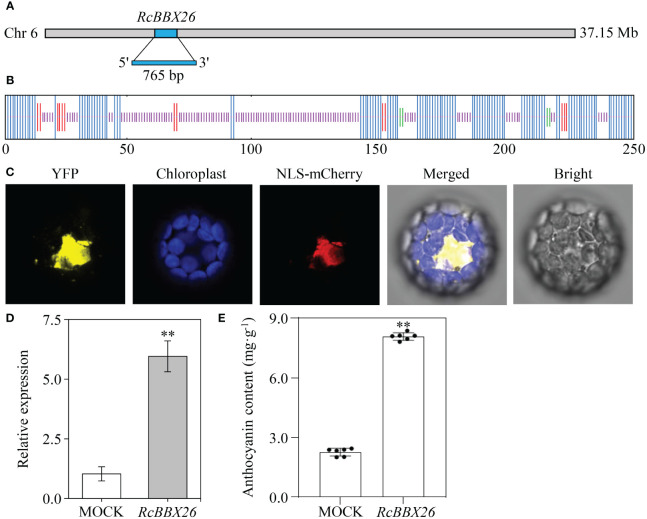
Functional analysis of *RcBBX26* in *R. chingii* leaves. **(A)** *RcBBX26* in the *R. chingii* chromosome. **(B)** Secondary structure of RcBBX26. Alpha helixes, extended strands, beta turns, and random coils are represented in blue, red, cyan, and purple, respectively. **(C)** Subcellular localization of RcBBX26. **(D)** qRT-PCR analysis of *RcBBX26* in transgenic *R. chingii* leaves. **(E)** Content of anthocyanin in transgenic leaves. Data denote the mean ± SD (standard deviation) of six independent replicates. Double asterisks indicate significance between MOCK and *RcBBX26* based on a student’s *t*-test at *P* < 0.01.

RcBBX26 was amplified, sequenced and submitted to NCBI under accession no. PP723082. RcBBX26 harbored a coding sequence of 765 bp, encoding 254 amino acids with a molecular weight of 27.40 kDa (**Figure 13A**). The two-dimensional structure showed that RcBBX26 consisted of 50.00% random coils, 43.31% alpha helices, 5.12% extended strands and 1.57% beta turns (**Figure 13B**). Besides, RcBBX26 was a nuclear-localized protein (**Figure 13C**), supporting the conclusion that RcBBX26 functions as a TF.

*R. chingii* is a medicinal xylophyte whose genetic transformation is difficult to achieve (Wang et al., 2021). Herein, efficient transient overexpression was utilized to confirm the function of RcBBX26, resulting in positive transgenic lines through semi-qRT-PCR (**Supplementary Figure 1**) and qRT-PCR (**Figures 13D, E**). Overexpression of RcBBX26 accelerated anthocyanin production in *R. chingii* leaves, representing a 3.57-fold increase compared with the untreated MOCK (**Figure 13F**). Besides, nine anthocyanin biosynthetic genes (*Rc4CL4*, *Rc4CL5*, *Rc4CL6*, *Rc4CL7*, *Rc4CL12*, *RcF3′H3*, *RcANS*, *RcUFGT8*, *RcUFGT9*, and *RcUFGT11*) were upregulated by 1.43- to 13.80-fold, with *RcUFGT11* showing the largest increase (**Supplementary Figure 2**).

## 4 Discussion

The BBX family, which belongs to the zinc finger TF superfamily, is considered to play an important role in plant growth and development. The BBX family has not only been widely studied in model plants *A. thaliana*, *O. sativa*, and *S. lycopersicum*, but also in economic plants *Fagopyrum tataricum* (Zhao et al., 2021), *Lycium barbarum* (Yin et al., 2022), *Phyllostachys edulis* (Ma et al., 2021), and *Castanea mollissima* (Yu et al., 2024). However, no information is available on the BBX family in *R. chingii*, which is an edible and medicinal dual-purpose herb.

In this study, a total of 32 genes were identified and named *RcBBX1-32* on the basis of their chromosomal positions (**Table 1**; **Supplementary Table 2**). This number is the same as the amount of *BBX* genes in *A. thaliana* (32) (Khanna et al., 2009) and *S. miltiorrhiza* (32) (Li et al., 2023), but more than in *C. mollissima* (18) (Yu et al., 2024), *Ananas comosus* (19) (Ouyang et al., 2022), *F. tataricum* (28) (Zhao et al., 2021), *L. barbarum* (29) (Yin et al., 2022) and *O. sativa* (30) (Huang et al., 2012), suggesting the diversification of BBX members among different plant species.

Three conserved domains (B-box1, B-box2 and CCT) of the 32 RcBBX proteins displayed high similarity, inferring that the RcBBX sequences have been strongly conserved throughout evolution. A phylogenetic tree categorized the 32 *RcBBX* family genes into five groups (**Figure 1**), and the RcBBX members within the same group harbored a strictly consistent combination of conservative domains, which was in line with the *BBX* genes of *A. thaliana* (Khanna et al., 2009) and *O. sativa* (Huang et al., 2012). The number of different groups varied among species. For instance, 13, 4, 8 and 7 A*. thaliana BBX* genes were clustered into groups I/II, III, IV and V, respectively, compared to 9, 2, 7, and 14 in *R. chingii* (**Supplementary Table 4**), and 10, 3, 7, and 16 in *S. miltiorrhiza* (Li et al., 2023). These findings indicate that the BBX family might share a common ancestor among different plant species, although their evolutionary changes occurred independently. Furthermore, the number of group V *RcBBX* genes, only harboring a single B-box 1, was significantly higher than in any of the other groups. The loss of B-box 2 and CCT domains in group V may be due to a highly frequent gene expansion (Gangappa and Botto, 2014), while critically functional genes might also be hidden in this group.

Six duplication sets covering 32 *RcBBX* genes were phylogenetically distributed in a discrete group with similar domains (**Figure 4B**), motif compositions (**Figure 4C**) and gene structure (**Figure 4D**), suggesting that each pair of duplicated genes probably underwent the closest evolutionary processes and shared similar roles in *R. chingii*. Furthermore, *RcBBX* genes exhibited 4-fold more homologous gene pairs with *A. thaliana* than with *O. sativa* (**Figure 3**), possibly indicating that different gene duplication events occurred during the evolution of monocotyledonous and dicotyledonous plants. The differences between groups may be associated with widespread diversity of the BBX family (Shan et al., 2022).

Different CAEs present in the promoter region play an essential role in functional diversity (Wittkopp and Kalay, 2011). A total of 836 CAEs were identified, and 48.44% of them were involved in light responsiveness (**Figure 5**). Notably, the G-box, which was mainly distributed in the promoter region of *RcBBX* genes (28/32), is an important binding site for BBX regulators, including HY5, PIF3, and PIF8 (Job and Datta, 2021). In *A. thaliana*, HY5 binds to the G-box at the promoter region of BBX11, positively activates its expression, and ultimately affects light-mediated growth and development (Zhao et al., 2020). Through GO and KEGG enrichment annotation, most *RcBBX* members were enriched in response to light or abiotic stress stimuli (**Figure 7**). ABRE, a well-studied CAEs associated with ABA-induced expression, was widely distributed in the promoter region of *RcBBX* genes (28/32), indicating that they might be of great importance to cope with various environmental stresses. It is widely known that ABA can promote the product of specialized metabolites in multiple medicinal plants, such as tanshinone and salvianolic acid in *S. miltiorrhiza*, artemisinin in *Artemisia annua*, and ginsenoside in *Panax ginseng* (Zheng et al., 2023). Herein, most *RcBBX* genes were up or down regulated (87.50%) after treatment with ABA (**Figure 9**), suggesting that they might be involved in ABA-induced anthocyanin biosynthesis.

*R. chingii* is employed as both food and medicine, and the fruit is the primary tissue. Tissue-specific expression analyses showed that nine *RcBBX* genes (*RcBBX1*, *RcBBX5*, *RcBBX10*, *RcBBX12*, *RcBBX17*, *RcBBX20*, *RcBBX23*, *RcBBX26*, and *RcBBX31*) were dominantly expressed in fruit (**Figure 8**), suggesting that they might be related to the accumulation of anthocyanin. Total anthocyanin content gradually increased as ripening proceeded, attained a maximum at the YE stage, then decreased (**Figure 10B**). Besides, seven *RcBBX* genes (*RcBBX7*, *RcBBX9*, *RcBBX14*, *RcBBX15*, *RcBBX25*, *RcBBX26*, and *RcBBX27*) initially increased, then decreased (**Figure 10C**), a trend that was consistent with the dynamic accumulation of anthocyanin. *RcBBX26*, which showed fruit-specific expression, is a candidate gene to explain anthocyanin accumulation in *R. chingii*. Overexpression of *PpBBX16* in *P. pyrifolia* callus promoted red coloration, and resulted in the activated expression of key TF PpMYB10 and structural genes, for instance *PpCHS*, *PpCHI*, *PpUFGT*, and *PpDFR* (Bai et al., 2019a). Additionally, PpBBX16 interacted with PpHY5, thereby stimulating the expression of *PpMYB10*, while PpBBX21 interacted with PpHY5, but suppressed anthocyanin biosynthesis (Bai et al., 2019b). During fruit ripening, seven enzyme-coding genes (*Rc4CL4*, *Rc4CL5*, *Rc4CL6*, *Rc4CL12*, *RcUFGT8*, *RcUFGT9*, and *RcUFGT11*) related to anthocyanin biosynthesis (**Figure 12**) also exhibited almost the same trend as *RcBBX26* expression and anthocyanin accumulation. The nuclear-located RcBBX26 was conductive to anthocyanin production in transgenic *R. chingii* leaves (**Figure 13**). These results suggest that RcBBX26 is a positive regulator that potentially activates the expression of anthocyanin biosynthetic genes (**Supplementary Table**-**2**) and thus promotes the accumulation of anthocyanin in *R. chingii* fruits.

## 5 Conclusion

In this study, a total of 32 *BBX* genes were identified from the high-quality genome of *R. chingii*. The complete series of *RcBBX* genes was analyzed, including a phylogenetic analysis, an assessment of their structure and motifs, prediction of chromosome location, and analysis of CAEs in the gene promoter region. Expression profiles of the 32 *RcBBX* genes in different tissues, at different developmental stages, and following treatment with ABA were diverse. A combination of the co-expression of *RcBBX* genes and functional overexpression unveiled the role of *RcBBX26*, which was closely involved in anthocyanin biosynthesis in *R. chingii* fruits. This study lays a foundation for further studies of these *RcBBX* genes and contributes to the ability of breeders to genetically improve the quality of *R. chingii* varieties.

## Data availability statement

The datasets presented in this study can be found in online repositories. The names of the repository/repositories and accession number(s) can be found in the article/**Supplementary Material**.

## Author contributions

ZX: Data curation, Formal analysis, Investigation, Writing – original draft. GZ: Data curation, Formal analysis, Funding acquisition, Writing – original draft, Writing – review & editing. JC: Data curation, Formal analysis, Investigation, Writing – original draft. YY: Data curation, Formal analysis, Investigation, Writing – original draft. LY: Data curation, Formal analysis, Investigation, Writing – original draft. XL: Data curation, Formal analysis, Investigation, Writing – original draft. JT: Investigation, Supervision, Writing – original draft, Writing – review & editing. ZY: Conceptualization, Funding acquisition, Project administration, Supervision, Visualization, Writing – original draft, Writing – review & editing.
