# Supplementary material for: Role of Rubus chingii BBX gene family in anthocyanin accumulation during fruit ripening
Source: Front Plant Sci. 2024 Aug 2;15:1427359. doi: 10.3389/fpls.2024.1427359 (PMC11327127; doi:10.3389/fpls.2024.1427359)
Supplement: Supplementary file 1 [file DataSheet_1.pdf]

Supplementary Material

Table S1 Specific primer of 32 RcBBX members used in this study.

Table S2 Protein sequences of 32 RcBBX proteins.

Table S3 BBX protein sequences used to construct the phylogenetic tree.

Table S4 Domain organizations of BBX proteins in *R. chingii*.

Table S5 The 44 anthocyanin biosynthetic enzymes in *R. chingii*.

Table S6 Correlation between RcBBX26 and anthocyanin biosynthetic genes.

Figure S1 Semi-qRT-PCR analysis of MOCK and *RcBBX26* in transgenic *R. chingii* leaves.

Figure S2 Analysis the transcript level of anthocyanin biosynthetic genes in transgenic *R. chingii* leaves. Asterisks indicate significance between MOCK and *RcBBX26* based on the student's *t*-test at $p < 0.01$.

Table S1

Gene ID	Forward primer (5′ to 3′)	Reverse primer (5′ to 3′)
RcBBX1	CGCAAGTGCTTCCTCAAATG	GCTCCGCATCTATATCGTAAGG
RcBBX2	GATCGACGGTATGGGAAGAAAG	TAAACGACCAGCACTGTAACC
RcBBX3	AGGTGAGACCAGAAGAGTACAA	CATCATCACTGGCATCCGATAG
RcBBX4	GTCGGCAGTATGGTGATTCTAA	TCATCAGATCGCCTACCATTTC
RcBBX5	TACCTTCCCATCGTCTCCATAG	GAACACCACTTTGGCGTTATTG
RcBBX6	TACCAAGCACCCAACAACTC	CTTGGGTGTAACGGGTCTAATG
RcBBX7	GAAGCAGCGTGTCTGTGATTA	GACTTCTCGGTCGCAAGTAAAG
RcBBX8	ACGAGCACTGCTTCGAAAT	TCCAGTGACGATGAGGATACA
RcBBX9	TCAGCAGGACAGAGCAATTC	CAGAGGTAGCAGAGAGCTTAAC
RcBBX10	CCGAGTTCACCATGCCAATA	CATGCCTTTCCTGGCAGATA
RcBBX11	CGCAGGAAGAACGAGAAGAA	AACCTGAAGTAGAGGGTGAGA
RcBBX12	CAGAGCTTTGGCTACATGGATA	TGCACTCATAATCCTCCTGTTC
RcBBX13	GGAAGACGATGAGGATGAGAAC	CGCTTCACTATCCGAAGAACA
RcBBX14	CCACCAGGCTCAACTTCTTTA	CCTGCGCTTACATGGATATGA
RcBBX15	TCCACAGCCTTCTTCTCTATCT	GCCTCGATGATCTGCAGTAAA
RcBBX16	CTCCGAACGGTAGGAACAAA	CTTGCCTTCCTCGACTCATATC
RcBBX17	GGCATTGACCTCCACTTGTA	GGCATGTGATGTTGGCATTT
RcBBX18	GTGCCAAATGCGATGTTGAG	GTCGCATCTAGGGAGTTTGTT
RcBBX19	GTTCTATCTGCCACAGGAGTTT	CTGCTCCCATTTCCTCGTTAT
RcBBX20	TCAGCCGAGTCAATCGTTAAG	TGAGGTTCGGAATGAGGAAAG

RcBBX21	GCGGGTTTGTTACCGAATTAC	CATCAAGCCCTCTGCCTAATA
RcBBX22	CAACCCTGTCATTCTCTGTCTC	CCGAGTGTCCAACATCCATT
RcBBX23	CCGTGTGACGAGTTGGATAA	CAGTCCTCATCAAGTCCCTTAC
RcBBX24	GCAACTCACAACCAGCATTAG	GTGTTTGCCTCTTGTGTGATG
RcBBX25	CCAGGAGACTGTTGGTTACTTC	ACGAGTTCGCTGTGTGTATG
RcBBX26	CTGTGTCTCTAGCACCGAATC	AGCATCCCAAGCCTTTCC
RcBBX27	CAACAACAAGGAGGGAGGAA	CTTGTAGTTGAAGGAGGTGGAG
RcBBX28	GCCAAGTGTGCAACTCAAAG	AGCATTGGCCGAGTGAAT
RcBBX29	CTGCTGAATCCCGTGAAGAA	ACCTCCACTCCGAAGAAGAA
RcBBX30	GTCAACAATGGCGAGAGAGA	AAATCGTCCTGAGAGCGAAG
RcBBX31	GAACCTTGGGACTCTACTTTGG	CTGGCCTTTCTAGAGGCATATC
RcBBX32	AAGCCTGCAAGACCCTTATC	CAGCACACCGTCGTCTAAA

Table S2

Protein name	Amino acid sequences
RcBBX1	MGSRKEEERNEKIIRGLMKLPPNRRCINCNSLGPQYVCPNFWTFVCTTC SGIHREFTHRVKSVSMSKFTSQEVEALQNGGNQRAREIYLKDWDLQRQ RLPDSSKVDKIREFIRSVYVDRQYAGGRTSEKPPRDLQNHRIREDETRR ASSYHSYSQSPPYDYQYEDRRYGKKAAALTRKPGSDRGRYEGKLSSFG YSAGRLSDQMYEDRFENDGSGSIVSDYSVSSGGDASRSGVQSPNFQKD IGSSSPSFQPSQDTISEVRCQAKSTVLETNVNRDAEAKPFSQRTVSSGSF GSVDSYSMSVKSFNSGGLTDAVSEPDQYAGTLQAKPTSLSSLFGSSDRL DLFKAPVQQEISSAASSVDLFQLPAASSSLSIHSFQPSVLSSASSLNVHQP PQALTPSLELFADFPQHQSPAKQVQESVPKNEGWATFDTHQPSTSISGT ENIIPANIASNDGGSIGNFDLFSSSNPGMQWPSFENISNPWSDNLHDFTA PNTTESTQSWNAFEDSIGHFSLEGTKKSGESVGTDKLLSAGDQYMGVR ISEDSSKDGIQRAASEGEPHDPSLPLHILGPPYTPQVLPQMGEIKSDATN HRSNNPFDIPYDIDAEQTNMFFDISSLQASLPNAPLQSSFLSDISQPWFPQ NAATVMPYIPAAVEGGLTYLAGQAQSTQIPNVSTQGPVASIGGNPFA
RcBBX2	MGSRKEEERNEKIIRGLMKLPPNRRCINCNSLGPQYVCPNFWTFVCTTC SGIHEFTHRVKSVSMSKFTSQEVEALQNGGNQRAREIYLKDWDLQRQR LPDSSKVDKIREFIRSVYVDRQYAGGRTSEKPPRDLQNHRIREDETRRA SSYHSYSQSPPYDYQYEDRRYGKKAAALTRKPGSDRGRYEGKLSSFGY SAGRLSDQMYEDRFENDGSGSIVSDYSVSSGGDASRSGVQSPNFQKNI GSSSPSFQPSQDTISEVRCQAKSTVLETNVNRDAEAKPFSQRTVSSGSFG SVDSYSMSVKSFNSGGLTDAVSEPDQYAGTLQAKPTSLSSLFGSSDRLD LFKAPVQQEISSAASSVDLFQLPAASSSLSIHSFQPSVLSSASSLNVHQPP QALTPSLELFADFPQHQSPAKQVQESVPKNEGWATFDTHQPSTSISGTE NIIPANIASNDGGSIGNFDLFSSSNPGMQWPSFENISNPWSDNLHDFTAP NTTESTQSWNAFEDSIGHFSLEGTKKSSELVGTDKLLSAGDQYMGVRIS EDSSKDGIQRAASEGEPHDPSLPLHILGPPYIPQVLPQMGEIKSDATNHR SNNPFDIPYDIDAEQTNMFLDISSLQASLPNAPLQSSFLSDISQPWFPQN AATVMPYIPAAVEGGLTYLAGQAQSTQIPNVSTQGPVASIGGNPFA
RcBBX3	MRTLCDSCESAAAIVFCAADEAALCRACDEKVHLCNKLASRHVRVGL ATPSAVPRCDICENAPAFFYCEIDGSSLCLQCDMVVHVGGKRTHGRYL VLRQRVEFPGDKPGNIVEDPPSQPIDPGETRRVQHQQPRMTIGENHQNH RTSPIRLSDASDDGHVKMDNKLIDLNMKPNRMHGQASNKEDQ
RcBBX4	MNLTRTATVRELLHLQRIEEVRMANRVKEDEKNERIIRGLLKLPENRR CINCNSLGPQYVCTNFWTFVCTNCSGIHREFTHRVKSVSMAKFTSQEV KSLQEGGNQRAKELYLKELDPQRNSFPDSSNVERLRDFIKHVYVDRRY TGERNFDKPPRVKMGDKEDSYENRRLDYHGGSRSPPYDDERRYSERSS PGGRSYEEQRSPGYDQESRQYGDSKRSPSRPEIVNDWRREDRFGNGRR SDDRRISDGDSKLDAKSPERPRDINSSSPPMVRPVRDILGEKTIPLRIIEPP KANSVRATDGSVVTQRTASSSSLGSANGNPVEVKLETSGSLIDFDADIE PASAAAVPQAQQTSVTQSYSQPVNSDSDTNWASFDVAPQVKVSQAPA NANSLESLLSQLSVSAPVPSYVSGTAGNTGAFTAAGQMTAPFSGNSVIT PVGHTMLPTATGSHTIAPVTSLSTFPPVGAPVAAPGLAQIFPANAGNFP ATGSGQWPNMPHQQPSLFPSNGLQSASQQYIPSVGGASSNQPWNLAH APNAQVQPSNPATQAPQHVSRSIHNVSTIASQPSAGDVKSIGRSELPAD

	LFAMNYSSFPTPVPGWQTGPPHGMGFAMPYNTAVPMPTFQQSLKSAN PFDVNSEPPPVQATTFPPVASSQGALPNVRPPSGLVRTSSLGAPSSAWM PLQSSSYSSGLPLQAPPYASQMPPSAYMGQQIPSSMPPSGYQGVGAFGA EGAAFGSLNMDQQVIGRFSAPATPNPFPSAGGNPFG

RcBBX5 MGIQKPAWLEALYTQKFFAACSFHETAKKNEKNVYCLDCCTSICPHCL
PSHRLHRLLQIRRYVYHDVVRLEDLQKLVDCNNVQSYTINNAKVVFIK
KRPQNRQFKGSGNYCTSCDRSLQEPYIHCSLGCKVDYVLTLKRGLSPY
LKKCDSLQLSPDFLVPLQSESDDQDMTSDQTLHSTIVDCDDPMSYSSSS
GSENMSMAYTNGDQIVRKKRSGLNYSYVYSARSARKVSDDDQDMAS
SIISRRKGIPHRSPLC

RcBBX6 MGNRIKEEEKIERIIRGLLKLPENKRCMNCNSMGPQYVCTTFLTFVCTN
CSGVHREFTHRVKSVSMAKFTAEEVSSLQAGGNERARQIYFKEFDPQY
HSFPDSSNINRLRDFIKHVYVDRKYTGERSVQKLPRLRLSEEPDESRKV
GAYHGGSRSFHDEERKVGAYYGGSRSFRDEDWKVAVPYGGSRSFRDH
EDRFEQNYSQGSSPSVRDQNKKSSILSRKFKKRRFQEKPLRFEVVDNRI
RDDRKARSGFPTGESRSQSRTPENKKNMDSSSFPVARFVKGKPGENEL
PPQVGEISIANDQKHADGSAHNQKMKSMSSHDGNAVELKSQPQQIPPL
NNDSNWASFESSTKENASQVPKPDTLESLLFELSTPTSVPAITASEAPSN
DDAPSTACTNNMSAGGVASDAPAEQMLSLFDTVCASTSSSISTSVPVQP
SDAGHLQALPTSGGDTSVRVTDAQQLPSTQQDQYSISFASESGFTSQHT
VTPVGASNGLSRTSSLAPNTQGSLSVSAETPSQSMLRTQSLPVETKSSE
REELPVDLFAASYSSIPSQASGWHNGPPHGMGFHMQYYPNIEPIDILVS
FTACSCISGSRKPTNPFDLNDNKSVVPSTHFPSMSSVEDALHNVSVPPSL
MHTSSLGSYSSHLKPPQSPSHESMMPSHSLPSYASFSPSMGEQLHNNAQ
FLRPHEIGGFSSNEAAVFGNLSTSQQPSIRYQAPNNSETFSPMGERGTTL
YTNCIRPVTPKINIINPVVTPAAIISGIPKSGPFVNAEATNGLHGLDGHW
DAVYGTTNNVVVPDENQCCTHVKVLS

RcBBX7 MDGSKEGGGGGGHQNMRAQKQRVCDYCGDSMALLYCRADSAKLCF
TCDREVHSANQLFSKHTRSQLCDACDKAPASIFCSTESSVMCQNCDWE
RHNLSSSSVHDRRPLEGFTGNPCLNELLAFVGFENLDKKALIFSEESGS
GGDGDGLDGSLGLSDGFSDLLVWETPSVVSLDDLIVSNPAHKFQAMG
VPPLPKNRNAACGRHKEEVLSQLRTLAKSEPDLMNENVDLNSFMSFQS
LESEQNMQSAGFCTIFEQDAEPLAFPAYEAQEFESNDCGKSANQDFFPK
TLQRSYLQDCSMVPDLNSNNDGTASHASDGYGGQMNSEASSAFPKVP
SHECSLHRESALSRYKEKKKTRRYDKHIRYESRKVRAESRTRIKGRFAK
MNN

RcBBX8 MASKLCDSCKSATATLFCRADSAFLCINCDTKIHAANKLASRHARVWL
CEVCEQAPAHVKCKADDATLCVTCDREIHSANPLSRRHERVLVTPFYD
SLNSDNSSPVKSGAAVNFLDDRYFSDVDGETTEVSREEAEAASWLLPN
PKAMDSPDLNSCQYVFSDMDPYLDLDYGAVDPKIEAQEQNSCGTDGV
VPVQSKSVQPQIVNEHCFEMDLPGSKPFIYGFNGHCLSQSVSSSSLDVS
VVPDGNMLTDVSDPYPKSIGSMVDQLSHPTVQISSADREARVLRYREK
RKNRKFEKTIRYASRKAYAETRPRIKGRFAKRTEVEIEAERLCRYGVVP
SF

RcBBX9 MKIQCDVCNKDDASVFCTADEAALCEGCDHRVHHANKLASKHQRFSL
IHPSSSKQSPLCDICQERKAFLFCQQDRAILCRECDVPIHSANEHTQKHS
RFLFTGVKLSATSAVYTSSSDSATVTDLKSQINNKSIVSAASISNPPSIPKI

SATTTTTTKNCGDLLLNDGVGATSSISEYLIETLPGWHVEDLLDFSSKPF
GFCKADDNGVLPFFDDDIESNLSSFSSENMGLWVPQAPCPSLQYSQMG
GGGLIGFKESPKDAANMNMIKANNNYNRSMWNDVDAFTVPQISPPSV
GSKRTRPF

RcBBX10 MKIRCDVCDKAEATVFCCADEAALCDVCDRRVHHANKLASKHKRFFL
LQPTVKDSPQCDICQERHGFLFCQEDRAILCRECDHSIHKANEHTKKHN
RFLLTGVKLSTDTTLLPTSSSSCSTFSDGGVINNSTDHARVTKSSTKRPR
TGCNEQTLSSSSYKVEENCSISDNGLVISTNSISEYLMGDQTAEWRVED
LLDVSFDFDGLYEVGRAPYI

RcBBX11 MVRHHQREVCSSSTCMEDEMNSMSKPAWLQGLMGETFFGGCGVHEN
RRKNEKNVFCLHCCLSICPHCLQSHRSHPLLQVRRYVYHDVVRLGDLE
KLIDCSYIQPYSNNGAKVIFLNQRPQSRPLSRTNGNKGFAANICFTCDRI
LQEPFRFCSLSCKVDHLVLQEEDLSGILYRFDESDFTISQFEGLRMDGSE
VIDDDGQMMPSSILEDHELQFRDSSCSNNSDSVMSRSTEAVVKKKNKG
ILPGIMLSLGGRRKGAPQRAPLS

RcBBX12 MKIQCNVCEAAEANVLCCADEAALCWACDEKVHAANKLASKHQRVP
LSASHMPKCDICQEAVGYFFCLEDRALLCRKCDVAIHTANSLVSGHRR
FLLTGIKVGPEPNPPGSGGGVAGSSSSVKSHSGSLSKFDVNNQLAEECK
VAPSSVDGMPFAGGSAAGSAPQWPMDEFLGFTDFDQSFGYMDNGSSK
ADCGKLGESDSSFLRSSEEEQEDYECIGQVPETSWMVPQVPSPPTASGL
CWPKSYQGPSDCAVFVPDVCYSEMQNSLHCQQNDISAGPLTSSLTI

RcBBX13 MKECELCGLRARMYCDADHASLCWDCDEKVHGANFLVAKHPRRLLC
HVCHSPTPWTGSGPKLTPTVSVCENCVQSHRKIDLNQEQESEAENEED
EEEEFDDEDDDVDDESEDEEDDEDENQVVPWSCCSYSQAQPPPAACSS
DSEAEISGSKRVRENVDVDSSDDEVGFNGTALYGGSLRPLKRPRLNED
QNHLQRSSDGDGGCEAEWRAKIIMSSMQRLEKEVIDGGDHPSAAVLRI
WKLSRDEGSR

RcBBX14 MRKCELCDSVAKMYCESDQASLCWDCDIKVHGANFLVAKHSRTLLC
HVCQSLTPWNASGPKFGPTVSVCLNCVNTSNKESRNEEEEEEEGDDSG
GDDDDDDDEEENSIREDDDHEEENSIGEDDDHDGGGDNDADDNGGG
GNDDDDDEENQVVPWSSSFSPPPGSTSLSDEEECCNESFSKSRSSYSYP
CKRRRFNAH

RcBBX15 MEEEMLVPSWLESLLSTAFFSICPTHRDAPRSECNMYCLDCHNGAFCF
YCRSSRHRDHQVIQIRRSSYHDVVRVNEIQKVLDIGGVQTYVINSARVL
FLNERPQPKTAVKGSSHICEICGRSLLDPFRFCSLGCKLVGLKKNGDAS
FIFSAKNGEDNEEGRREGLAIRLPSKEVDHQDPLRSEGTMQPKQEQDM
YQSTPPPPPPQYSRSRRRKGIPHRAPLGQ

RcBBX16 MVSPKSGTGEGIPCDFCSEQPAVLYCKADSAKLCLFCDQHVHGANLLS
RKHVRSQICDNCASEPVAFRCSTDNLVLCHECDWDSHGSLLRLRRPRS
HPDRGLLRLPVASRARLLMGTRSPRHQQESGSGRPDPEPEQGLGLLLLL
LDRNGGVYQNCGELIPAKRQSSGGILGKQKHGIQKQLVELLRRDFDGG
GENLEAPIPNSNGWQRSDDAHANANANAEGFDVENGRVDGVNVGGA
TVTSQPLLQEQAPFTSLLMMAEGIVDRQLLWDTNPHTHTTHNTQIWDF
NLGKLRDHEETVPLDVAYGSSASGFMIKNFSELLKEASVTDTKMFGDV

YQMNCPIGQDDITFNNNSNNPSASQGPATSESNNFTVGRPSSGSAFGED
NGSGASKDTHIMEQPFLIRSDSLRTVGTKADMELLAQNRGNAMLRYK
EKKKNRRYDKHIRYESRKARADTRKRVKGRFVKATEAPDGDILIFWK
NNLVDPNNVFQSWDATLINPCTWFHVTCNNEDSVTRLDLGNAGLAGP
LVPELGKLTNLQYLELFHNNFSGSIPREIGQLVHLVSLDLYKNKLSGPIP
ETFGHINSLRFLRVFRNNLTGSIPSSLGKLTNLQILKLNSNKLTGAVPVK
VIELVRFGKLAILDVSNNLLAGTVHRTNTTVVIASVDCNSEGDILYSWK
TKLVDPNNVLASWNQTSTNPCPWFHVTCNSENIVTRLDLGNAGLSGPL
VPELANLTNLQYLELYKNKFYGSIPWEIGHLKELISLDLYQNQLSGSIPE
SLGHLNSLRFLRVFGNNITGAIPSSLGNLTACVILKLNSNNLSGVLPVEV
IELVRFGNLTVLDVSNNLLEGTIRRTNSTGFAVTKIIQDPKVKN

RcBBX17 MKIQCDVCEKAPATVICCADEAALCAKCDVEVHAANKLASKHQRLLL
ESLSNKLPRCDICQDKAAFIFCVEDRALFCQDCDESIHLANSLSANHQR
FLATGIRVALTSTCTKDAETSSLEPPVHSSQQVSTKMPTSHASGFSSPW
GVDDLLQLSDFESSDKKESLEFGELEWIADMGLFGEQFPQEALAAAEV
PQLPVSQSSNYMSYRPPKSNSAYKKPRIEIPEDDEEHFTVPDLGIF

RcBBX18 MKIQCDVCEKAPATVICCADEAALCAKCDVEVHAANKLASKHQRLLL
ESLSNKLPRCDICQDKAAFIFCVEDRALFCQDCDESIHLANSLSANHQR
FLATGIRVALTSTCTKDAETSSLEPPVHSSQQVSTKMPTSHASGFSSPW
GVDDLLQLSDFESSDKKESLEFGELEWIADMGLFGEQFPQEALAAAEV
PQLPVSQSSNYMSYRPPKSNSAYKKPRIEIPEDDEEHFTVPDLGIF

RcBBX19 MQQKGHVVVPQWLKIMCSTAFFRACIQHPDAKKNDLDHFCIDCLQPIC
LNCLAQHLFHKHVKIRRYVYSDVINRRDLCKLFDCSGIQTYFTNRAKV
VFLKQRHQPQQQQQQNNNNNKSREYMCSICHRSLQDNSLYCSVACKV
LAIHGNECQRKNLCAGHRFEDNNEEMGAERYREVPFTKRQKLRRKGV
PLRAPMF

RcBBX20 MGIQAGWSLPGLTPKPCDTCKTSPAAVFCRADSAYLCLPCDSKVHCAN
KLASRHQRVWMCEVCEQAPAAVTCKADAALCVTCDADIHSANPLAR
RHERVPVEPFLDSAESIVKSTSALNALVPNGDVSSIKVDDADAFLIPNLN
FSSKFVDAALDIKPGDMFFPEMESLLDFEYPNPIHNTSSGMDSVVPVQP
DPIPPPPSVLNQQPSHENCFHIDFCRNKLSSSFSYPTQSLSQSVSSSSLDV
GVVPDGSSLSDISYPFVRNVNHNGIEPGVPVSATASQATQLCGVDREA
RVMRYREKRKNRKFQKTIRYASRKAYAETRPRIKGRFAKRTETETDV
MDRFYPGLT

RcBBX21 MISNKKAAASAAAASVVGAKTARACDSCIKKRARWYCAADDAFLCQ
ACDSSVHSANQLARRHNRVLLKTASSLKSSRTSSNNNSQVPSWHGGFT
RKARTPRHGKSVTPRSRFPLVPEVGADDVSYEENDEEQLLYRVPVFDP
FVAEPCTTSPNSNTDINGNESKAGLLPNYYSDNGNSSSRGFHLPSDMDL
AEFAADVDSLLGRGLDDDECFGIQGLGLMDCNEKESTTTEDCSSGRVK
CEEDEENGYTACQAETEIDLMREPFVLNFEDYDDSPASCAEEEEDEDD
KLEVRMSLDGMKTSNEEHNNCNKRKIFLRLDYDAVITTWDSQSKGSP
WTCGDRPDFNSDCLPDWMGTSSGGELLHYPLSGVVGVHPAMTDGGR
EARVSRYREKRRTRLFSKKIRYEVRKLNAEKRPRMKGRFVKRASFAAS
GMLN

RcBBX22 MEKFCEFCKALRPVVYCKADAAHLCLSCDAKIHSANTVFNRHHRTVL
CDSCRCRPAYVQCLDHRMFMCNACDRSQHASSSQHCHKRAIRSYTGC

PTAKDFAALWGLQLNELDNKSSAHLDQTLSTSCASCGSNVVNLDICGQ
SCSHIECVPGPARFEMGSTCQQYKIYHREGEVHNKTSFILHQILDLERLQ
LTEGNSPSAQSDLSSSAHYTCKRCNENLYQHLQHSETSGTNFQQKESLI
QDLKVDPLPFPFSQPEHLPTNSTIGLPLQTESIWQCRSPVQSSQLWSQN
MQDLGVCEELVCRDDFNIPDVDLTFRNFEEIFGGDQDPIRTLLDVKDVS
YSSVERDMSLNKSDNCNSRAVDASEASSMYLNESADFIQDTGASNKV
ANVSGSMDSDSPCPIQPSSSTLSFSVSRFSTVSSIADCHDTQPSSNSPEMD
VGHSEFRANSRIRYKEKKHSRLIEKKLPYPSRKATVEVSKRGKGRCVK
TEEDVDSDTIDVTRSY

RcBBX23 MELPKAKNIGGGGGAKTWKVFNGILSKKHSSFLAQPLDRMECNTSSGS
DARCLSLAGPAVAAEANKSKEIIELPKQSTKSVFLKFSHILEFSIPTNTGA
HKIRRDQLWPCPSLAEGPHGSPIRLVTNENSVNKMKFRSLANEHYYSL
HATAHKFHQTHSVSAAQVLYMITEKKAANAMGANTARACDSCLVKR
ARWFCGADDAFLCKRCDASVHSANLLASRHERVRLNTSSYKLNHHLA
TEAAQEPPLPAWQQGVTRKARTPRGHGNHKSGKDEAGKVLEQPMPFV
PEMGSEEVDYCCLEDDHESEDLEQMLYRVPIFDPFEAELCNNMTNTDH
HEVRIGNVLEDYGARTSSPCDELDNLQGLILPSDMELAEFAADVETML
GKGLDEDCSDIKGLGLSDGTKEAEQYGMDICDIGIEERKLVKVEEEDQ
GMECEFNPSMLDWNLDYDDESAGVLLGGGISTDLEEEEKVVVLLEAE
KKRKRICLRLNYEAVITAWASQRSPWTTGVKPELNPDDGWPDCMGM
WGADQIHNLQGHGDSSAAGCVMGMRGNNEQRPGREARVSRYREKRR
TRLFSKKIRYEVRKLNAEKRPRMKGRFVKRTSFQLGMGTS

RcBBX24 MGYICDFCGDQRSMVYCRSDAACLCLSCDRNVHSANALSRRHSRTLL
CERCNSQPALVRCTEERVSLCQNCDWMGHGASTSAASHKRQTLNCYS
GCPSASELSSIWSFVLDLPSTGSGESACEQEMGLMSIAENSTGSTWSPPE
NNTRQNASDTFEVNDVGAMDKSDGLVGSSSVPALNSAPQVVGQMAG
SANSTLPKLYCHGTKSPGLCEDDDLYDDFDMDEMDLNLENYDELFGV
SLNHSEELFKNGGIDSLFGAKNMSRAQDVVAAEGSSIGRVNALQQPAC
STAASVDSVMSTKTEPIVSFVPKQAQSNLSFSGVTGESSAGDCQDCGAS
SMLLMGEPPWCPPGPESSFQSANRSNAVMRYKEKKKARKFEKRVRYA
SRKARADVRKRVKGRFIKAGEAYDYDPLNQTRTRSY

RcBBX25 MKIQCNACEVAEAKVLCCADEAALCWGCDHQIHAANKLASKHHRVP
LASSSSMPKCDICQETVGYFFCLQDRALLCRKCDVAIHTANSYVSSHH
RFLLTGVKVGIEIKDTFPTQIGGVGTLVSEKVPLNRSITTSAGTIQGWHL
EEYLGSTNPNQNYGVLDNMPSKIYGSYCV

RcBBX26 MQDRSCELCNQQATLYCASDSAFLCFHCDSRVHGANFLVARHVRQPL
CSNCKGLAGDAISGEGVPPSVRWLCSSCSPENEDTLSSFDSACSACVSS
TESLAGTTTTTKVGLQRSESSVTEVSGKAWDAPARFTKRRMQRARAPT
SADARAEGTFENWCKKLGLSRNSALVVSSASHALGFCLGRLPGVPLRV
SLAASFWFGVRFCGVSTCQNLRRVEEISGVPAKLILAVDAKLGRELRV
RRARPELEEGWAEC

RcBBX27 MDTTMLVPPWLEQLLTSSFFTICRTHGDAARSECNMFCLDCGGDAFCF
YCRSSRHKDHQVIQIRRSSYHDVVRVSEIQKVLDISGVQTYVINSARVL
FLNERPQPKAGIKGVPHICEICSRGLLDPFRFCSLGCKLVGIKRNGDANF
TLEARNEEGNGNGRREGSTTTRREEDHHQLREGSQQDMYPDTPPPPPS
TTRRRKGIPHRAPFGT

RcBBX28	MGSWCQVCNSKVAALYCKADLAKLCLLCDHDIHSANALSLRHTRSQV<br>CDNCRAQSASVVCFTHNISLCHTCDRISPCSSADRTPVQGFTGCPSAME<br>LASVLGFDLGSQAQNFNLSLDDDHNKLIKHSLGRPKDEIYEQLVEMRM<br>RALAFSAQQQQQQQVEDAASYSFISIDQARRPTPGQVLASEIHLNSNI<br>EVVTDGRRSRCNAILRYNNKKNKKKKTSRQADVIYILHFFTFSFNIYTL<br>NINYVVHAS
RcBBX29	MLKEESNDAAAGNSWPRICDTCRAAACTVYCRADSAYLCSGCDATIH<br>AANRVASRHERVWVCEACERAPAAFLCKADAASLCTACDADIHSANP<br>LARRHQRVPILPISGFHHSPPATDSGGQILVGSTLADTTEDGLDEAMDE<br>EDEDEAASWLLLNPVKNGNGNGNNHNTNTNNNPSNNNNGFFFGVEV<br>DDYLDLVEYNSSSCADQNQFSTTSATNDQHNQYAVPHKISYGGDSVV<br>PVQYGDGKVTQMQMQQQHKHNFHQLGMEYESSKAAYSYDGSITHTV<br>SVSSMDVGVVPDSTMSDMSVSHPRTPKGTIDLFNGPTIQMPTQLSPMD<br>REARVLRYREKKKTRKFEKTIRYASRKAYAETRPRIKGRFAKRTDIEVE<br>VDQMFSTSLMGETGYGIVPSF
RcBBX30	MKNCELCQLPARTYCESDQAILCWDCDFKVHGANFLVARHSRTLLCR<br>ACHAQTPWKASGEKLGHTFTVCERCVARNENRGDDEESQGGNDDDID<br>TDNDDLDEDHVSDDDDDADGDIDFDEDGENQVVPWAATPPPPAASSS<br>GSEDEASLVNNGERDDSRSVAAVFLKRARDDASDLRSQDDFHRSTAR<br>LRRQTACTGHSGRSGADGGAISIDSSKDRRVDLNGSGSRTSPAIESPRR<br>NRQQNSRKLSEGSEAVDLDSSEPRIPQI
RcBBX31	MARLCLNCDGCVHSANALARRHPRWLLCDKCNVQPAIIRCLDENVSL<br>CQSCEWNHSNGVTGMGHQSQAISCYTGCPSLSEISRIWSAVLDGGSAS<br>GGFAASGWESLAGSGVPKNDTDCISNCLERRDSEGSSFGVVSAGKLNE<br>VLAESNCTPKFEPWMAPSTIIPSNPNCIQPQCKDQAPFLPQESSQLPKEL<br>QDSSNFKDLGIQDDHDICEGLNMDDVPLDVENGDQLFSCSEGPSRYPF<br>EDGELDCLLMDQKNLSVTESNGPLSDNAIQQASPSRQQDCTVGFQSSC<br>VSDSVMAPVMNASSSVNCSLLMNPNCTRNINLQGLINPTGQVHSSISLS<br>LSSITRETTHPDYQDCGLSPVFLSAEPWDSTLETGSPRARDKAKMRYEE<br>KKKTRTFGKQIRYASRKARADTRKRVKGRFVKSGEEYDYDPLVRSSF
RcBBX32	MFVPSNLLPSWLAVLLTEKFFNACIIHEEERKNEKNIYCLDCCISFCPHC<br>FTPHQSHRLLKIRRYVYHDVIRLDDAARLIDCAFVQSYTSNSAKVVFLN<br>QRPQTKNSRGSGNMCSTCDRSLQDPYLYCSISCKIDHLIRTEGGLSKYL<br>RQCKFMALPDPGLDDGVLTPDSVLETACSVRTSSGSGGYAELGCLSLA<br>CTATTEVVRKKRSSLSALRAACRPVFSPVSEISGGRRKGTPHRAPLH

Table S3

Plant name	BBX protein sequences
Arabidopsis thaliana	>AtBBX1<br><br>MLKQESNDIGSGENNRARPCDTCRSNACTVYCHADSAYLCMSCDAQVHS<br>ANRVASRHKRVRVCESCERAPAAFLCEADDASLCTACDSEVHSANPLARR<br>HQRVPILPISGNSFSSMTTTHHQSEKTMTDPEKRLVVDQEEGEEGDKDAKE<br>VASWLFPNSDKNNNNQNNGLLFSDEYLNLVDYNSSMDYKFTGEYSQHQQ<br>NCSVPQTSYGGDRVVPLKLEESRGHQCHNQQNFQFNIKYGSSGTHYNDNG<br>SINHNAYISSMETGVVPESTACVTTASHPRTPKGTVEQQPDPASQMITVTQL<br>SPMDREARVLRYREKRKTRKFEKTIRYASRKAYAEIRPRVNGRFAKREIEA<br>EEQGFNTMLMYNTGYGIVPSF<br><br>>AtBBX2<br><br>MLKVESNWAQACDTCRSAACTVYCRADSAYLCSSCDAQVHAANRLASRH<br>ERVRVCQSCERAPAAFFCKADAASLCTTCDSEIHSANPLARRHQRVPILPIS<br>EYSYSSTATNHSCETTVTDPENRLVLGQEEEDEDEAEAASWLLPNSGKNSG<br>NNNGFSIGDEFLNLVDYSSSDKQFTDQSNQYQLDCNVPQRSYGEDGVVPL<br>QIEVSKGMYQEQQNFQLSINCGSWGALRSSNGSLSHMVNVSSMDLGVVPE<br>STTSDATVSNPRSPKAVTDQPPYPPAQMLSPRDREARVLRYREKKKMRKFE<br>KTIRYASRKAYAEKRPRIKGRFAKKKDVDEEANQAFSTMITFDTGYGIVPSF<br><br>>AtBBX3<br><br>MLKEESNESGTWARACDTCRSAACTVYCEADSAYLCTTCDARVHAANRV<br>ASRHERVRVCQSCESAPAAFLCKADAASLCTACDAEIHSANPLARRHQRVP<br>ILPLSANSCSSMAPSETDADNDEDDREVASWLLPNPGKNIGNQNNGFLFGV<br>EYLDLVDYSSSMDNQFEDNQYTHYQRSFGGDGVVPLQVEESTSHLQQSQQ<br>NFQLGINYGFSSGAHYNNNSLKDLNHSASVSSMDISVVPESTASDITVQHPR<br>TTKETIDQLSGPPTQVVQQLTPMEREARVLRYREKKKTRKFDKTIRYASRK<br>AYAEIRPRIKGRFAKRIETEAEAEEIFSTSLMSETGYGIVPSF<br><br>>AtBBX4<br><br>MASSSRLCDSCKSTAATLFCRADAAFLCGDCDGKIHTANKLASRHERVWL<br>CEVCEQAPAHVTCKADAAALCVTCDRDIHSANPLSRRHERVPITPFYDAVG<br>PAKSASSSVNFVDEDGGDVTASWLLAKEGIEITNLFSDLDYPKIEVTSEENS<br>SGNDGVVPVQNKLFLNEDYFNFDLSASKISQQGFNFINQTVSTRTIDVPLVP<br>ESGGVTAEMTNTETPAVQLSPAEREARVLRYREKRKNRKFEKTIRYASRKA<br>YAEMRPRIKGRFAKRTDSRENDGGDVGVYGGFGVVPSF<br><br>>AtBBX5<br><br>MASKLCDSCKSATAALYCRPDAAFLCLSCDSKVHAANKLASRHARVWMC<br>EVCEQAPAHVTCKADAAALCVTCDRDIHSANPLARRHERVPVTPFYDSVSS<br>DGSVKHTAVNFLDDCYFSDIDGNGSREEEEEEAASWLLLPNPKTTTTATAG<br>IVAVTSAEEVPGDSPEMNTGQQYLFSDPDPYLDLDYGNVDPKVESLEQNSS<br>GTDGVVPVENRTVRIPTVNENCFEMDFTGGSKGFTYGGGYNCISHSVSSSS<br>MEVGVVPDGGSVADVSYPYGGPATSGADPGTQRAVPLTSAEREARVMRY

REKRKNRKFEKTIRYASRKAYAEMRPRIKGRFAKRTDTNESNDVVGHGGIF
SGFGLVPTF

>AtBBX6

MGFGLESIKSISGGWGAAARSCDACKSVTAAVFCRVDSAFLCIACDTRIHSF
TRHERVWVCEVCEQAPAAVTCKADAAALCVSCDADIHSANPLASRHERVP
VETFFDSAETAVAKISASSTFGILGSSTTVDLTAVPVMADDLGLCPWLLPND
FNEPAKIEIGTENMKGSSDFMFSDFDRLIDFEFPNSFNHHQNNAGGDSLVPV
QTKTEPLPLTNNDHCFDIDFCRSKLSAFTYPSQSVSHSVSTSSIEYGVVPDGN
TNNSVNRSTITSSTTGGDHQASSMDREARVLRYREKRKNRKFEKTIRYASR
KAYAESRPRIKGRFAKRTETENDDIFLSHVYASAAHAQYGVVPTF

>AtBBX7

MGYMCDFCGEQRSMVYCRSDAACLCLSCDRSVHSANALSKRHSRTLVCE
RCNAQPATVRCVEERVSLCQNCDWSGHNNSNNNNSSSSSTSPQQHKRQTIS
CYSGCPSSSELASIWSFCLDLAGQSICEQELGMMNIDDDGPTDKKTCNEDK
KDVLVGSSSIPETSSVPQGKSSSAKDVGMCEDDFYGNLGMDEVDMALENY
EELFGTAFNPSEELFGHGGIDSLFHKHQTAPEGGNSVQPAGSNDSFMSSKTE
PIICFASKPAHSNISFSGVTGESSAGDFQECGASSSIQLSGEPPWYPPTLQDNN
ACSHSVTRNNAVMRYKEKKKARKFDKRVRYASRKARADVRRRVKGRFV
KAGEAYDYDPLTPTRSY

>AtBBX8

MGYMCDFCGEQRSMVYCRSDAACLCLSCDRNVHSANALSKRHSRTLVCE
RCNAQPASVRCSDERVSLCQNCDWSGHDGKNSTTTSHHKRQTINCYSGCP
SSAELSSIWSFCMDLNISSAEESACEQGMGLMTIDEDGTGEKSGVQKINVEQ
PETSSAAQGMDHSSVPENSSMAKELGVCEDDFNGNLISDEVDLALENYEEL
FGSAFNSSRYLFEHGGIGSLFEKDEAHEGSMQQPALSNNASADSFMTCRTE
PIICYSSKPAHSNISFSGITGESNAGDFQDCGASSMKQLSREPQPWCHPTAQ
DIIASSHATTRNNAVMRYKEKKKARKFDKRVRYVSRKERADVRRRVKGRF
VKSGEAYDYDPMSPTRSY

>AtBBX9

MEARCDFCGTEKALIYCKSDSAKLCLNCDVNVHSANPLSQRHTRSLLCEK
CSLQPTAVHCMNENVSLCQGCQWTASNCTGLGHRLQSLNPYSDCPSPSDF
GKIWSSTLEPSVTSLVSPFSDTLLQELDDWNGSSTSVVTQTQNLKDYSSFFP
MESNLPKVIEEECSGLDLCEGINLDDAPLNFNASNDIIGCSSLDNTKCYEYE
DSFKEENNIGLPSLLLPTLSGNVVPNMSLSMSNLTGESNATDYQDCGISPGF
LIGDSPWESNVEVSFNPKLRDEAKKRYKQKKSKRMFGKQIRYASRKARAD
TRKRVKGRFVKSGETFEYDPSLVM

>AtBBX10

MSPSMEPKCDHCATSQALIYCKSDLAKLCLNCDVHVHSANPLSHRHIRSLI
CEKCFSQPAAIRCLDEKVSYCQGCHWHESNCSELGHRVQSLNPFSGCPSPT
DFNRMWSSILEPPVSGLLSPFVGSFPLNDLNNTMFDTAYSMVPHNISYTQNF
SDNLSFFSTESKGYPDMVLKLEEGEEDLCEGLNLDDAPLNFDVGDDIIGCSS
EVHIEPDHTVPNCLLIDKTNTSSFTGSNFTVDKALEASPPGQQMNINTGLQL
PLSPVLFGQIHPSLNITGENNAADYQDCGMSPGFIMSEAPWETNFEVSCPQA

RNEAKLRYKEKKLKRSFGKQIRYASRKARADTRKRVKGRFVKAGDSYDY
DPSSPTTNN

>AtBBX11

MEAEEGHQRDRLCDYCDSSVALVYCKADSAKLCLACDKQVHVANQLFAK
HFRSLLCDSCNESPSSLFCETERSVLCQNCDWQHHTASSSLHSRRPFEGFTG
CPSVPELLAIVGLDDLTLDSGLLWESPEIVSLNDLIVSGGSGTHNFRATDVPP
LPKNRHATCGKYKDEMIRQLRGLSRSEPGCLKFETPDAEIDAGFQFLAPDLF
STCELVIETAIGCVFLCLSNDFPAYKSLLSI

>AtBBX12

MGTSTTESVVACEFCGERTAVLFCRADTAKLCLPCDQHVHSANLLSRKHV
RSQICDNCSKEPVSVRCFTDNLVLCQECDWDVHGSCSSSATHERSAVEGFS
GCPSVLELAAVWGIDLKGKKKEDDEDELTKNFGMGLDSWGSGSNIVQELI
VPYDVSCKKQSFSFGRSKQVVFEQLELLKRGFVEGEGEIMVPEGINGGGSIS
QPSPTTSFTSLLMSQSLCGNGMQWNATNHSTGQNTQIWDFNLGQSRNPDE
PSPVETKGSTFTFNNVTHLKNDTRTTNMNAFKESYQQEDSVHSTSTKGQET
SKSNNIPAAIHSHKSSNDSCGLHCTEHIAITSNRATRLVAVTNADLEQMAQN
RDNAMQRYKEKKKTRRYDKTIRYETRKARAETRLRVKGRFVKATDP

>AtBBX13

MSSSERVPCDFCGERTAVLFCRADTAKLCLPCDQQVHTANLLSRKHVRSQI
CDNCGNEPVSVRCFTDNLILCQECDWDVHGSCSVSDAHVRSAVEGFSGCP
SALELAALWGLDLEQGRKDEENQVPMMAMMMDNFGMQLDSWVLGSNE
LIVPSDTTFKKRGSCGSSCGRYKQVLCKQLEELLKSGVVGGDGDDGDRDR
DCDREGACDGDGDGEAGEGLMVPEMSERLKWSRDVEEINGGGGGGVNQ
QWNATTTNPSGGQSSQIWDFNLGQSRGPEDTSRVEAAYVGKGAASSFTIN
NFVDHMNETCSTNVKGVKEIKKDDYKRSTSGQVQPTKSESNNRPITFGSEK
GSNSSSDLHFTEHIAGTSCKTTRLVATKADLERLAQNRGDAMQRYKEKRK
TRRYDKTIRYESRKARADTRLRVRGRFVKASEAPYP

>AtBBX14

MMKSLASAVGGKTARACDSCVKRRARWYCAADDAFLCHACDGSVHSAN
PLARRHERVRLKSASAGKYRHASPPHQATWHQGFTRKARTPRGGKKSHT
MVFHDLVPEMSTEDQAESYEVEEQLIFEVPVMNSMVEEQCFNQSLEKQNE
FPMMPLSFKSSDEEDDDNAESCLNGLFPTDMELAQFTADVETLLGGGDREF
HSIEELGLGEMLKIEKEEVEEEGVVTREVHDQDEGDETSPFEISFDYEYTHK
TTFDEGEEDEKEDVMKNVMEMGVNEMSGGIKEEKKEKALMLRLDYESVI
STWGGQGIPWTARVPSEIDLDMVCFPTHTMGESGAEAHHHNHFRGLGLHL
GDAGDGGREARVSRYREKRRTRLFSKKIRYEVRKLNAEKRPRMKGRFVKR
SSIGVAH

>AtBBX15

MMKSLANAVGAKTARACDSCVKRRARWYCAADDAFLCQSCDSLVHSAN
PLARRHERVRLKTASPAVVKHSNHSSASPPHEVATWHHGFTRKARTPRGS
GKKNNSSIFHDLVPDISIEDQTDNYELEEQLICQVPVLDPLVSEQFLNDVVEP
KIEFPMIRSGLMIEEEEDNAESCLNGFFPTDMELEEFAADVETLLGRGLDTE
SYAMEELGLSNSEMFKIEKDEIEEEVEEIKAMSMDIFDDDRKDVDGTVPFEL
SFDYESSHKTSEEEVMKNVESSGECVVKVKEEEHKNVLMLRLNYDSVIST

WGGQGPPWSSGEPPERDMDISGWPAFSMVENGGESTHQKQYVGGCLPSSG
FGDGGREARVSRYREKRRTRLFSKKIRYEVRKLNAEKRPRMKGRFVKRAS
LAAAASPLGVNY

>AtBBX16

MVVDVESRTASVTGEKMAARGCDACMKRSRASWYCPADDAFLCQSCDA
SIHSANHLAKRHERVRLQSSSPTETADKTTSVWYEGFRRKARTPRSKSCAF
EKLLQIESNDPLVPELGGDEDDGFFSFSSVEETEESLNCCVPVFDPFSDMLID
DINGFCLVPDEVNNTTTNGELGEVEKAIMDDEGFMGFVPLDMDLEDLTMD
VESLLEEEQLCLGFKEPNDVGVIKEENKVGFEINCKDLKRVKDEDEEEEEA
KCENGGSKDSDREASNDKDRKTSLFLRLDYGAVISAWDNHGSPWKTGIKP
ECMLGGNTCLPHVVGGYEKLMSSDGSVTRQQGRDGGGSDGEREARVLRY
KEKRRTRLFSKKIRYEVRKLNAEQRPRIKGRFVKRTSLLT

>AtBBX17

MTSHQNIKISEKIMISKYQEDVKQPRACELCLNKHAVWYCASDDAFLCHV
CDESVHSANHVATKHERVCLRTNEISNDVRGGTTLTSVWHSGFRRKARTP
RSRYEKKPQQKIDDERRREDPRVPEIGGEVMFFIPEANDDDMTSLVPEFEGF
TEMGFFLSNHNGTEETTKQFNFEEEADTMEDLYYNGEEEDKTDGAEACPG
QYLMSCKKDYDNVITVSEKTEEIEDCYENNARHRLNYENVIAAWDKQESP
RDVKNNTSSFQLVPPGIEEKRVRSEREARVWRYRDKRKNRLFEKKIRYEVR
KVNADKRPRMKGRFVRRSLAIDS

>AtBBX18

MRILCDACESAAAIVFCAADEAALCCSCDEKVHKCNKLASRHLRVGLADP
SNAPSCDICENAPAFFYCEIDGSSLCLQCDMVVHVGGKRTHRRFLLLRQRIE
FPGDKPNHADQLGLRCQKASSGRGQESNGNGDHDHNMIDLNSNPQRVHEP
GSHNQEEGIDVNNANNHEHE

>AtBBX19

MRILCDACENAAAIIFCAADEAALCRPCDEKVHMCNKLASRHVRVGLAEP
SNAPCCDICENAPAFFYCEIDGSSLCLQCDMVVHVGGKRTHGRFLLLRQRI
EFPGDKPKENNTRDNLQNQRVSTNGNGEANGKIDDEMIDLNANPQRVHEP
SSNNNGIDVNNENNHEPAGLVPVGPFKRESEK

>AtBBX20

MKIWCAVCDKEEASVFCCADEAALCNGCDRHVHFANKLAGKHLRFSLTSP
TFKDAPLCDICGERRALLFCQEDRAILCRECDIPIHQANEHTKKHNRFLLTG
VKISASPSAYPRASNSNSAAAFGRAKTRPKSVSSEVPSSASNEVFTSSSSTTT
SNCYYGIEENYHHVSDSGSGSGCTGSISEYLMETLPGWRVEDLLEHPSCVS
YEDNIITNNNNSESYRVYDGSSQFHHQGFWDHKPFS

>AtBBX21

MKIRCDVCDKEEASVFCTADEASLCGGCDHQVHHANKLASKHLRFSLLYP
SSSNTSSPLCDICQDKKALLFCQQDRAILCKDCDSSIHAANEHTKKHDRFLL
TGVKLSATSSVYKPTSKSSSSSSSNQDFSVPGSSISNPPPLKKPLSAPPQSNKI
QPFSKINGGDASVNQWGSTSTISEYLMDTLPGWHVEDFLDSSLPTYGFSKS
GDDDGVLPYMEPEDDNNTKRNNNNNNNNNNNTVSLPSKNLGIWVPQIPQT

LPSSYPNQYFSQDNNIQFGMYNKETSPEVVSFAPIQNMKQQGQNNKRWYD
DGGFTVPQITPPPLSSNKKFRSFW

>AtBBX22

MKIQCNVCEAAEATVLCCADEAALCWACDEKIHAANKLAGKHQRVPLSA
SASSIPKCDICQEASGFFFCLQDRALLCRKCDVAIHTVNPHVSAHQRFLLTGI
KVGLESIDTGPSTKSSPTNDDKTMETKPFVQSIPEPQKMAFDHHHHQQQQE
QQEGVIPGTKVNDQTSTKLPLVSSGSTTGSIPQWQIEEIFGLTDFDQSYEYM
ENNGSSKADTSRRGDSDSSSMMRSAEEDGEDNNNCLGGETSWAVPQIQSP
PTASGLNWPKHFHHHSVFVPDITSSTPYTGSSPNQRVGKRRRRF

>AtBBX23

MKIQCEVCEKAEAEVLCCSDEAVLCKPCDIKVHEANKLFQRHHRVALQKD
AASATTASGAPLCDICQERKGYFFCLEDRAMLCNDCDEAIHTCNSHQRFLL
SGVQVSDQSLTENSECSTSFSSETYQIQSKVSLNSQYSSEETEAGNSGEIVHK
NPSVILSP

>AtBBX24

MKIQCDVCEKAPATVICCADEAALCPQCDIEIHAANKLASKHQRLHLNSLS
TKFPRCDICQEKAAFIFCVEDRALLCRDCDESIHVANSRSANHQRFLATGIK
VALTSTICSKEIEKNQPEPSNNQQKANQIPAKSTSQQQQQPSSATPLPWAVD
DFFHFSDIESTDKKGQLDLGAGELDWFSDMGFFGDQINDKALPAAEVPELS
VSHLGHVHSYKPMKSNVSHKKPRFETRYDDDDEEHFIVPDLG

>AtBBX25

MKIQCDVCEKAPATLICCADEAALCAKCDVEVHAANKLASKHQRLFLDSL
STKFPPCDICLEKAAFIFCVEDRALLCRDCDEATHAPNTRSANHQRFLATGI
RVALSSTSCNQEVEKNHFDPSNQQSLSKPPTQQPAAPSPLWATDEFFSYSDL
DCSNKEKEQLDLGELDWLAEMGLFGDQPDQEALPVAEVPELSFSHLAHAH
SYNRPMKSNVPNKKQRLEYRYDDEEEHFLVPDLG

>AtBBX26

MAQVCHTCRHVTAVIHCVTEALNFCLTCDNLRHHNNIHAEHVRYQLCDN
CSMYPSILFCYEDGMVLCQSCYSHHYNCATNGHQTQVVFANMNNQHHDH
AHMPHVVHHNNNNNHQQQHVGGHQRRAEMFERSCHGDNNCERWMFAM
RCELCVASNSNAVVYCPTHNQILCDSCDRMIHSHEDAVPPHSRCKLCVICK
RPSRRFLIGGYQFNFPPVHPPAAEGIPVTPPTELPQQDINYDYLDDVDDFSW
FGR

>AtBBX27

MKKLLKTDRRSFCHQSLIFLVLLSGGIFYMLCIIIIENMERVCEFCKAYRAVV
YCIADTANLCLTCDAKVHSANSLSGRHLRTVLCDSCKNQPCVVRCFDHKM
FLCHGCNDKFHGGGSSEHRRRDLRCYTGCPPAKDFAVMWGFRVMDDDD
DVSLEQSFRMVKPKVQREGGFILEQILELEKVQLREENGSSSLTERGDPSPL
ELPKKPEEQLIDLPQTGKELVVDFSHLSSSSTLGDSFWECKSPYNKNNQLW
HQNIQDIGVCEDTICSDDDFQIPDIDLTFRNFEEQFGADPEPIADSNNVFFVSS

	LDKSHEMKTFSSSFNNPIFAPKPASSTISFSSSETDNPYSHSEEVISFCPSLSNN<br>TRQKVITRLKEKKRARVEEKKA<br><br>>AtBBX28<br><br>MGKKCDLCNGVARMYCESDQASLCWDCDGKVHGANFLVAKHTRCLLCS<br>ACQSLTPWKATGLRLGPTFSVCESCVALKNAGGGRGNRVLSENRGQEEVN<br>SFESEEDRIREDHGDGDDAESYDDDEEEDEDEEYSDDEDEDDDEDGDDEE<br>AENQVVPWSAAAQVPPVMSSSSSDGGSGGSVTKRTRARENSDLLCSDDEI<br>GSSSAQGSNYSRPLKRSAFKSTVVV<br><br>>AtBBX29<br><br>MGKKKCELCCGVARMYCESDQASLCWDCDGKVHGANFLVAKHMRCLLC<br>SACQSHTPWKASGLNLGPTVSICESCLARKKNNNSSLAGRDQNLNQEEEIIG<br>CNDGAESYDEESDEDEEEEEVENQVVPAAVEQELPVVSSSSSVSSGEGDQV<br>VKRTRLDLDLNLSDEENQSRPLKRLSRDEGLSRSTVVMNSSIVKLHGGRRK<br>AEGCDTSSSSSFY<br><br>>AtBBX30<br><br>MCRGFEKEEERRSDNGGCQRLCTESHKAPVSCELCGENATVYCEADAAFL<br>CRKCDRWVHS<br><br>>AtBBX31<br><br>MCRGLNNEESRRSDGGGCRSLCTRPSVPVRCELCDGDASVFCEADSAFLCR<br>KCDRWVHGANFLAWRHVRRVLCTSCQKLTRRCLVGDHDFHVVLPSVTTV<br>GETTVENRSEQDNHEVPFVFL<br><br>>AtBBX32<br><br>MVSFCELCGAEADLHCAADSAFLCRSCDAKFHASNFLFARHFRRVICPNCK<br>SLTQNFVSGPLLPWPPRTTCCSESSSSSCCSSLDCVSSSELSSTTRDVNRARG<br>RENRVNAKAVAVTVADGIFVNWCGKLGLNRDLTNAVVSYASLALAVETR<br>PRATKRVFLAAAFWFGVKNTTTWQNLKKVEDVTGVSAGMIRAVESKLAR<br>AMTQQLRRWRVDSEEGWAENDNV
Cucumis melo	>CmBBX1<br><br>MPFSSLTIFGNFPASMKSYTQPRAIMPPNGICSPCNANLNLTPKFRLLSLSAIPN<br>MLHFPNQPNLYFGSSSNYSGFSRLFGPLRRRDFQTRSRLTFVNCGAGATRAA<br>ATDHYSTLNVSRNATLQDIKNSYKKLARKYHPDVNKEPGSEDRFKEISAAYE<br>VLSDDEKRYLYDQLSEAGVQGDYGVMSRDSQGVDPFEIFDAFFGGSDGLFR<br>ERDGIGGINLNQRSEKIQSLDIHYALHLSFEESVFGGEQAIQFSFFETCGKCDG<br>TGAKSNSCIKLCANCHGRGGVVKTQKTPFGMMSQVSICSECGGDGKKITELC<br>RSCGGSGQLQSIKKMNLVIPPGVSDGATMKIQREGSYDKKRGMTGDLYIML<br>HIGEKHGIWRDGIHLYSNISIDYTEAILGTVVKVETVEGLKDLQIPAGVQPGD<br>RVRLPFMGIPDINKPSVRGDHLFIVNVQIPKRISDSERTKIKELALLKASTKND<br>EVYTHGLPLGIFDKYADKNQGNLVSSQEIKRHTSLWSSINKRQPREGFASIGI<br>EISKPSCCRPLKLHSSYTDSLIMVVLVTSLFLMGKNYFWTLFRRKYH<br><br>>CmBBX2

MPSSQESESNPRDLELKIMKNIKCELCDCRANAYCESDEASLCWSCDSNVHS
ANFIVEKHSRILLCQICQSPTPWTATGPKLGPTLSLCQFCVVPQNVASLQFHH
QDHLHSGSSTTRDFDHRHHHSGDDEENQVVPLSPPPPVSS

>CmBBX3

MHSFAAAWSGVPAATKPLCYSCKSATAVLFCRHDTAFLCLRCDAQIHTLSG
TRHPRVWLCEVCEQAPATITCNADAAALCTSCDADIHSVNPLARRHDRSAIQ
PFYDSPPSSSVASVFKFLIPTQHQHDAVQPDLKSEDIFFSDMDSLIDFDYPTAG
DGVVPEQSNPGTESTTPPTDYSTRNLSGFQLCSTRSKPDAMSYPSQNLSHSVS
SSSLDVGVVPDRNTASDASFPMGQLAEKAVQLRGMDREARVLRYREKKKN
RKFEKTIRYASRKAYAEIRPRVKGRFVKRSETNCEIERFYGSAGVGFMVGDG
QYGVVPSLRV

>CmBBX4

MKKLCELCGCVATVMCEADAAMLCWGCDSKVHGANFLVGKHLRVLLCHD
CQAPTPWNGSGPNLVATVAFCHNCVHKKRLNNGRRCEKACGSSNGGTAPIG
CDDDDDDDDDDDEIENQVVPWLSLSPS

>CmBBX5

MRILCDSCESAAATLFCAADEAALCAVCDTKVHMCNKLASRHVRVGLANPS
EVPRCDICENAPAFFYCEIDGSSLCLQCDVIVHVGGKRMHKRYLRLRQRVEF
PGDKRNDGKDQNVKPMEQVEKVKGQNEERGEIEKHEELRVSGVEKDYSNG
DGHSKRPNKVIDLNM

>CmBBX6

MKIQCDVCEKGDAAVFCTADEAALCDLCDHRVHHANKLASKHRRFSLLRP
DAGEAPVCDVCKERRGFLFCQQDRAILCRECDDPIHSANELTKKHDRFLLTG
IKLSASAALYAPPPSGEKQIESGGRVVSASKSKGSVKKVAAVSKVPTICTPNV
CVNAPTNITPAAVVNKGGGGQIATGGGGSASSISEYLMETLPGWHFEDFLDS
SVSPPFVEVGLSLLLLLFVCEVDNHKV

>CmBBX7

MASKLCDSCKSATATLFCRADSAFLCLGCDSKVHAANKLASRHARVWVCE
VCEQAPAHVTCKADAAALCLTCDHDIHSANPLARRHERVPVTPFYDTSNSD
NSLAVKPGAAINFLDDRYFSDVDADAADVSREEAEAASWLLPNPNPKAIESS
DLNSSKFEFPEMDPYLDLDYGHVDPKLEAQEQNSSGADGVVPVQSKGVHLS
SANDRCLGIDFTGTKSFPYGHNPQSISHSVSSSSIEVGVVPDGNAMTDVSNPY
TKPSTESSVQPLQISPADREARVLRYREKRKNRKFEKTIRYASRKAYAETRPR
IKGRFAKRTDIELDVDRVSGYGVVPSF

>CmBBX8

ELTAVSDYIVATSPSPLSLRFSYSKIHKSAQETRLNEMMSRKNIANAVGGKTA
RACDSCVRRRARWYCAADDAFLCQSCDTTVHSANPLARRHQRLRLNSSNSP
TLNSPSWVSGFTRKPRTPRPRPKSNDLIHVSVVPETDDATSHEDEHLEDQLLY
RVPTLDVNVSDFGGDVEKNLEREWFDIEEELGFVKVEESCWRIGEGFDSDQV
DFLMDFEEIKIPPLVVGVKNERHEDNEDDESGITNKNKRRVLSLRLDCEAVIA
AWGNLQSPWTNGQRPDFDPDQPWPNSMEVCEVRYGQRCGEHGLRTAAMG

DGGREARVSRYREKRRTRLFSKKIRYEVRKLNAEKRPRMKGRFVKRTPFPPQ
PTLPFITIN

>CmBBX9

MTTRKSVEKTAEGGKTARACDSCISKRARWYCAADDAFLCQACDASVHSA
NSLARRHERVRLQTASFRPSSDNSSAASWHQGFTKKPRSPRIGKTAPARKPFP
QVPEVSAKEESEEQEQLLLHRVPVLGADSKDGNLASFVGEKESSNGYLSYD
MDPAEFAADVESLLGNSLDNECFDMEELGLAASKDHSLTNDDYSLNSHEIIK
IEPDEIEELTPMLGSEADTMREPFELNFMDFGSNPTTCGEDEDKVMMEVMAV
VKNGELEMEETKIVKNKKKISLSLDSEAVIIAWGSRGTPWTSGDRPNLDLDY
YWPDYMGTYESDCYYQPYGEFGSGIGRHAVTGVEGEREARVSRYREKRRT
RLFAKKIRYEVRKLNAEKRPRMKGRMKAQPTSLFNNVGGQIGLIPHTYTLNV
S

>CmBBX10

MKIRCDVCDQTEASVFCYADEAALCHACDLHVHRANKLAGKHSRFSLLQPI
KKDSPPCDICQERRALVFCQQDRAILCRECDISIHETNEHTQKHNRFLLTGVK
LSSTCFSYQTSSSSNGCDIDAPMDVKTGSSNASCSKRPKMAAKDQQISTSHSA
EKATPPSTSNHLVDQDGQALSDGGSFSTSSISEYLETLPGWCVEEFLDPSAAA
AAAAAANRFCKLCSTLPQY

>CmBBX11

MKIQCNVCEMAEATVLCCADEAALCWACDEKIHAANKLASKHQRVPLSGS
SSQMPKCDICQEASGYIFCLEDRALLCRKCDVAIHTANTYVTGHQRFLLTGV
KVALEPTDPVACSSMAKSHSREKSTETKVRPPSEREFSMPSPTELSRSLSVLG
GSEDFMENRTLLTGSGDSGSGGFSQWQMDELISLTGFNQNYGYMDNGSSKA
DSGKLGDSDSSPVLRAADIELDDDDECSGQVPEASWTVPQIPSPPTASGLYW
PRSYHNSMDGAVFVPDICSSEKVQHCSRNGTFSKRRRQF

>CmBBX12

IIKFIASLLAISVFLPTKWISLSLKRPYSSNNCQTPLGLFQFLSSIFWPHPFKNNS
FPPSSFPFSSPKPLSLPFSNFHMLNNSTETTTTTTAMTGGRTARVCDSCLCKRA
RWFCAADDAFLCQSCDVSVHSANQLARRHDRIRLETSSFNSTTDHLPPTPWL
KGFTRKARTPRSNNNKISSSKASVFSIVPEIGNDNELGFSIDENDDEHHQFLGH
QQEVPVFDPLFDDQKLLLTHELEDFGDGFLPSEVDLAEFVADVENLLGKEEE
EEQQQQHDGNTIIVKVKDEELVQDCINKNNNHNGFWMDWDFKEEIEEEEED
EEELKIKNKKNIISLRLNYDAVITAWDAQSSPYTTGNRPQFDLDDCLEEWSGI
CSKGGRNVAVDDQEWRRNYNGIISNGEREARVSRYREKRRTRLFSKKIRYQ
VRKLNAEKRPRMKGRFVKRTTTT

>CmBBX13

MGIDGDSVVKGFGGGWGVVAKPCDSCKTGPAAVYCRPDSAFLCLPCDAKIH
CANKLASRHERVWMCEVCEQAPAVVMCKADAAALCVTCDADIHSANPLA
RRHERVPVEPFFDSTESVVKSSSVFNFLVPNETNAPVCDGAHHHEEVEVSSW
LLSNSFFNSKLVDGPEIKPPSGDHLFFNEMDSFIDFEYPNPVNNHSAINDSVVP
VQTKPLPTPVTNHTHSPENCYDIDFCRSKLNSFGYQPQSLSHSVSSSSLEVGV
VPEGNSMSEISYPMGQNVSTGADSGLRLSGPGNQATQLCGMDREARVLRYR
EKRKNRKFEKTIRYASRKAYAETRPRIKGRFAKRTDMLSEVDEIYGSAASSV
FLTDAQYGVVPTF

>CmBBX14

MGLDGATVLNGFPGRPCHFCKTHPAAVYCRPDSAFLCVSCDAKIHCANKLA
SRHERVWMCEVCEQAPAVVMCKADAAALCVTCDAEIHSANPLASRHERVP
VEPFFDTAESVVKSSSVLNFLVPNETNVCLGAHYHEEVEVASWLLSNSSFNS
KLVHGPEIKPLGGDHLFFTEMDSFIDFEYPNSVSDDHNDINDSVVPVQTKPHP
TPVINHSHSPENCYDIDFCRSKLNSFGYQPQSLSHSVSSSSLDVGVVPQANSM
SEISYPMGQTVDSGLPLSGSGNQATQLCGIDREARVLRYREKRKNRKFEKTV
RYASRKAYAETRPRIKGRFAKRTDMVSEVDEIYGSAASPVFLTDAQYGIVPT
F

>CmBBX15

MVCSKSTTGETVPCDFCNDQVAILYCRADSAKLCLFCDKHVHSANLLSRKH
VRSQICDNCRSEPVSIRCSTDNLVLCQECDWDAHGSCSVSAAHDRTPIEGFTG
CPSALELVSLWGFDLGDKKLEESEMLVQNWVCSQDLVMPIDSWASRASATA
FNDLIVPNDNPFLFANLNCTDAASMFKKQSPSCGKHKQVIYKQLVELLKRDF
EGGDDDDGDDTRDGDAGGEDVGLQSMVPETTNGDCYWPGDLEGRQIPKED
DGVFVGAAPPPLLQQQTSFTSLLTMPSHVGLKDSERSVDETGVWDSNPNRQS
TQIWDFHLGRLRGHKDANTFDDAYGTGNMGFTIKNFGEYLKETSPTSAKLL
GETYPINCSSVHDDIPSFNNNVNNTTLSQGAATCESMNMPNNKLKCGSKSLQ
AIKQPIIIKGDSILSTSTTKADLELLAQNRGNAMQRYKEKRKTRRYDKYIRYE
SRKARADTRKRVKGRFVKASEAPVFG

>CmBBX16

MKKCELCGHQARMFCESDQANLCWDCDEKVHCANFLVAKHSRSLLCHVC
QSPTPWAASGRKLTPTVSVCEGCVEVHDGKCEQDRRRDNEVEVDDGDDFV
DGEGFDSYEGGDSQEDDDGEDDDDEEEDDEDGENQVVPWSYASSSSPPPPP
TVTSSSEGEISAGAGVASKRMREYGVDLDSDDEIESCSAPQSTRPFFNDEATS
SSSLRPLKQARVAGPIQSTTSTPDDEACKAELKSTAVVRSIQRLQNRLPTDIN
DASKMIFGICKMSRDQNR

>CmBBX17

MKIQCDVCSKDEAMLFCTADDAALCSSCDHRLHHHQQPDLLSSNHHRFPLL
YPNNNNNNNSHFPLCDICQERRAFLFCQEDRAILCKDCDVAIHWANQVTRN
HQRFLLTGVKLSSAAFSLSSLPNSNSHHVGANVSSTPVSDSPSVAESSTATAS
AAHGYGSMNGMAEYLIEPLPEWHFEEFIDSSSTPTTAPHHHHLAFSKWN

>CmBBX18

MCKGVEQEETKAPVRQRLTTDDRAARGGDPVRCELCGSRASLYCEADEAY
LCGKCDKSVHNANFLALRHVRCLLCNTCQSHTQRYLLGASMEVVVPSIVSR
ERNFLNYHCDSDGFVQNCSQVLKTPCLFL

>CmBBX19

MLKTEDEYREGGGNGWAAGICEACERCPAEFICKADAASLCAACDAEIHSA
NPLARRHQRVPISSGGAMFRSVEEEDEEEAASWLLMNPGKNNNDNKNNNSN
NNGMFLLGGEDEDEEEDDDYLKFVEFNGNNEEDDEFERLKNNNYGGGGDS
VVPIDQFEGNKDHHHHLHHHLHHHHEQQQNHEILLEQSYGGLVDASEFFH
TSSKPSYSYNGFLTHAISVSSMEVGVVPESTTTTMSDISISNVRPPKGTIDLFSG

TTAAEVAVGIQMPAAQLSPMDREARVLRYREKKKTRKFEKTIRYASRKAYA
ETRPRIKGRFAKRTDVEVQLDRKYSNPLLPDAGYGIVPSF

>CmBBX20

MCRGVEEGNHGHGHGHSHGHGRGGCRQSPVIQKGDVSAAGVLMCELCNSK
ASLYCQADDAYLCRKCDKWVHGANFLALRHIRCILCDVCQNLTQKYLMGT
STEVLLPTIIACTKANDCNNNGNLNTCCSVMLKRPFLFL

>CmBBX21

MKIQCDVCEKAPATVICCADEAALCAKCDVEVHAANKLASKHQRLLLQCLS
TKLPKCDICQDKAAFIFCVEDRALFCQDCDEPIHSSGSLSANHQRFLATGIRV
AMSSSCTKDVDKVKMEPPNPKNPQVPAKVPSQQVPNFTSSWAVDDFLHFSD
LESSDKQKEQLEFGELEWLAEMGLFGEQVPQEALAAAEVPELPTSHSGNAIA
CRPTKSSTSYKKPRIEMVDDEEFFTVPDLG

>CmBBX22

MKIRCDVCDKEEASVFCPSDEAALCPACDRQVHRANKLASKHSRFSLLHSA
ASAAATSQPLCDVCQIQRAFLFCREDRAILCRECDIPIHDTSEHTQKHSRFLLT
GVKVSPSTATSSSCSSSVGSGGEENEGLLKKCSRKRSKMGFSKGLVISEYLES
LPGWCVEDFLDSSSSPHILL

>CmBBX23

MGYICDFCAQQRSIVYCRSDAAALCLSCDRNVHSANALSRRHSRTLLCDRC
HSQAAFVRCPEENISLCQNCDYMGHSSSASISSRKRQPINCYSGCPTAAELSSI
WSFVLDLPSGSDACEQELGLMSIAENSAVNAWGPNDKAGQNVSGVDETNEF
SSVDKSIWYGSSSMPHIMDQPITMDATSPKLHYPGTKGPELDIEDDLYESLN
MDEDSLNIENYEELFGVSLSYSEELLENGGIDSLFRMKNLSAAKSGCPGGAA
AEGASVSFVNNVMQPASSNAASADSVMSAKTEPVLCYNNKQEHSGLSFSGM
TGESSAGEHQDCGASSMLLMGEPPWCSMATETSFQSSNRSDAVMRYKEKK
KARKFEKKVRYASRKVRADTRRRVKGRFVKAGEAYDYDPLSQTRSY

>CmBBX24

MGFMCDFCGDQRSMVYCRSDAACLCLSCDRNVHSANALSRRHTRTLLCER
CHLQPSTVRCIEERVSLCQNCDWTGHGTSTLASSSHKRQTINCYSGCPSAAEL
SCIWSFVLDVPSVNDACEKELGLMSIAETDLTGAWSLSENNAGQRMPRSTEA
SDVCSREKSNVLVGSSSLFGSRPHTSDQSVELDNVALPKDLLRHHIFGMQFC
CPGTKVAEFCGEDADLYKEFDMDEMDLNLENYEDLFSMSLNHSEEFFENGG
IDRFFEAKGLSFEDSVSHSAVAAEGSSVGVVQQMQPAYSNGASADSVMSTK
TEPILCFNSRQAQSGMSFSGLTGESSAGDHQDCGASSMFLMGEPPWCAPSTE
SSFPSTDRNSAVQRYKEKKKTRKFEKTVRYATRKARADVRRRVKGRFVKAG
EAYDYDPLNQARSC

>CmBBX25

MMMKMWMYHYQIRLQKTKDASYTSLSKRNGMENVEAHPENYCHNDKYPF
CSTCSNKAYEENVIFESEDEDEEEEEDVGENSKSFDSSVS

Cucumis sativus

>CsBBX1

MGIDGATVVNGFRGRPCGFCKADPAAVYCRPDSAFLCLSCDAKIHCANKLA
SRHDRVWMCEVCEQAPAVVTCKADAAALCVTCDADIHSANPLASRHERVP
VEPFFDTAESVVKSSSVLNFLVPDETNVCDGVHHHEEVEVASWLLSNPSFNS
KLVHGPEIKTQLGGDHLFFTEMDSFIDFEYPNSVNDDHNDIKDSIVPVQTKPD
PTPVINHTHSPENCYDIEFCRSKLNSFGYQPQSLSHSVSSSSLDVGVVPQAISM
SETSYPMGGQTGDSGLPLSGSGNQATQLCGMDREARVLRYREKRKNRKFEK
TVRYASRKAYAETRPRIKGRFAKRTDMLSEVDEMYGSAASHVLLTDAQYGL
VPTFCP

>CsBBX2

MGIDGDSVVKGFGGGWGVVAKPCDSCKTGPAAVYCRPDSAFLCLPCDAKIH
CANKLASRHERVWMCEVCEQAPAVVMCKADAAALCVTCDADIHSANPLA
RRHERVPVEPFFDSTESVVKSSSVFNFLVPNETTAPVCDGAHHHEEVEVSSW
LLSNSFFNSKLVDGPEIKPPSGDHLFFNEMDSFIDFEYPNPVNNHSAINDSVVP
VQTKPLLTPVINQTHSPENCYDIDFCRSKLNSFGYQPQSLSHSVSSSSLEVGVV
PEGNSVSDISYPMGQNVSTGADSGLPLSGSGNQATQLCGMDREARVLRYRE
KRKNRKFEKTIRYASRKAYAETRPRIKGRFAKRTDMLSEVDEIYGSAASSVF
LTDAQYGVVPTF

>CsBBX3

MTTSTAMTGGRTARVCDSCLCKRARWFCAADDAFLCQSCDVSVHSANQLA
RRHDRIRLETSSFNSTDHLPPTPWLKGFTRKARTPRSNNNNNKISSSKASVFSI
VPEIGNDNELGFSIDENDDEHHQFLGHQQEVPVFDPLFDDQKLLLTDELEDF
GDGFLPSEVDLAEFVADVENLLGRQDDEEQQQQDHDGNTNIIVKVKDEDLV
QDCINKNNNHNGYLMDWDFKEEIEEEEEEELKIKNKKNIISLRLNYDAVITA
WDAQSSPYTTGNRPQFDLDDCWEEWSGVCSKGGRNVVVDDQEWRGRNNN
GISNEEREARVSRYREKRRTRLFSKKIRYQVRKLNAEKRPRMKGRFVKRTTT

>CsBBX4

MKNIKCELCDCRANAYCESDESSLCWSCDANVHSANFIVEKHSRILLCQICQ
SPTPWTATGPKLGPTLSLCQLCVLPQNVASLRFRHQDPLHSGSSTTRDFDHHP
NEDDEENQVVPLSPPPVSS

>CsBBX5

MKIRCDVCDKEEASVFCPSDEAALCAPCDRQIHRANKLASQHNRFSLLHSSA
SASAAATSEPLCDICQIRRAFLFCREDRAILCRECDIPIHDTSEHTQKHSRFLLT
GVKVSPSPATSSSCSSSVASSGEENEGSLKKCSRKRSKMGFSKGLVISEYLESL
PGWCVEEFLDSSSSPHLFL

>CsBBX6

MKIQCDVCEKGDAVVFCTADEAALCNLCDHRVHHANKLASKHRRFSLLRP
DAGEAPVCDVCKERRGFLFCQQDRAILCRECDDPIHSANELTKKHDRFLLTG
IKLSASAALYAPSPSGEKPIGSGGCVVSASKSKGSVKKVAAVSKAPTICTPNV
CVNAPTNITPAAVVNKGGGGQIATGGGGSASSISEYLMETLPGWHFEDFLDS
SVSPPFVEFDDGIGFPFVEGDLNGCFSSSERIELWVPQGPPPAPYNSGLMMNN
GLKDTKDLGVNSSKVNRSVWTDDGFTVPQITSTVPSPGFKRSRPFW

>CsBBX7

MASKLCDSCKSATATLFCRADSAFLCLGCDSKVHAANKLASRHARVWVCE
VCEQAPAHVTCKADAAALCLTCDHDIHSANPLARRHERVPVTPFYDTSNSD
NSLPVKPSAAINFLDDRYFSDVDADAADVSREEAEAASWLLPNPNPKAIESS
DLNSGKFEFPEMDPYLDLDYSHVDPKLEAQEQNSSGADGVVPVQSKGVHLS
SANDRCLGIDFTGTKSFPYGHNPQSISHSVSSSSIEVGVVPDGNAMTDVSNPY
TKPSTESSVQPLQISPADREARVLRYREKRKNRKFEKTIRYASRKAYAETRPR
IKGRFAKRTDIELDVDRVSGYGVVPSF

>CsBBX8

MEPLCEFCGVVRAVVYCKSDSARLCLQCDGRVHSANSLSRRHLRSLLCDNC
NAQPAIVRCMDDKLSLCQSCDWNNNPNSHPNPSPNAPPPSHILHSYSGCPSM
PDFFRFWSSDPSSLPPPHSWFPPPPPPHFPFSTTPFHLSDPHSKGCSDFKDVTTT
TTAADATHDHDDLCEALNLDDVSLHLDNNEDELFGCPQGTTIKCQFEDGEL
DSLLMEKNFFEVTDSNAPPPPPLDNTIEDISSVQQDFIGFQSSQEGVSINMIQN
GNSNCMLMNPSCNGNINIGFPPTAQVHSSISLSLSNMTGESSVADYQDCGLSP
AFLTEASWDPSMEGIGPQAKDRNRDKAKMRYNEKKKTRTFGKQIRYASRK
ARADTRKRVKGRFVKAGEAYDYDPLVTRNF

>CsBBX9

MCRGVEEGNHGHAHGRDGCRQSPVIQKGVVSAAGVVMCELCNSKASLYCQ
ADDAYLCRKCDKWVHGANFLALRHIRCILCNVCQNLTQKYLMGTSTEVLLP
TIIACAEANDCNNNGNRNPCCSVMFKRPFLFL

>CsBBX10

MHSFAAPWSGVPASTKPLCCSCKSATAALFCRHDTAFLCLRCDAQIHTLSGT
RHPRVWLCEVCEQAPATITCNADAAALCPSCDADIHSVNPLARRHDRSAIQP
FYDSPPSSSVASVFKFLIPTQHQHDAVQPDLKSEDIFFSDMDSLIDFDYPTAGD
GVVPEQSNPGTESTTQLTDSSTRNFSGFQLCSTRSKLDAISYPSQNLSHSVSSS
SLDVGVVPDRNTASDASFPTVEKAVQLRGMEREARVLRYREKKKNRKFEKT
IRYASRKAYAEIRPRVKGRFVKRNETNCEMERIYGSAGVGFMVGEGQYGVV
PSLRV

>CsBBX11

MIKCELCGHQARMFCESDQANLCWDCDEKVHCANFLVAKHSRTLLCHVCQ
SPTPWAASGRKLTPTVSVCEGCVQVHDGKCDQERRRENEVEVDDGDDFVD
DEGFDSYEGGDSEEDDDGEEDDDEEEDDEDGENQVVPWSYASSSSPPPPPTV
TSSSEGEISAGAGVASKRMREYGVDLDSDDEIESCSAPQSTRPFFNDEATSSSS
LRPLKQARVTGPTQSITSTPDDEACKAELKSTAVVRSIQRLQNRLPTDINDAS
KMIFGICKMSRDQNR

>CsBBX12

MVCSKSTTGETVPCDFCNDQVAILYCRADSAKLCLFCDKHVHSANLLSRKH
VRSQICDNCRSEPVSIRCSTDNLVLCQECDWDAHGSCSVSAAHDRTPIEGFTG
CPSALELVSLWGFDLGDKKLEESEMLVQNWVCSQDLVMPIDSWASRASATA
FNDLIVPNDNPFLFANLNCTDAASMFKKQSPSCGKHKQVIYKQLVELLKRDF
EGGDDVEGDDTRDGDAGGEDVGLQSMVPETTNGDCYWQGDLEGRQISKED
DGVFVGAAPPPLLQQQASFTSLLTMPSHVGLKDNERSVDETGVWDSSPNRQ
STQIWDFHLGRLRGHKDSNTFDDAYGTGDMGFTIKNFGEFLKETSPTSAKLL

GETYQINCSSVHDDIPSFNNNVNNTTLSQGAVTCESINMPNDKLKGGSKSFQ
AIKQPIIIKGDSILSTSTTKADLELLAQNRGNAMQRYKEKRKTRRYDKYIRYE
SRKARADTRKRVKGRFVKANEAPVFG

>CsBBX13

MRTLCDACEKAAAIVFCAADEAALCRSCDEKVHMCNKLASRHVRVGLANP
SDVPRCDICENAPAFFYCEIDGSSLCLQCDMIVHVGGKRTHKRYLLLRQRVE
FPGDKPINLDDPSPHSKVPNEIGKVHNQPPPHKVTVEDNQQNHHRLSPVREA
NDDGHAETDTKMIDLNMKPHRVHGQAANNQDL

>CsBBX14

MKIQCNVCEMAEATVLCCADEAALCWACDEKIHAANKLASKHQRVPLSGS
SSQMPKCDICQEASGYIFCLEDRALLCRKCDVAIHTANTYVTGHQRFLLTGV
KVALEPTDPVACSSMAKSHSREKSTEIKIRPPSEREFAMPSPSELSRSLSVLGG
SEDFMANRTLLTGSGDSGSGGFSQWQMDELISLTGFNQNYGYMDNGSSKAD
SGKLGDSDSSPVLRAADIELDDDDECLGQVPEASWAVPQIPSPPTASGLYWP
RSYHNSMDGAVFVPDICSSEKVQHCSRNGTFSKRRRQF

>CsBBX15

MLKTEDEYRGSGGNGWGAVICEACERCPAEFICKADAASLCAACDAEIHSA
NPLARRHQRVPISRGGAMFRSVEEEDEEEAASWLLMNPGKNNDNKNNNNN
NNNGMFLLSGEDEEDDEYLKFVEFNGNNEEDDDEFETLKNNNYGGGGDSV
VPIDQFEGNKNHDHHLHHHHHEQQQQNHEILLEQSYGGLVDASEFFHTSSKP
SFSYNGFLTHAISVSSMEVGVVPESTATIMSDISISNMRPPKGTIDLFSGMIAA
EPAAASQMPAAQLSPMDREARVLRYREKKKTRKFEKTIRYASRKAYAETRP
RIKGRFAKRTDVEVQLDRKYSNPLMPDAGYGIVPSF

>CsBBX16

MTTRKSVERTAEGGKTARACDSCISKRARWYCAADDAFLCQACDASVHSA
NSLARRHERVRLQTASFRPSSDNSSAASWHQGFTKKPRSPRMGKTAPTRKPF
PQVPEVSAKEESEEQEQLLLHRVPVLGADSKDGNLASFVGEKESSNGYLSYD
MDPAEFAADVESLLGNSLDNECFDMEELGLVASKDHSLTNDDYSLNSHEIIK
IEPDEIEVLTPMLGIEADTMREPFELNFMDFGSNPTTCSEEDDKMMMEVMAV
VKNGELEMEETKIVKNKKKVSLSLDSEAVIIAWGSRGTPWTSGDRPNLDLDY
YWPDYMGTYESDCYYQPYGEFGSGIGRQAVTGVEGEREARVSRYREKRRT
RLFAKKIRYEVRKLNAEKRPRMKGRFVKRSSCFAPPPLPLFNQ

>CsBBX17

MERTCEFCAALRPIIYCTPDAAHLCLPCDAKVHSANALSSRHLRTLLCEFCRS
FPTYLQCLDHQMFLCRGCDRTLHVSSSQHQKRIIRGYMGCPSAKDFAALWG
FHVHEVDKAKFVSTSGSESSSVVKTFDAPGRSRSHIAAAENKVRYKGQEKGT
SFILQQILELSRLQLVKKNIHSPLILGEGKDGATSLKTCASEKFEQSLNEHVHH
SEDRSTGIQQRDNLLQELKMTSFTQLESFPMSSPILLPFHGESLWHCKSPAESS
QLWSQNMQDLGVCDELVCRDDFNMPEVDLTFQNFDEIFNSDQDPTGGLFDN
KDESYSYSSMDKDMSLSKSDNRDGKGVEASSATSSSCIFSYALMDKDSEPSD
EVCNHPMSTKIESARPIQPSLSTLSFADSRMSLDSAATDFPDRARGEPSCSSPY
HRDRKHSVSLNNVDAATKIYKEKQQFQLQEKQIRRKARSLVKKRVKGRYEK
GERYDSSTVAFSRSY

>CsBBX18

MRILCDSCESAPATLFCAADEAALCAICDTKVHMCNKLASRHVRVGLANPS
EVPRCDICENAPAFFYCEIDGSSLCLQCDVIVHVGGKRMHKRYLRLRQRVEF
PGDKQNDVKDLNVKPTEQVEKVKSQNEERGENEKHEELRVSGVTKDYSNG
DGHSKRPNKVIDLNM

>CsBBX19

MKKLCELCGCVATVMCESDAAMLCWGCDSKVHGANFIVGKHLRVLLCHD
CQAPTPWNGSGPNLVATVAFCHNCVHKNRRNNGRRCEKACGSSDGGGTTA
PSGCDDDDDEIENQVVPWLSLSPS

>CsBBX20

MMSRKNIANAVGGKTARACDSCVRKRARWYCAADDAFLCQSCDTTVHSA
NPLARRHQRLRLNASNSSPLNSPSWVSGFTRKPRTPRPRPKSNDLIHVSVVPE
TDDATSHENEHLEDQLLYRVPNLDVNVSDFGGDVERNLEREWFDIEEELGL
VKVEEDCWRIGEGFDSDQVDFFMDFEEIKMPPLVVGVKNERHEDEGDESGIT
DKNKRRVLSLRLDCEAVIAAWGNLQSPWTNGQRPDFDPDQPWPNSMDLCE
VRYGQRCGEYGFRTAAIGDGGREARVSRYREKRRTRLFSKKIRYEVRKLNA
EKRPRMKGRFVKRTPFAPQPTLPIITIN

>CsBBX21

MKIQCDVCEKAPATVICCADEAALCAKCDVEVHAANKLASKHQRLLLQCLS
TKLPKCDICQDKAAFIFCVEDRALFCQDCDEPIHSSGSLSANHQRFLATGIRV
AMSSSCTKEVDKVKMEPPNPKNPQVPAKVPSQQVPNFTSSWAVDDFLHFSD
LESSDKQKEQLEFGELEWLAEMGLFGEQVPQEALAAAEVPELPTSYSGNAIA
CRPTKSSTSYKKPRLEMVDDEEFFTVPDLG

>CsBBX22

MKEAFLSHFSVHSSYAHIHISSSLSVTLNSIHLNTKQNSTNLHLLGFLSFFLSFF
LSFLNYYPKPLLQNSPLFSSVLERNSPYLAVPEEATLWSENLNSERSEKSIAK
MGFMCDFCGDQRSMVYCRSDAACLCLSCDRNVHSANALSRRHTRTLLCER
CHLQPSTVRCIEERVSLCQNCDWTGHGSSTLASSSHKRQTINCYSGCPSAAEL
SCIWSFVLDVPSVNDACEKELGLMSIAETDLTGAWSPSENNAGQRMPGSTEA
SDVCSREKSNVLVGSSSLIGSRPHTSDQPVELDNVALPKFCCPGTKVAEFCGE
DDDLYKEFDMDEMDLNLENYEDLFSMSLNHSEEFFENGGIDSFFEAKGLSFE
DSVSHSAVVAEGSSMGVVQQMQPAYSNGASADSVMSTKTEPILCFNSRQAQ
SGMSFSGLTGESSAGDHQDCGASSMLLMGEPPWCAPGTESSFPSTDRNSAVQ
RYKEKKKTRKFEKTVRYATRKARADVRRRVKGRFVKAGEAYDYDPLNQEG
SISPEDERWIRKGGVHRRFND

>CsBBX23

MGYICDFCAQQRSIVYCRSDAAALCLSCDRNVHSANALSRRHSRTLLCDRC
HSQAAFVRCPEENISLCQNCDYMGHSSSASISSRKRQPINCYSGCPTAAELSSI
WSFVLDLPSGSDACEQELGLMSIAENSAVNAWGPNDKAGQNVSGVDETNEF
SSVDKSIWYGSSSMPHIMDQPITMDATSPKLHYPGRKGPEIDIEDDLYESLNM
DEDSLNIENYEELFGVSLSYSEELLENGGIDSLFRMKNLSAAKSGCPGGAAAE
GASVSFVNNVMQPANSNAASADSVMSAKTEPVLCYNNKQEHSGLSFSGMT

GESSAGEHQDCGASSMLLMGEPPWCSMATETSFQSSNRSDAVMRYKEKKK
ARKFEKKVRYASRKVRADTRRRVKGRFVKAGEAYDYDPLSQTRSY

>CsBBX24

MKIRCDVCDQTEASVFCYADEAALCHACDLHVHRANKLAGKHSRFSLLQPI
KKDSPPCDICQERRALVFCQQDRAILCRECDISIHETNEHTQKHNRFLLTGVK
LSSTCFSYQTSSSSNACDIDAAMDVKTGSSNACSKRPKMAPKDQQISSTSHSA
EKATPPSTSNNYLVDQDGQALSDGGSFSTSSISEYLETLPGWCVEEFLDPSAA
AAAAAAAANRFY

>CsBBX25

MCKGVEQEETTQVAVRQRRTTDDGAAGGGDPVRCELCGSRASLYCEADEA
YLCGKCDKSVHSANFLALRHVRCLLCNTCQSHTQRYLLGASMEVVLPPSLV
SRERNFLNYHCDSDSFVQNCSQVLKTPCLFL

>CsBBX26

MKIQCDVCSKDEAMLFCTADDAALCSSCDHRLHHQQQPDLLSSNHHRFPLL
YPNNNNNNHFPLCDICQERRAFLFCQEDRAILCKDCDVAIHWANQVTRNHQ
RFLLTGVKLSSAAAFSLSSLPNSNSHLVGANNVSSTPVSHSPSVAESSTATAS
AAHGCGSMNGVAEYLIEPLPEWHFEEFLDSSSTTTTNAPPPHPLAFSKSDDGD
CIFPFVDAVVELEITTDSLSLEHWRISTSVDVLPLCSLLIRYEVEMEKRNECSS
DSDSGGDSTLVDTIKMFLKSPAFHCNGRPFALGILFVCLLTILNKKEVCSSISF
LSFIPYYILLNIVCVD

Solanum lycopersicum

>SlBBX1

MLKKENSGGLDGSSNYWARVCDSCRSVTCTIYCQADSAYLCADCDARIHAA
SLVTSRHKRVWVCEACERAPAAFLCKADAASLCASCDADIHSANPLAHRHH
RIPIITIPGTLYGPPAVETVGGDSMMISGSTGEGTEDDGFLSLTQDADDTIIDEE
DEDEDEAASWLLLNHPVKNNNKNNVNNNNNQTNNYDMLFGGEVVDDYLD
LAEYGGDSQFNDQYNVNQQQQQYFVPQMSYGGDSVVPVQDGQGKPLIFYQ
QQQQQQQSHHQNFQLGMEYDNSNTRLGYPASMSHSVSVVSMDVSVVPESA
LCETSNSQPRPQKGTIELFSGHPIQIPLLTPMDREARVLRYREKKKNRKFEKTI
RYASRKAYAETRPRIKGRFAKRTDVEAEVDQMFSTQLMTDSSYRIVPSF

>SlBBX2

MLKNENSGVFYGSRNNWSRVCDSCRSTACAVYCRADSSFLCAGCDTRMHA
ANLLASRHKRVWICEACERSPAAFLCKADAASLCTSCDADIHSASPLACRHH
RVPIMTILDTSQLTPMDREARVLKYREKKKNRKFEKTIRYALRKVYAETRPRI
KGRFAKRTDVAEEDQMLSTQLMADGIYGIVPS

>SlBBX3

MLKKENSNNWARVCDSCHSATCTVYCRADSAYLCAGCDARIHTASLMASR
HERVWVCEACERAPAAFLCKADAASLCASCDADIHSANPLARRHHRVPIMPI
PGTIYGPPAVHTITGGSMMIGGTTGEGTEDDGFLSLNQDADDTTIDEEDEDEA
ASWLLLNPPVKNNNKNNNYGMLFGGEVVDDYLDLAEYGGDSQFNDQYSV
NQQQQHYSVPQKSYVEDSVVPVQNGQRKSLILYQTPQQQQSHHLNFQLGME
YDNSNTGYGYPASLSHSVSISSMDVSVVPESAQSETSNSHPRPPKGTIDLFSGP

PIQIPPQLTPMDREARVLRYREKKKNRKFEKTIRYASRKAYAETRPRIKGRFA
KRTDVEAEVDQMFSTQLMTDSNYGIVPSF

>SlBBX4

MVAESWSTTAKRCDACKATPSTVFCKADMAFLCLTCDSKIHAANKLASRHA
RVWVCEVCEHAPASVTCKADAAALCTTCDQDIHSANPLARRHERIPVVPFY
DSASASSSRGAAADGNDDPQQHDDDTEEEEAEAESWLLQAPSTNNNTQGIE
YKSVEYLFSDVDPYVEMDIIADQKPSNDIAQLHNQEEYKEDCVVPHVQNNK
NDIQLQGPVVDGYPTYEIDFSGGSKPFMYNFTSQSISQSVSSSSMEVGVVPDH
NTMTDVSNTFVRNSAIDGLPNPVSSLDRKARVLRYREKRKNRKFEKTIRYAS
RKAYAETRPRIKGRFAKRTENEVGDSLVASDASYGVVPSF

>SlBBX5

MGTENWSLTAKLCDSCKTSPATVFCRADSAFLCLGCDCKIHAANKLASRHA
RVWVCEVCEQAPASVTCKADAAALCVTCDRDIHSANPLARRHERFPVVPFY
DFAVAKSHGGGDTDADAVDDEKYFDSTNENPSQPEEEAEAASWILPTPKEG
TDQYKSADYLFNDMDSYLDIDLMSCEQKPHILHHQQHQHNHYSSDGVVPV
QNNNETTHLPGPVVDGFPTYELDFTGSKPYMYNFTSQSISQSVSSSSLDVGVV
PDHSTMTDVSNTFVMNSSSGAIAGAGADVVPNAVSGLDREARVMRYREKR
KNRKFEKTIRYASRKAYAETRPRIKGRFAKRTETEIDSLITVDASYGVVPSF

>SlBBX6

KKKKKMGIFREAPNCFPGGWNIGAAARMAKSCEYCHLAAALVFCRTDNTF
VCLSCDTRLHARHERVWVCEVCEQAAASVTCRADAAALCVACDRDIHSAN
PLARRHERVPVVPFYDPVESVVKSTAATLLVSINGTTTTATTTATITPELGKV
DTCIGHHENNNDPWIPPNTITSKLPLNTEMKGMDFIFTDSENFLDFDYPACVD
TQSQPHYNSSNDSVVPVQANTPIKSLPFHHQEKHFEIDFTQSHIKSYNTPSLSV
SSSSLDVGIVPDGSSISEISYPYIRTMNNSNSSIDLSNSANHQGEKLLGLDREAR
VLRYREKKKNRKFEKTIRYASRKAYAETRPRIKGRFAKRTDGSAGAGEFDD
VDGIFSGTDFIAAESRYGVVPSFLT

>SlBBX7

MGYICEYCGEQRSIVYCRSDAACLCLSCDRNVHSANALSQRHSRTLICERCN
SQPAVVRCVEERISLCQNCDWSGHASSSSGSSMHKRQALNSYTGCPSAAELS
NIWSFLLDDPSIGDTCEQRMGSMSINDNRPRDGQDPQGKDNSQNVCAAVEA
NDMSISEKSNLLVESSMPTFDNKLHNMEPPIGSSSKGCYMGAKGSNLFEEDP
YCDNLIMDAVDLSIENYEELFGDSLNYPDELFENENLDSFFGMKDIKGADYS
YHGVNATEGLSNARVNTVQPTCSDAASADSIMSCKTDSILYFARQSSLSVSN
QTGGECSAGDHQDCGVSPMLLMGEPPWCPPCPEISSTSTSRSNAVLRYKEKK
KTRKFDKRVRYVSRKARADVRRRVKGRFIKAGDAYDYDPLPTRSY

>SlBBX8

MGHMCEFCGEQRSIVYCRSDAACLCLSCDRNVHSANALSQRHSRTLLCERC
NSQPAIVRRVEEKVSLCKNCDSIGHAGSGTGSVHNRQALSSYTGCPSAAELS
TIWSFLLDNSLGGDSTCEKGMGSMSITDNRLTDSRAPQGKFNSQDASATVEV
SEIHTPGKSSILVGSSMPNLGSKLNKVEHIAGSVNISSSKDCYSGVKASTIYED
DPFSQDFNMDEVDLSFENYEELFSGSLDNPNQLFENDDIDGLFGTKDMSVSD
SSCQDANAVEVTLTINIISSASVEIHANSVNDES

>SlBBX9

MGYICEYCGEQRSIVYCRSDSACLCLSCDRNIHSANALSQRHSRTLVCERCNS
QPAIFRCVEERVSLCQNCDWLAHASSGTCSTHKRQALSCYTGCPSAVELSTI
WSFILGDPSVCDSTCEQGMGSMSITDCQPGDSQHPQVKEKSQDISSDEAKDL
HNLVKSAPFMGSSMPSLDNELPNVELLVASTNLTWSKVKNSGTKGYNDPFY
DDFNMDEVDLSIENYKELFGVSIDNCDQLFKNEDIDDFFGMKDMSVAESSFQ
GVNAVEVVHTL

>SlBBX10

MDPLCDLCGEVRAVVYCKSDSARLCLQCDDYVHSPNLISRRHSRSFICDKCN
SQPSIVRCMDEAISLCERCDWDGNGCIGTGHRLKKLNPYTGCPSPDEFTKML
SQVLEMPIGTDTNFGSFGNSLGSSLSINENNSSLENKVNEDSFVSSKLLASNY
KFEAWSIPPEPNYLNSYQIDLAPFSEGSGLSKQDCPIKDLGLQEGDDLSKGVD
FDDVTLDFNCSYEILPDSRQTGFSDENKELDCLVMEKNSSVTGSNNVETSHE
ATSSVQQEYMGLQSSQISAAASSTNLLQTMSANANCMLMNSTCNGSIGALPF
LPAPIHPSMSLSLSNITGESCAEQDCGLSPGFLNEAPWDLSLENCPQKRHEAK
MRYNEKKKTRTFGKQIRYASRKARADTRRRVKGRFVKAGEAYDYDPSETR
DF

>SlBBX11

MTEVKKDEENHHQHLCDFCGNNTALLYCRADSAKLCFTCDREVHSTNQLFT
KHTRWLLCNLCDSSPASILCCTETSVLCQNCDWESHNKLLSLHERRPLEGFT
GCPSVSELLSILGFEDLGKKELLCGGDDGAYGFSDWVIWDAPSVVTLDDLIA
NNNESRHNYQAIGVPPLPKNRNAACGKHKEEILGQLRELSKLEPNSGDDQDE
NVPTTGFQSMEPVQNCPLRYKGSGFMQRSDQHVVPSSEGSAFHWHGDTGEF
VDQGFSSSLTDCFIETKCLLPDRDSDVCDASGGGNEEQSHHPTTTETFQMVP
KVVHRELNSQERETAVSRYKEKKKTRRYEKHIRYESRKARAETRTRIKGRFA
KMDYRDSSVHQ

>SlBBX12

MSPGESRPCDFCNQQIAVLYCRADTAKLCLFCDQLVHSANALSKKHLRSQIC
DNCGSEPVSIRCDTDKLVLCQECDWDAHGSCAVSGAHDRSPVEGFSGCPSAS
DLASAWGLDIESKKLHQQHTVLEYPSWMSKDAPPSSVLLQDLMVPSAINSAI
YSTKQTPTCGKQKQVIFKQLIELFKRDLADGVGAGAEDLVPKTPNATSDWQ
GNINVSIMDGVTQKPQQQQPQNVPFTSSIMPHNPKDSDQMVERNILWRGNSF
DQNTQIWDFNLGQLRSHEQSSSLEADYSESDMACMMKSYGELIKGTSLATS
KVLGLSGINCSVVHDDMTAFSNNSNNRAASQGPATSESNNLPRIKTSSDLGC
VKPKCGGVSTDLNFMDQSIVVGGDNTGEETLKADMELLAKNRGNAMQRYK
EKKKTRRYDKHIRYESRKARADTRKRVKGRFVKANESPDG

>SlBBX13

MSSEKKLANAMGAKTARACDNCIRKRARWYCAADDAFLCQSCDSSVHSAN
PLARRHERVRLKTSSFKSSDDFPNLESTVSGLGSGSGSGSDSIPSWHCGFTRK
ARTPRYGNKHAKRVKSTEEEEEEEEMKNPIQLVVPEILSDENSHDENEEEQLL
YRVPIFDPFMADGSNYGNEYSSNKVDFNQDMNTFQGLLAPSEMELAEFAAD
VVSLLGKGLDDEESFNYMEGLGFLEKHDEKLVKVEDEGEVGFVNMISTNNQ
VDYSEFDMVGETFELKFDYDSQVINNLDEDNKKVEFLEINYDSGKNNNKIM
LNLDYESVLKSWGDKRFPWTTGVRPEVDFNDCWPVCMGNCGKIHSYGDIAI

MNGHGGGVVDEGREARVLRYKEKRRTRLFSKKIRYEVRKLNAEKRPRMKG
RFVKRTNFAPTPFPSLNK

>SlBBX14

MMTSESKTANAIGGKTARACDSCLSKRARWFCPADDAFLCQSCDVSIHSAN
QLASRHERVRLETCSNKSTITKLVDKTHQPSWHQGFTRKARTPRNGKKAQIR
QWKKNEENRVPEIGSEENSLDENEFENEEQLLYRVPIFDPFEAELCNVPDETG
SIVDLDILLNTEDACHDLNLPEFLSSDIELAEFAADVETLLGGEEEQSTQLLNA
DFENNKAIKIEVEDEEMRAVVACHLDPELDMEREALNWNFDEYYEETVEQK
VMAADAAVTEFIASAEEYSGSSTKSDEKNRLFLSLNHEAVISAWPNQSSPWT
NGIRPHFNPDDYWPDFFSETSVGNYGGHVRCGDGGREARVSRYREKRQNRL
FSKKIRYEVRKLNAEKRPRLKGRFIKRSSSLFSVPGFSYMMNKR

>SlBBX15

MVSERKLASAMGGKTTRACDNCIKKRARWYCPADDAFLCQNCDASVHSAN
PLARRHERVRLKTSSLKQTSSPSSSSDDYFPDLESPLSISSVSVSVSVPSWHRG
FTRKARTPRQGRKASKSAGDGDVIRKNPIHLVPEILSDENSLDENEEEQLLYR
VPILDPFVGHLYSSSTAPTDADSEFKLESKEMTLQDDICNVDLNRFHEMLPSE
MELAEFAADVESLLGKGLDDESFDMEGLGLLGVCNKEENSMISHEKVKIED
EGEMEVVTKTTSPTTHNHQYNHTHDHDIDINEDTFEFKFDYDSSINIIGDDEV
VTNDENKKKILLNLDYEGVLKAWADQRCPWTNGERPELDSNESWPDCMGN
YMGIMNENVTIVDRGREARVTRYREKRRTRLFSKKIRYEVRKLNAEKRPRM
KGRFVKRANFVTTSTPNYPLVK

>SlBBX16

MCNGRREIDEEKIEELHNIIVCELCKSEAYVYCEADNAFLCKKCDKLVHTAN
FFAQRHIRCILCGICKKLTKRYLIGVSHEVILLKVVRCTNFDEQNCSTKVKEPF
LFL

>SlBBX17

MCSGRREGDEKTSSTSYCKGPSKEGESIISSAITCALCSSEASVYCEADNAFLC
RKCDRSVHGANFLAQRHIRCLLCSVCRKTTRRFLIGTSSELILPTIARLEQRNR
SRSAESETTDYRTTPQELFLFI

>SlBBX18

MRTLCDVCESAAAILFCAADEAALCRACDEKVHMCNKLASRHVRVGIAKPN
EVPRCDICENSPAFFYCEVDGSSLCLQCDMMVHVGGK

>SlBBX19

MRTLCDVCESAAAILFCAADEAALCRSCDEKVHLCNKLASRHVRVGLADPS
KIQRCDICENAPAFFYCEIDGSSLCLQCDMIVHVGGKRTHGRYLLIRQRIEFPG
DKLGPSNEQGLPSTEQGDVRRETAQPFKLPMIDNHQPNRETAMTAVENNVN
NSVKVENELIDLNSRPQRMHGQTSNNQEQVMDMLGGSNHESVGVVPDGPF
KREPEK

>SlBBX20

MKIQCDVCNKKEAIVFCTADEAALCDDCDHRVHHVNKLASKHQRFSLVQPS
PKQAPMCDICQERRGFLFCQQDRAIMCRECDIPIHKANEHTQKHNRYLLTGI

KLSANSALYSAPSQSQSQSQSAISSADSCVSNLKSKDSTSKPVAGSVFVSPAII
SNSTKGGAVSSAVESVKVVKEKVGGCNNNVQFVNGGGNNLTSSISEYLEML
PGWHVEDFLDCSTPNVYSKNIGDEDMLSFWDTDLESQFSSFPPQNVGIWVPQ
APPLQESKQETQIQFFPSQNLNFGGQIGLKESREVTNIKSSRKWTDDNSFAVP
QMKPPSTSFKRTRTLW

>SlBBX21

MKIQCDVCNNNEASVFCVADEAALCDSCDHRVHHANKLASKHQRFSLIQPS
PKQIPVCDICQERRAFLFCQQDRAILCRECDVSIHKANEHTQKHNRFLLTGVK
ISANSSLYTSSESVSAASCSANQDSVTNLNKPQICTKKTSPVSGSVPQQQVSV
AANIGENSYTSSISEYLEMLPGWHVEELLNASTIPTNGFCKIGDNDVFPIWDS
EIESSMNSFSPENIGIWVPQAPPALTPQKNQNQVFPRNINFGGQIEFKNMKEV
TSKKSSRKWRDDNSFAVPQISPSSSSISFKRSRTLW

>SlBBX22

MARRRLLLLLKPFDMLPSRHFDESSHFRNSKGWPALWRSVFKGGTTMVEGC
RVRLLLLQLSQVIKYLDSRSLVHMEAINFCQNILRKKHVDWEAVYRFNLCRP
IRDVDLVVTIGGDGTLLQASHFMDDSIPVLGVNSDPTQAEEVEDCNKEFDAT
RSTGYLCAATVKNFEQIIDDILENHARPSEVSRMSITHNSKQLPTYALNDVLIC
HPSPATVSRFSFSKKKEGQSSSSMVHCRSSGLRVSTAAGSTAAMLSSGGFAM
PILSRDLQYIVREPISPGAYNSAMHGTVKPEELMEIAWYCNEGLIYIDGSHIIH
SVQHGDIIELSCKAPKLKIFLPSHLISDDGCGLGFCSLDLQLQKHYYDEMWIT
FQSSSNHAKEIFCFFIRRSAEEVMKIQCNVCEVAEANVLCCADEAALCWSCD
EKVHAANKLASKHQRVPLSGSSSSMPMCDICQETVGYFFCLEDRALLCRKC
DIAIHTANPHVAAHQRFLLTGVKVGLEPVDPGGISSSGTSQSIQKVSEPESAPL
SKRNASVSLDAQFNKVLPTQVSGIEDFAPTKSPFAGGSAAGSMPQWQFDEFI
GLSDFNQNYGYMDDGSSKADNGKLGGESDSSSILRVEDEELDGDECLGQVP
DTSWAVPQVPSPPTASGLYWPKTYQNPFDSAVFVPDISYSPSSSLQQQPPSGT
RLKRRRQC

>SlBBX23

MKIQCNACEVAEAKVLCCADEAALCWYCDDKVHAANKLANKHQRVALSA
SSSPMPKCDICQETVGFFFCLEDRALLCRKCDISIHTVNAYVSSHQRFLLTGV
KVGLEPLGPCASASSRKSHEQRSPPILKITPPSEGVLPVHTSGNGNFAPSRLPIV
GNIPQCQFDQYLGMPDFNQNYGYMDYGQSKAGNGKVGESASSPFLRDVDA
EVAGDECFSTEVPDTCWAVPQIPSSPTASGLNWPTKSIQNPFDAALLESDASY
FPLQNIQDQQSNGSGTKRSRRF

>SlBBX24

MKIQCDVCEKAQATVICCADEAALCAKCDIEVHAANKLASKHQRLHLQCLS
NKLPPCDICQDKAAFIFCVEDRALFCKDCDEAIHSASSLAKNHQRFLATGIRV
ALSSSCNKESVKNQLQPQPPQQNSQQVGLKMPPQQLSCITSPSWPVDDLLGF
PDYESSDKKDLLELGEFEWLGGIDLFGEQTAAEVPELSVPQSSNTNIYRTTKY
QMPYKKSRIEIPDDDEYFTVPDLG

>SlBBX25

MKIQCDVCDKEEASVYCSADEATLCQSCDYQVHHANKLASKHLRFSLIHPSF
KDSPLCDICQERRALLFCKEDRAILCKECDLPIHKANEHTKKHNRFLLSGVQL

	SSDILASNYNNNQNSISPAGSAASNAGTNNFKALSGNFGMKSNSISSTTESTH<br>NYFHVDYVQEGSVSTSSISEYLTETLPGWHVEDFLEYPSSSSYEF<br><br>>SlBBX26<br><br>MMNCELCGENKSMMYCESDQANLCWDCDSKVHSANFLVAKHSRNLLCHS<br>CQNSTPWNASGPKLSPTFSLCNSCIQNPDPIEQIGETIEENYQTEADNDEDEYV<br>STDSEIDDYDDENQVVPLSSSPSSDFSPSPSPVRSVSSSGSYGDLSAIGDYGGG<br>ATAADNSSPWKRVRESDCLHFEGESVPPTLHPRIEISGSE<br><br>>SlBBX27<br><br>MEMEKACEFCMLLKPVVYCEADAAHLCLSCDAKVHSANALSNRHPRTLVC<br>ECCGHNPAYIRCSDHQTFMCRDCDRCHHDLSSQHQRKVITSYMGSPSAKDL<br>AALWGFGLKDLENATPPDQFISTSNGKANGVKVISKKFKRSHSSPGGSSLASE<br>LDFTGLVVSPESEVGSTSYYTKVLSLRKRRENTSLILQQILDLERLQLTEGSNN<br>LTSGESRNNVSSLKNCTSWNMHNKFDCLQSSLDLGPELQDWGSTHESPVDE<br>SFPLPLPDGDSFWECKSPVQSSQLWPQNLQDLGAYAELERFDNSNMPDVDL<br>TFQNFEELFGEDQYLNNTLLEEDMTCSSMENDSSIDRSDYSYVKKDISTASSV<br>RTGHSTHFGQAHGEHIPTIKDCPPPIRTNFSSLSFSASRLSSESSGNEYVDSPAA<br>NDQEVSCNSQMDSKKKARLYEKQARNTPRRARTNFKKQHVLKAHCYETDA<br>LNMSRSF<br><br>>SlBBX28<br><br>MKNCELCNGLARIYCESDHANLCWDCDLKVHSANFLVAKHSRSLLCNVCRS<br>PTVWSASGAKIGRTVSVCERCVNDEETDREEEEKEEIDLEDIQVVPWSSTPPP<br>QPESSSSSSDESLTDFFSLKRMRKDDDSGTLTSHREVNMLSPATEDHGGDEA<br>AEFVKSFPVKMPSKIQRTGEILSGRVENIGCSATVELIGRINRGGG<br><br>>SlBBX29<br><br>MKKCELCSSIARVYCESDQASLCWDCDARVHTANFLVAKHSRILLCNSCQSL<br>TPWTGSGSKLGPTVSVCQKYQPNPPSLTSSSSSSEDSTNRFNRRSIIGFSPNRTE<br>ENIQSHYCGITRPKEKIERSSWRWR<br><br>>SlBBX30<br><br>MELLSSKLCELCNDQAALFCPSDSAFLCFHCDAKVHQANFLVARHLRLTLCS<br>HCNSLTKKRFSPCSPPPPALCPSCSRNSSGDSDLRSVSTTSSSSSSTCVSSTQSS<br>AITQKINIISSNRKQFPDSDSNGEVNSGRCNLVRSRSVKLRDPRAATCVFMHW<br>CTKLQMNREERVVQTACSVLGICFSRFRGLPLRVALAACFWFGLKTTEDKSK<br>TSQSLKKLEEISGVPAKIILATELKLRKIMKTNHGQPQAMEESWAESSP<br><br>>SlBBX31<br><br>MKNCELCTGLARMYCESDNASLCWDCDAKVHSANFLAARHSRSLLCQVCQ<br>SPTAWSAAGAKLGKTVSVCDKCVDGYYHRDGVEEMEESESVNDEESDTED<br>EETDYEEDEDEIDSGDIQVVPWSNTTPPPPASSSSSEDSSNGCRNVSSKRMRE<br>ADPDLQSDDDSGSSSCQRKVHISPAMDADGGDDAAASVDSYSSKMRTPVKI<br>QRTDRLTAPRCTPVIEFIRRNNRQRMNSGAAIAELCRLNHESRTTDLKSSETS
Zea mays	>ZmBBX1

MGEGDDDRRDELLGAGRDHEPEPADAEEAKKPAPGEAEAGNGAGAEAATC
DYCGTAAAAVYCRADSARLCLPCDRLVHGANGVCSRHARAPLCAGCCAAG
AVFRRASTSAFLCSNCDFGRHRDGGDPPLHDRCAVQPYSGCPPASDLAALLA
VPLFDKPAAEDGAWWNIWEEPQVLSLEDLIVPTTPCHGFEPLLTPPSPKNRSI
SPDRKVNEEILRQLGELAESDGGMQASAGREEAEQAGGDQFPSWASPQYAT
GHGNFGTEDNHENGRWNNSEYHDLNDACKLEVTYDQAPVNSAEPCLSSFAP
LSEICPSMSNGSSKEDNHQANPGIGMPMQGLPKRSGFDVVPCPDRDSVISRY
KAKRKTRRFDRQVRYESRKVRADGRLRIKGRFAKANQT

>ZmBBX2

MTNAGAATGAALGARTARSCDSCMRRRARWHCPADNAFLCQSCDVSVHS
ANPLARRHHRVRLPSASCSSPPRDPDAPTWLHGLKRRPRTPRSKPGGSNKHE
AAPSSIAAAASAAVPDLEAEAEAEAEESGSGILGDNDDDHGFQDDDENLLYC
VPVFDPMLAEFYNPVADEGEQKPACLMLPLVETSPEFASGGLAEADGLSGFD
VPDMDLASFAADMESLLMGVDDGFDDLGFLDEQKPQVNADVDLEAMVAP
EPEREDKKRKRTDMILKLNYEGVIASWVRDGGSPWYHGERPHLDDPYELWL
EFPATGSRGLFGGTMTAVTGGEREARVSRYREKRRRRLFAKKIRYEVRKLN
AEKRPRMKGRFVKRTTLPPLPRPPPPSQQQQKKKQLPRSLPHVGMVLAPPPG
ADGRFRF

>ZmBBX3

MKVQCDVCTAEAASVFCCADEAALCDACDRRVHRANKLAGKHRRFSLLHP
CSSSSSSAAHKPPLCDICQERRGFLFCKEDRAILCRECDAPVHSASDMTRRHS
RFLLTGVRLSSAPVDSAGPSEEEEQENSRGPCNDESCSSGSGAGGATTATASD
GSSISEYLTKTLPGWHVEDFLIDDASAGDVGACSDGLYQVGSVLSSQPSVRC
HANTAARF

>ZmBBX4

LCEVCEHAPAAVTCRADAAALCASCDADIHSANPLARRHERLHVAPFFGAL
ADAPKPFASAAPPKATDDDGSNEDEAASWLLPEPDHGQKEGATTEVFFADS
DPYLDLDFARSMDEIKTIGVQQSGSPELDLAGTKLFYSDHSVNHSTRIRLQVS
SSEAAVVPDAASGMAPMVAVVSRGLEREARLMRYREKRKSRRFEKTIRYAS
RKAYAETRPRIKGRFAKRTPGAGEDTLEEHEEMYSSAAAAVAALMAPGGAD
ADYGVVPTY

>ZmBBX5

MRIQCDACEAAAAAVVCCADEAALCARCDVEIHAANKLASKHQRLPLGDA
AALPASLPRCDVCQERPAFIFCVEDRALLCRDCDEPIHVPGTLSGNHQRYLAT
GIRVGFSSVCGASAEGLPPAPPKGSSKPTAVVSAPAAGTTTKTRTVKDALPQE
VPSSPFLPPSGWAVEDLLELSDYESSDKKDSSLGLKELEWFEDIDLFHAHSPA
KTAMAEFFASPQPVSNAGFYKANGARLFKKSRMEVPDDDDEDYFIVPDLG

>ZmBBX6

MRTICDVCESAPAVLFCAADEAALCRPCDEKVHMCNKLASRHVRVGLADP
NKLARCDICENSPAFFYCEIDGTSLCLSCDMTVHVGGKRTHGRYLLLRQRVE
FPGDKPGHMDDVPMEIKDPENQREQNTPKEQMANHHNVNDPVSDGNCDGQ
GNIDSKMIDLNMRPARTHGQGSNSQTQGVDLSVNNHDSPGVVPTSNSERDAI
K

>ZmBBX7

MSDADAAGSKEAPRCDYCMGLPAVVYCRADSARLCLPCDRHVHGANTVST
RHARAPLCARCRAAAATAVASPRGGGGGFVCADCYCLEKEDEEKGEEHRD
PRPLHHDHDAVEEYAGCPSIAELAAILGVAGYDEKAAAAGGDVGWWSTWE
EPQVVCLEDVVVPTTSCHGLHPLLAPPSPKNRSSGGELADVVIRQLEELAKSE
AAAVAASYAELEPGDGEQLPPWASPELDIGAAADFGALDAADADAAWHDA
ATMAFAAVPSHEEQEAWIATACDVDARRAEVEVEEAREQAAPAPDEPCLSS
FVDMSEIFPASVTLSSGGDVDNSGNKPDEETAPRPQLLATTALVPVTEKKGG
YDVAHPDRGTVISRYKEKRKNRRFGKQIRYESRKARADGRMRIKGRFAKSG
EV

>ZmBBX8

MKVLCSACEAAEARLLCCADEAALCARCDRDVHAANRLAGKHHRLPLLPP
DDVSAPNCDICQEAHAYFFCVEDRALLCRACDMAVHTANAFVSAHRRFLLT
GVQVGLQPAAAAQDADPHPPAAAEPLQTPPPPDRKAAAAGDGSSPALLYSD
DDIDWAAGADAGGGVSVAVTLPDWSLVHEQFGAPPVVPRHADPALARTPSS
KRSPRRSVAAAFTVQGGGGLAGGLPDWPLDEFFGFSEYSAGLAFAENDTSK
ADSGKLGSTDGSPAGRPSSDASQDFFGQVPEFHQWSVPELPSPPTASGLHWQ
GGPRHGAATTTDVAAVFVPDISSPENPFWCYATAAGQAPTKRRRRC

>ZmBBX9

MGGEGSTSPAPGGAACAVCGGAAVVYCVADAAALCSPCDAAVHAANLLAS
RHERVPLSMAAVPSASGAYDDLFAPDDVDAAAPAQAQGLGSPRNGSSSASF
TTSGDSGAEGSSLFDFDLLSGVDLAACVTDGVAPLHHDDAAPLWAQPGLAV
AWATAWSPADSAAAVVAAAAEREARVQRYREKRKNRRFHKTIRYASRKAY
AEARPRIKGRFVKRAAGTSSSSSSGAGTPDDGNDATGAAAKFWLSFSDDAR
DGFYVDAGAYGVVPSFYRVRSEQLTS

>ZmBBX10

MKIQCDACEGAAATVVCCADEAALCARCDVEIHAANKLASKHQRLPLEALS
ASLPRCDVCQEKAAFIFCVEDRALFCRDCDEPIHVPGTLSGNHQRYLATDIRV
GFASASSACSDACDAHDDSDHHAPPKAAVSSAAQQVPSPPQFLPQGWAVDE
LLQFSDCESSDKLHKESPLGFKELEWFTDIDLFHEQTPKAGRRLAEVPELSGT
QAANDAAYYRPAKATATAGAGVRQSKKARTEVTDDEDHLIVPDLG

>ZmBBX11

MELEQKPPPPPAGYWSGVAGGRPCDACAARPARLHCRADGAFLCPGCDAR
AHGAGSRHARAWLCEVCEHAPAAVTCRADAAALCAACDADIHSANSLARR
HERLPVAPLLGALSDAPAPQHFPSAAAAAGEEASAAEEEDGSDEAEAASWL
LPEPDNSHEDSAAADSFFAEPDAYLGVDLDFARCVDGVKAIGVPVTPAPLEL
DMAAGSFFYPQHSMNHSVPSSSEVAVSRGKEREARLMRYREKRKSRRFDKT
IRYASRKAYAETRPRVKGRFAKRCSEDDDALEHEEAACFSPAGSASASSDGV
VPSLC

>ZmBBX12

MKVQCDVCAAEAAEVFCCADEAALCDACDRRVHRANKLAGKHRRFSLLSP
APPPPPPLCDICQDKRGLLFCKEDRAILCRDCDVSVHTASDLTMRHARFLLTG
VRLSAEPAAACPAPEDEEEEDDENSSGSFCCSAGDAAAHPPPLPSSAPATSHG
SDSSSISEYLTKTLPGWHVEDFLVDEAAAAAATDIGVSAADASYQDGYPAW

MAAQERLLCEGGGGARGSRERWVPQMATYSGPGLAVAVAGTNKRSRATSA
ASSFPYW

>ZmBBX13

MVPLCGFCGKQRSMIYCRSDAASLCLSCDRSVHSANALSRRHRRTLLCDRC
GLQPASVRCLEDNTSLCQNCDWNGHDAASGASGHKRQAINCYSGCPSSAEL
SRIWSFIIDIPTVAAEPNCEDGLSMMTIDDSDVTNHHGASDDKRLLEIANTAL
MSDPPSPDKLKPLIGSSSGDGFDVLPLATDQPAGPVSATPKVPYARDDNKFN
DGMYEDLCVDDADLTFENYEELFGTSHIRIEELFDDAGIDSYFEMKETPPFDF
NEQPKIVQLQCSDVVPADCAMSNTGERADSSLCIPVRQVRSSISHPLSGLTGE
SSAGDHQDCGVSPILLMGEPPWYSPGPEGSLAGGSRDSALTRYKEKKKKRM
FDKKIRYASRKARADVRKRVKGRFIKAGEAYDYDPLSQTRSY

>ZmBBX14

MTSAGAATGAALGARTARSCDGCMRRRARWHCPADDAFLCQTCDVSVHS
ANPLARRHHRVRLPSASCSSPPRDPDAPTWLHGLKRRPRTPRSKPGGSKSNK
HEATPSFIAAAASSAAVPDLEAEESGSGILGGNDDHHGFLQDDDEDLLYRVP
VFDPMLAEFYNPVADEGEQKPACSLLMPSLAETSPEFASGGSAEADGLSVSF
HVPDMELASFAADMESLLMGVDDDGFDCLGFLDEEKPQVNADLDAIVAPAP
EPEDKKRKRPEMILKLNYEGVIASWVRDGGSPWFHGERPHLDCHELWSDDF
TTGSRELLGGAVTPVTGGEREARVSRYREKRRTRLFAKKIRYEVRKLNAEKR
PRMKGRFVKRATLPPLPRPPPPQQQQQQKQLPRAPPHVGMVLPPPPVSNGRL
WF

>ZmBBX15

MQMLCDVCAAAPAAVICCADEAALCSACDRRVHRANKLAHKHRRIPLAQP
SGDESDADAKPLCDVCKERRGLVFCVEDRAILCPDCDDPIHSANDLTAKHTR
FLLVGAKLSAALVDAQAPHSPDDDDNDCGRGNGAAAEPDAVPAVCAQGSC
AAKASSLESGGGGGGGSGSGSSISEYLTNICPGWRVDDLLFDDSAFSAASKA
DSCDDGHEQVPSLDADLFDVVAGAGWPGKRGSAWSGVGALGFDKVPASV
VVVPTAAKQQQGCVRERTWDSDSDSDVFAVPELPQPPQAKKARPAPAPAPT
FWCF

>ZmBBX16

MEALVGRYWGLGGRRCGACGGSPAAAHCRTCPGGAYLCGGCDAGHARAG
HERVWVCEVCECAPAAVTCRADAAALCAACDADIHDANPLARRHERVPVQ
PIGAAADPAAEKAALLFGLAAEGKDGGDAAKVVVDASKLDLLFADDVVDP
FLAQDFARFPHADSVVPNDGSNCGAAVDFDFGGGVAVAAKQPSYSSYTAAS
LGHSGSSSEVGLVPDVMCGRGGSVTGGVIELDFAQSKAAYLPYAATPTHSLS
SLDVGAVPERSDGVMAGRLATPTPATATESREARLMRYREKRKNRRFEKTI
RYASRKAYAESRPRIKGRFAKRADDNDADADLDEAAAPAPPARSQPQPQPQ
PSYRYVLDFAAGYGVVPTF

>ZmBBX17

MKIQCDACEGAAATVVCCADEAALCARCDVEIHAANKLASKHQRLPLEALS
AKLPRCDVCQEKAAFIFCVEDRALFCQDCDEPIHVPGTLSGNHQRYLATGIR
VGLASASACSDACDAHDSDHHAPPKATIEPPHAAVSAAVQQVPSPPQFLPQG

WAVDELLQFSDYESSDKLHKEPTLGFKELEWFADIDLFHEQAPKASRTLAEV
PELFGYQAANDAAYYRPAKAAAGGGAGVRQSKKARIEVTDDEDYLIVPDLG

>ZmBBX18

MASLCDFCGKQRSMIYCRSDAASLCLSCDRNVHSANALSRRHTRTLLCDRC
GSQPASVRCLEDNASLCQNCDWNGHDAESGASGHKRQAINCYSGCPSSAEL
SRIWSFITDIPTVAAEPDYEDGLSMMTIDGSDVTNRHDTSDDKRLLEIANTTL
MSDPPSADKLKSPTGSSSGDGFDVMTLATDQPAGPVSATPKVPNARDDDKF
NDGMYEDLCVDDADLTFEDYEELFGTSHIRTEELFDDAGIDGYFELKETPPF
YFNEQPKAMQIECGNVVSADCAMSNPGARADSSLCIPVRQVRSSISHSLSGLT
GESSAGDHHDCGVSPMLLMGEPPWHSPGGPEGSVAGGSRDSALTRYKEKK
KRRKFDKKIRYASRKARADVRKRVKGRFIKAGEAYDYDPLSQTRSY

>ZmBBX19

MSSSKHAAGAVGGKAARACDSCLRRRARWYCAADDAFLCQGCDASVHSA
NPLARRHERLRLRPTDPHSTTLEAGVATATWKKRQQQVAPAWSKRKARTR
RPHVKSVGELLSRKLVVVPEVSPIESSEERKAEEEEEEEEGQLLYCVPTFDRA
LAELCSPPPPVDDPTASSCCRDDVDGAVENNTKAPPVVVAESPVQQLPDSFA
GFGPTDAELREFAADMEALLGQGLDDGNELDRSFYMESLGLMAQQAEDVG
RIKMEPNGIVSSRSRGEGAPGFGPTEMKPEASSAAAEVLDTDFNCCSPTVMM
DNEDEDSFEQKASASNAAAAAAGTQFLKRSLDLSLNYEAIIESWGSSPWTDG
QRPSVQLDDFWPHAHLTGWMAGGGRLGGEAAVTPRLGMGGGREARVTRY
REKRRTRLFAKKIRYEVRKLNAEKRPRMKGRFVKRPAAAAGGGGEELPLPP
RAPSPSLPIEVARVAAQSPCGVPIGVRPRPCPRECRGR

>ZmBBX20

MRIQCNACGAAEARVLCCADEAALCVACDEEVHAANKLAGKHQRVQLLTD
SATAAASPAPAVPKCDICQEASGYFFCLEDRALLCRDCDVAIHTVNSFVSVH
QRFLLTGVQVGLDPADPVPPIAEKHVNASGGSVKQSVRHLPRRSPGVQFSVE
GSASVPSKNVSNGDYSRQNSVPTARAEVVDWTKNNTTIQSVESPPKYMSEES
PTLLQSSQTTTVFSNQINGNSDGTYHLSFSSGNVTDSLPDWPVDEFFSNSEYV
PNLGFSEHGSSKGDNAKLESAGGSLQCRLAEGSIAEELLGQVPGLITDDYMS
RVPENSWTVPEVPSPPTASGLNWHGNLCFPAYDSTMFVPEITSLQNSQNQFT
VPSSFKRRRREY

>ZmBBX21

MQVLCDVCGGAPAAVLFCTDEAALCSACDRRVHRADKRRRIPLVQPCGDDS
AAAAAAPLCDVCKERRGLVFCVEDRAILCPDCDDPIHSANDLTAKHTRFLLV
GAKLSAALVDQAPPSPDDDDDDVAEPDAVPAACAAKASSLESGGGSSISDYL
TNICPGWRVDDLLLDDSSFSAPSKTGYSDGHDQVPSVDADLFDVVASGRPG
KRVGGAALGFDKAPASVVVVPTQGCVTERTWNSDSDVFAVPELPQPPPAKK
ARPAPAPTFWCF

>ZmBBX22

MIATTRGSSAKAAAAVGGKAARACDGCLRRRARWYCAADDAFLCQGCDTS
VHSANPLARRHERLRLCPASPLQTPPDRSAAAAATATNKRERHDEVAVPAW
FGRKARTPRGGHAKSVGQVALSRRLVVPHAAGGDSDSPDERNGGEEEQLLY
RVPILDPALAEFCSPPPLEDAAGLALDASVCNEDGAIEDPAKPDPAAPLAQFC
PVSGHFNFGPTDAELREFAADMEALLGHGLDDGNEEDSSFYMETLGLLDPM
EVGDDATQVKVETDGSSACCEASGTLACGLELDLEASDEMLDIDFDYASPQ

DTATDERAASSDTGADAQFLQTSLSLTLNYEAIIQSWGSSPWTGGGERPHVK
LDTRTCGLWEEWSATAARTCRARLGWGWTAAGRPGCRGTGRSGGRGSSPR
RYGTRCASSTPRSGHG

>ZmBBX23

MKVLCSACEAAEASVLCCADDAALCARCDREVHAANRLAGKHQRLPLLAP
GGQGAAAVSPPKCDICQECDAYFFCLEDRALLCRSCDVAVHTANSFVSAHR
RFLLTGVQVGQELEPDDLSLEQREASSPPPAKSEPTPPLYSESDFGWAAGAG
ATGSLTDWSAVQEEFGSPAPRLAEAAPRATPKRTPRAPAFGAGQGRIAGGV
MDWPLGEFFRGVSDFNGGFSFGESGNSKADSGKLGDSAGGSPYYRSSEEER
DANELFGQVPEIQWSVPALPSPPTASGLHWQHGGPDSGAFVPDICTPDGAGR
CFPTASGAAKRQRNR

>ZmBBX24

MRTICDVCESAPAVLFCAADEAALCRPCDEKVHMCNKLASRHVRVGLADP
NKLVRCDICENSPAFFYCEIDGTSLCLSCDMTVHVGGKRTHGRYLLLRQRVE
FPGDKPGHMDDVPMEIQDPENQRDQKKPPKEQTANHHNGDDPATDGNCDD
QGNIDSKMIDLNMRPVRTHGQESNSQVCHAHCD

>ZmBBX25

MKIQCNACGAAEARVLCCADEAALCVACDEEVHAANKLAGKHQRVPLLTD
AAAAAAAAAPAVPKCDICQEASGYFFCLEDRALLCRDCDVAIHTVNSFVSV
HQRFLLTGVQVGLDPADPVPPIAEKHVNAVGGSVNQPVRHQPRRSPTVQFSV
EGSASVPTKNVTNGDCSRQNFVPTARAEVVDWTMNNSTIRSVESPPKYISEE
SPTLLQSSQTTTVFSNQINGNSDGAYHLSFSGGNVTDSLPDWPVDEFFSNSEY
GPNFGFSENGSSKGDTAKLGGAGGSPQCRLAEGSVAEELLGQVPGLITDEYM
GRVPENSWTVPEVPSPPTASGLNWHGNLCFPAYDSTMFVPEITSLQNSQSHF
TVPSSFKRRRREY

>ZmBBX26

MIATTTTGSSAKAAAVGGKEARACDACLRRRARWYCAADDAFLCQVCDTS
VHSANSLARRHERLRLRPTSPLQTPPPPTPASANRESHDEVVPAWFKRKART
PRGGRAKSDVRTLSRRLVVPHAAGGDSPDGRNDEGEFEAEEPEEEVLYRVP
VFDPALAEFCSPQPLEDAAALASSCNEDGAVEDPAKTDRETPAAAPLVQFFP
DGGHANFGPTDAELREFAADMEALLGYGLDDGNEESSSFCMETLGLLEPVE
VGEDASRVKVETDAGSACEASGTLACALELLDPDASDEMLDIDFNYGSPQD
TTTTENAASSSHTGTDGQFLQTSLSLTLNYEAIIQSWGSSPWTGGAERPHVKL
DDSWPHDCTNMWVVGRGMVGHGGEDLLGTPRLGQGMDDVGREARVSRY
REKRRTRLFSKKIRYEVRKLNAEKRPRMKGRFVKRATAGGSLTIAGLA

>ZmBBX27

MDYNFDTSVLDEDVAGRGGREGSCPPAWARACDGCRAAPSVVYCHADTA
YLCASCNSRVHAANRVASRHERVRVCEACECAPAVLACRADAAALCAACD
AQVHSANPLAGRHQRVPVLPLPAAAVPAASVLAEAAATAAAVAGDKDEEV
DSWLLLTKDPDDDDKNHNCSSNNNNISSNTSTFYADVDEYFDLVGYSSYCD
NHINSNTKQYGMQEQQLLLHKEFGDKEGSEYVVPSQVGQQQSGYHRVIGTE
QAASMTPGISFSSSMEVGIVPDNMATTDMPSSGILLTPAGAISLFSSGPPLQMP

LHLASMDREARVLRYREKKKSRKFEKTIRYATRKTYAEARPRIKGRFAKRSS
DMDVEVDQMFSAAALSSDGSYGTVPWF

>ZmBBX28

MGALCDFCGEHRSMVYCRSDAASLCLSCDRNVHSANALSRRHTRTLLCDRC
ASQPAMVRCLAENASLCQNCDWNGHIAGSSSAGHKRQTINCYSGCPSSAELS
RIWSFVSDIPNVAPEPNCEQGISMMSISDSGVSNQDNAAGDSILLDIASATLVS
DIGTCDKLLVGSSSGAGVNLLPLATGQTETAGSVDSTPDKDSCMQVPYTPDK
DMFSKDSIYEDFCVDDADLAFENYEELFGTSHIQTEQLFDDAGIDIYFEMKEA
PAGNSTEQSKLKQPANSNAVSADSGMSNPGVKGDSSVCTPLRQARSSLSLSF
AGLTGESSAGDHQDCVVSSLLLMGEPPWQPPGPEGSIAGGSRDSALTRYKEK
KMRRKFDKKIRYASRKARADVRKRVKGRFVKAGEAYDYDPLCQTRSY

>ZmBBX29

MKGDEKSAGGAPAYWGLGARPCDACGAEAARLYCRADAAFLCAGCDARA
HGAGSRHARVWLCEVCEHAPAAVTCRADAAALCASCDADIHSANPLASRH
ERLPVAPFFGELADAPKPFASSAAVPKAADDDGSNEAEAASWLLPEPDHGQ
KEGATTEVFFADSDPYLDLDFARSMDDIKTIGVQGGPPELDLAGAKLFYSDD
SMNHSVSSSEAAVVPDAVAGAAPEVAVVCRGLEREARLMRYREKRKSRRF
DKTIRYASRKAYAETRPRIKGRFAKRTPGAGADGEEPLEEHEEIYSSAAAAV
AALMAPGGADADYGVVPTH

>ZmBBX30

MKVQCDVCAAEAASVFCCADEAALCDACDDRVHRANKLAGKHRRFSLLHP
CSSSAQKPPLCDICQERRGFLFCKEDRAILCRECDAPVHSANDMTRRHSRFLL
TGVRLSSAPVDSADPSEGEEEEEQENSSRPGNGESCSGGAGATTATASDGSSI
SEYLTKTLPGWHVEDFLVDDAYASDVGACSSDGLYQGQQDGQISGVLQEA
YMPWTGRELVPADVADERANWERWVPQMHAEFAGDSKRPRASPSPPCSY
W

>ZmBBX31

MGHHRGGCRCELCGAPAAVHCAADAAFLCAACDAKVHGANFLASRHRRT
RLLLAAPDECGYESGASSCVSTAVDSAAPPPRRTRGGGSPGPRSRGEAVLEA
WARRTGLTAADARRRAAAAARALRAHAHGLASARVAPRVAMAAALWRE
VAGRGGTGGGGHGHGEALRRLEACAHVPAGLVVAVAKSMARARGRGDEA
DTDAAAEGWDECAWAGPKSSPPRP

Table S4

Type	Domain organization (N-terminus to C-terminus)	Number
I	B-box1 + B-box2 + CCT	3
II	B-box1 + B-box2 + CCT	6
III	B-box1 + CCT	2
IV	B-box1 + B-box2	7
V	B-box1 only	14

Table S5

Name	Amino acid sequences
RcPAL1	MESITQNGHHHQNGIQNGSLDDGLCIKTESIKTGYSVSDPLNWGAAAE<br>SMTGSHLDEVKRMVTEYRKSVVKLGGETLTISQVAAIANHDSGVKVE<br>LAESARAGVKASSDWVMDSMNKGTDSYGVTTGFGATSHRRTKQGAA<br>LQKELIRFLNAGVFGNGTESAHTLPHSATRAAMLVRINTLLQGYSGIRF<br>EILEAISKFLNHNITPCLPLRGTITASGDLVPLSYIAGLLTGRPNSKAIGPK<br>GETLTAAEAFAQVGISSGFFELQPKEGLALVNGTAVGSGLASMVLFET<br>NILALLSEILSAIFAEVMQGKPEFTDHLTHKLKHHPGQIEAAAIMEHILD<br>GSSYVKAAKKLHEQDPLQKPKQDRYALRTSPQWLGPQIEVIRFSTKSIE<br>REINSVNDNPLIDVSRNKALHGGNFQGTPIGVSMDNTRLAIASIGKLMF<br>AQFSELVNDFYNNGLPSNLSGGRNPSLDYGFKGAEIAMASYCSELQFL<br>ANPVTNHVQSAEQHNQDVNSLGLISSRKTAEAVDILKLMSSTFLVALC<br>QAIDLRHLEENLKSTVKNTVSQLAKRVLTTGVNGELHPSRFCEKDLLM<br>VVEREYLFTYIDDPCSATYPLMQRLRQVLVEHALTNGENEKNANTSIF<br>QKITAFEEELKTILPKEVESTRAAYESGNAAIPNRIVECRSYPLYKFVRE<br>ELGGEFLTGEKVRSPGEECDKVFTAMCQGNIIDPILDCLSGWNGEPLPI<br>C
RcPAL2	MESFKNCNAAVESFCEGHDPLNWNMAAESLKGSHVDELKRMVSDYR<br>KPVVKLGGETLTIGQVAAIASHDGGVRVELAEEKRAGVKASSDWVMD<br>SMGKGTDSYGVTTGFGATSHRRTKNGGALQRELIRFLNAGIFGSSLDST<br>HILPHTATRAAMLVRINTLLQGYSGIRFEILEAITKFLNGNITPCLPLRGT<br>ITASGDLVPLSYIAGLLIGRPNSKSVGPKGETLSPAEAFKLAGIEGGFFEL<br>QPKEGLALVNGTAVGSGMASMVLFDVNTLAVLSEIMSAIFAEVMQGK<br>PEFTDHLTHKLKHHPGQIEAAAIMEHILDGSSYVKEAKKVHEMDPLQK<br>PKQDRYALRTSPQWLGPQIEVIRAATKMIEREINSVNDNPLIDVSRNKA<br>LHGGNFQGTPIGVAMDNTRLAIASIGKLMFAQFSELVNDYYNNGLPSN<br>LSGGSNPSLDYGFKGAEIAMASYCSELQFLANPVTNHVQSAEQHNQDV<br>NSLGLISSRKTSEAVDILKLMSSTFLVALCQAIDLRHLEENLKIVVKTTV<br>SNVAKRTLTVSPNGELHPSRFSEKDLLTVVDREYLFSYIDDPCLATYPL<br>MQKLRAELVEHALKNGERERSANTSIFHKISAFEEELKTILPKEVDNARI<br>EIENGKSEIPNRIKECRSYPLYRFVREELGTSLLTGEKIKSPGEECDKLFN<br>AICAGKLIDPLLECLKEWNGAPLPIS
RcC4H	MDFLLLEKTLLGLFVAVVVAITVSKLRGKKFKLPPGPIPVPVFGNWLQ<br>VGDDLNHRNLTDMAKKFGDVFMLRMGQRNLVVVSSPELAKEVLHTQ<br>GVEFGSRTRNVVFDIFTGKGQDMVFTVYGEHWRKMRRIMTVPFFTNK<br>VVQQYRYGWESEAAAVVEDVKKHPEAATNGMVLRRRLQLMMYNN<br>MYRIMFDRRFERSERRESRLAQSFEYNYGDFIPVLRPFLRSYLKICKEV<br>KEKRIQLFKDYFVDERKKLASTQATTNEGLKCAIDHILDAQQKGEINED<br>NVLYIVENINVAAIETTLWSIEWGIAELVNHPEIQKKLRVELDTVLGRG<br>VQITEPEIQKLPYLQAVVKETLRLRMAIPLLVPHMNLNEAKLGGFDIPA<br>ESKILVNAWWLANNPAHWKKPEEFRPERFLEEESKVEANGNDFRYLPF<br>GVGRRSCPGIILALPILGITLGRLVQNFELLPPPGQTQLDTTEKGGQFSL<br>HILKHSTIVMKPRT
Rc4CL1	MAHNSATPTPATVDPRSGFCKSNSIFYSKRKTEPLPPNDSLDVTTFISSQ<br>AHRGNIAFIDAATGRHLTYADLWRAVYSVASSLSDMGIRKGHVILLLS<br>PNSIFFPVVCLAVMSLGAIITTTNPLNTTREISKQVGDSKPVLAFTTRQL

LPKLAGSPLSSNSIVVIDDDDVGQVPNNNSSKNIFALTLGAMMKRQPT
GGRFREAINQDDTATLLYSSGTTGASKGVVSSHRNLIATVRTVLGRFVS
QVAEEPQTFLCTVPMFHIYGLATFATGFLTSGSTIVVLSKFEMHDMLSA
IGRYKVTYLPLVPPILVALANGADQIKAKYDLSSLHSVLSGGAPLSKEV
IEGFLEKYPTVGISQGYGLTESTGLGASTDTLEESRRYGTAGMLSPNME
AKIVDPDTGRALTVNQTGELWLKGPTIMKGYFCNEEASASTVDSQGW
LRTGDLCYIDDDGFIFVVDRLKELIKYKAFQVPPAELEALLLTHPQIAD
AAVIPFPDEKVGQFPMAYVVRKAGSNLSETAVMDFVAKQVAPYKRIR
KVAFIASVPKNPSGKILRKDLIQLATTSTSKL

Rc4CL2 MEKSGYGRDGIFRSLRPPLVLPRDPNLTMVSFLFRNSSSYPHKPALIDD
DSSETLSFSQLKLMVIKVAHGLLHLGIKKNDVVLIFAPNSIQFPICFLGII
AIGAIATTSNPLYTVSELSKQVKDSNPKLVITVPELWEKVKGFNLPAVIL
GQKTSSSQTESSSRIVGFHDLVESSGPVSDFPSVNVKQTDTAALLYSSG
TTGMSKGVILTHRNFIASSLMITMDQELAGEMHHVFLCVLPMFHVFGL
AVITYSQLRKGNAVISMARFNLEKILMAVEKYKVTHLWVVPPIILALSK
DSVVKKYNLSSLKHIGSGAAPLGKELMEECAKIIPQGVVSQGYGMTET
CAIVSVENTLVGPRHSGSAGSLASGVESQIVSVDTLKPLPPKQLGEIWV
RGPNMMIGYFNNPEATKLTLDKNGWIHTGDLGYFDESGQLFVVDRIKE
LIKYKGFQVAPAELEGLLVSHPEILDAVVIPFPDAEAGEVPVAYVVRSP
NSSLTEEDIKSFIASQVASFKRLRRVTFINTVPKSASGKILRRELIEKVRS
KI

Rc4CL3 MADHTGSSCIDPYSGFCSKTKTFHSLRPKAPLPPETTPLSITHFIFSQLQA
SPPPPSTPALIDPATRHCILYPDFTRRVQSLAAALQSQLNLSNGHTAFVL
SPNSLHLPILFFSLFSLGVTVSPSNPASTNPEISRQIHLSKPVVAFATSATA
HKIPNSLRYGTVLLDSAEFESMMTCPGRTPDLPRARVYQSDTAAILYSS
GTTGMVKGVALTHRNWISMLAAAFAVRPSSPPAVWLCTVPFFHVYGF
GVCMRVLAFGETLVSISGRFDLRSTLSAIEEFRITHAAWAPPVVVALVK
LGSELDGYDLSSLQVIASGGAPLAKSVIDKLKKRLPNVQLAQGFGLTET
SGRVFGTVGPNETRVEGAVGKLMSNFEAKIIDPETGIALPPLMPGELWL
TGPFLMKGYIGDEDATASTMDSQGWFKTGDLCYIDEQGYLFFVDRIKE
LIKYKGYQVAPAELEHLLLSHPDIVDAAVVPYPDEEVGQVPMAFVVRC
VGSALDESQIKDFIAKQVAPYKKIRRVTFINEVPKSAQGKVMRKELIKL
ASSKL

Rc4CL4 MENKRQDNHEFIFRSKLPDIYIPNHLPLHTYCFENLSQFHDRPCLINGNT
GETFTYAEVELTSRRVAAGLDKLGIQQNDVVMLLLQNCPEFAFAFLGA
SYIGAMSTTANPFYTPAEVAKQAKASNAKLIITQSPYVDKVKDFAKLN
DVKVMCVDETLSEDVLHFSELTSADESETPAVKINPDDVVALPYSSGT
TGLPKAVAQQVEGENPNLYFHKEDVILCVLPLFHIYSLNSVFLCGLRVG
AAILLMQKFEINKLLELVEKEKVTIAPFVPPIVLSIAKCPDLHRYDLSSIR
MVMSGAAPMGKELEDTVRAKLPNAKLGQGYGMTEAGPVLSMCLAFA
KEPYEIKSGACGTVVRNAEMKIIDPDTNESLPRNQSGEICIRGSQIMKGY
LNDPEATENTIDKEGWLHTGDIGFPSRPAELEAMLISHPNLSDAAVVSM
KDEAAGEVPVAFVVRSNGSKISEDDIKQYISKQVVFYKRISRVFFTDKIP
KAPSGKILRKDLRTRLAAGLPN

Rc4CL5 MLTHKGLVTSVAQQVDGENPNLYFHKEDVILCVLPLFHIYSLNSVFLC
GLRVGAAILLMQKFEINKLLELVEKEKVTIAPFVPPIVLSIAKCPDLHRY
DLSSIRMVMSGAAPMGKELEDTVRAKLPNAKLGQGYGMTEAGPVLS
MCLAFAKEPYEIKSGACGTVVRNAEMKIIDPDTNESLPRNQSGEICIRGS

	QIMKGYLNDPEATENTIDKEGWLHTGDIGYIDDDDELFIVDRLKELIKY KGFQVAPAELEAMLISHPNLSDAAVVSMKDEAAGEVPVAFVVRSNGS KISEDDIKQYISKQVVFYKRISRVFFTDKIPKAPSGKILRKDLRTRLAAG LPN
Rc4CL6	MENKRQDNHEFIFRSKLPDIYIPNHLPLHTYCFENLSQFHDRPCLINGNT GETFTYAEVELTSRRVAAGLDKLGIQQNDVVMLLLQNCPEFAFAFLGA SYIGAMSTTANPFYTPAEVAKQAKASNAKLIITQSPYVDKVKDFAKLN DVKVMCVDETLSEDVLHF
Rc4CL7	MARTSSYNFNSQIPETPNIEKHKSLATQQHPWWFSPDTGIYHSKQPSIN LPTDPFLDVVSFVFSHKHNGVSALVDSSSGFSISYSKLYSLVKSMASGL HRMGISQGDVVLLLLPNSIYYPIVFFGVLYVGAVVTTMNPLSSVVELK KQIADCNACLAFTGSENVDKLQALGVPAIGVPENVVSDSTKEFFSVFHE LVYGKFSLAPRPVIKLQDTAAILYSSGTTGVSKGVLVTHGNFIATVELF VRFEASQYEYSSLNNVYLAVLPLFHIYGLSLFVVGLLSLGSRIVVMKKF DVNEMVKAIDRYKVTHFPVVPPILTALTKIAKDVGAHSLQSLKQVSCG AAPLSMKSIEDFVQTLPYVDFIQGYGMTETTAVGTRGFNTEKVRKYSSI GLLAPNMQAKVVDWNTGSFLPPARIGELWLRGPSIMRGYLNNARETM STIDDDGWLHTGDIVYFDEDGYLNICDRIKEIIKYKGFQIAPADLEAVLI SHPEILDVAVTGATDEECGEIPVAFVVRKHSSELSQQDVMDYVARQVS PTRRSERWCLQTQYQDLQQGRSSEGSSGFSWLLDCAWDFASYCFSRD RSCNRNFLAASFVAAAIL
Rc4CL8	MAEHTDNPSRWIDPKSGFCPRTKTFHNFRPPVPLPPLSQPLSLAQYTLSL LQSSTTAPTTPVLIDATSGRHVSYGQFLAQFHSLTRSLRSLISKGQVAFI LSPPSLHVPVLYFSLLALGVVVSPANPIGSESEVAYQVRLCKPAVAFAT SATAHKLPKGLTTILLDSPEFLSLIDGSRPGTRPDYPVEVNQTDSAAILY SSGTTGRVKGVISTHRNFIALIAGMHANRLEPDPNEPVVQAVSMFTLPL FHVFGFFMLIRAVSMGETMVLMERFNFEAMLRAVERFKVTYMPVSPP VIVALANSELAQKYDLSSLRLLGCGGAPLGKEVAERFNRKFPNVEIVQ GYGLTETGGGATRMIDPEEAKNYASVGRLAENMEAKIVDPETGEALPP GQRGELWLRGPTVMKGYVGDDKATAETLDKDGWLKTGDLCYFDDE GFIYIVDRLKELIKYKAYQVPPAELEQILLSHPDIADAAVIPYPDEEAGQI PMAYVVRRPGNNITEAIVMDFVAKQVAPYKKIRRVSFINSIPKSPAGKI LRRELVTLALSSGSSKL
Rc4CL9	MAAQTPQHDIVYRSKLPDIHIPNHLPLHSYIFQNKSHLSSKPCIINGTTG DIHSYADVELTSRKVASGFNKLGIKQGDVIMLLLPNTPEFVFAFLGASF CGAMTTAANPFFTPAEIAKQAKASKAKLIITLACYYDKVKDLSCDEVK LMCIDSPPPDSSCLHFSELTQSDENDVPDVDISPNDVVALPYSSGTTGLP KGVMLTHKGLVTSVSQQVDGENPNLYYSSDDVVLCVLPLFHIYSLNSV LLCGLRAGAAILVMQKFEIVSLLELMQKHRVSVAPIVPPIVLAIAKFPDL DKYDLGSIRVLKSGGAPLGKELEDTVRAKFPNVTLGQGYGMTEAGPV LTMSLAFAKEPFEVKPGGVGLSSETQS
Rc4CL10	MKGYLNDPEATRTTIDKQGWLHTGDIGFVDDDEELFIVDRLKELIKYK GFQVAPAELEALLVTHPNISDAAVVPMKDDAAGEVPVAFVVRSKGSQI TEDEIKQFISKQVVFYKRINRVFFIEAIPKSPSGKILRKELRTKLAAGFAN

Rc4CL11	MHKVGYTLEILDILMKRGDCMLWIDLKSSSSVMVIRFPDVKAGEVPIA CVVLAPNSLLTEQDILKFVEKQVAPLHHAKDYGA
Rc4CL12	MISIASNNNHNNNNNRVVVETPTKPEISPNIISDVISTSQTQPEQEQQPPT TTNHHVFKSKLHRKILHLLRNPLFFPKDRSGPIQARHPQRRCGHDPSPE LRGVRLLIHGRFHDRRRHDHRQPFLHRLRNLQAVGGIQRQAHHHTIPV RRQAPTARPALSSGHHRRPPENCLHFSVLSDANENELPQVSIDPDDPVA LPFSSGTTGLPKGVILTHKSLITSVAQQVDGENPNLYLKGDDVVLCVLP LFHIFSLNSVLLCSLRAGAAVLVMLKFEIATLLELIQRYRVSVAAVVPPL VIALTKNPMVADYDLSSIRVVLSGAAPLGKELEEALRSRVPQAVLGQG YGMTEAGPVLSMCLAFAKQPFPTKSGSCGSVVRNAELKVVEPETGRSL GYNQPGEICIRGSQIMKGYLNDSEATATTVDVEGWLHTGDVGYVDDD DEVFIVDRVKELIKFKGFQVPPAELESLLVSHQSIADAAVVPQKDDAAG EVPVAFVVRSNGGNELTEEAVKEFIAKQVVFYKRLHKVYFVHAIPKSP AGKILRKDLRAKLAAAATPNPRHPI
RcCHS1	MVTVDEVRKAQRAEGPATVLAIGTATPPNCVDQSTYPDYYFRITKSEH KTELKEKFQRMCDKSMIKKRYMYLTEEILKENPSMCEYMAPSLDARQ DMVVVEIPKLGKEAATKAIKEWGQPKSKITHLVFCTTSGVDMPGADY QLTKLLGLRPSVKRLMMYQQGCFAGGTVLRLAKDLAENNRGARVLV VCSEITAVTFRGPSDTHLDSLVGQALFGDGAAAIIVGADPLPEIERPLFE LVSAAQTILPDSDGAIDGHLREVGLTFHLLKDVPGLISKNIEKSLNEAFK PLDITDWNSLFWIAHPGGPAILDQVEAKLGLKPEKLEATRNILSEYGNM SSACVLFILDEVRRKSAANGHKTTGEGLEWGVLFGFGPGLTVETVVLH SVAAST
RcCHS2	MVTVEEVRKAQRAEGPATVLAIGTATPPNCVDQSTYPDYYFRITNSEH KTELKEKFQRMCDKSMIKKRYMYLTEEILKENPSMCEYMAPSLDARQ DMVVVEIPKLGKEAATKAIKEWGQPKSKITHLVFCTTSGVDMPGADY QLTKLLGLRPSVKRLMMYQQGCFAGGTVLRLAKDLAENNKGARVLV VCSEITAVTFRGPSDTHLDSLVGQALFGDGAAAIIVGADPLPEIERPLFE LDVPGLISKNIEKSLNEAFKPLDITDWNSLFWIAHPGGPAILDQVEAKL GLKPEKLEATRNILSEYGNMSSACVLFILDEVRRKSAANGLKTTGEGLE WGVLFGFGPGLTVETVVLHSVGVTA
RcCHI1	MMVSFRFPFSFSQPPPATCSSATSRPFSAVTAAVTVAAGATAFAGVAA VSQTNPTNQTHPFLQNALNFFFANRSLPLWGSLSLNDTPASVVDSKTG VAFPSVLAESRRLLGIGLRRKRVLGLKNIDVYAYGIYADGNDVKKLLS EKYGKLSLSELQENKEYNDVLLETDIGMTVRLQIVYGKLSIGSVRSAFE ESVGTRLQKFGGSDNQELLQRFTSQFKDEIKIPRGSIIDLSREPGYVLRT TIDGNDVGSIQSKLLCKSILDLYIGEEPFDKQAKEDVKLNLASVLQE
RcCHI2	MAPPITGIQIEATSFPPTVKPPGSGNSLFLGGAGVRGLEIQGNFVKFTAIG VYLEDKAVPELAVKWKGKTAEELTESVQFFREIVTGPFEKFTQVTMILP LTGQQYSEKVSENCVAIWKKFGIYSDAEAKAIEKFTEVFRDQTFPPGAS ILFTQSPNGSLTIGFSKDGSIPEVGNAVIENKLLSEAVLESIIGKQGVSPA ARQSVAARLSELLKESDDSVTGNGKVEKCTKEAEVEA
RcCHI3	MQPWDPLLQNTAMGAVGTEAVLVDEIPFPSVITTTKPLSLLGHGITDIEI HFLQIKFTAIGVYLDPEIVGHLQQWKAKKGTELAEDDGFFDALISAPVE KFIRVVVIKEIKGSQYGVQLESSVRDRLAADDKYEEEEEEALEKIVEFF

QSKYFKKDSIITFHFPATSHTAEIVFTAEGKESSKIKVENANVVETIKKW
YLGGTRGVSPSTISSLANTLSAELTK

RcF3H1 MEVVRVQTLALGGLNELPAKFIRPAHEQPKNSKALEGVSVPMISLAQP
HDVVVKEVVKAAAEWGFFLLTDHGIPAFLIQELQKVGNEFFMLPQEEK
EAYANDPASGKFDGYGTKMTKNHDEKNPPSYREVNEKYNNEMLRVT
DKLLELLSEGLGLDKKVLKSHVGGEQVELEMKINMYPPCPQPQLALG
VEAHTDMSALTLLVSNDVPGLQLWKDDNWVAVNCLPNAVFVHIGDQ
IEVLSNGKFKSILHRSLVNKEHLRMSWAMFIVPPHEAVIGPLPELVNNQ
NPAKYSTKTYAEYRYRKFNSIPQ

RcF3H2 MEAGASTTASSSFTSALTLTQLGVSLVPQRYVLPPSHRSSPSYPNLSTTA
PLPILDLSSLQSPSLRPHVINDIHTACKEIGFFQVINHGIPLSILKDALSAA
NEFFNLPIEEKMVLGSDNVHAPVRYGTSMNQAVDRVHFWRDFIKHYS
HPISKWIHLWPSNPSSYKEKMGNYAKAVQTLQQQVMELVIESLGLNPN
YLDEEVENGSQVMAVNCYPKCPEPELALGMPPHSDYGFITILLQSCPGL
QIMDQNNNWVSVPDTEGALLVQMGDQMEVLSNGQYKSVVHRVTVS
DDKNRLSIASLHSLGLDKKIAPAPLLVDNEHPKSYREFSFRDFLDYITSN
DIIKGIRFIDTLKENP

RcFLS1 MEVVRVQTLALGGLNELPAKFIRPAHEQPKNSKALEGVSVPMISLAQP
HDVVVKEVVKAAAEWGFFLLTDHGIPAFLIQELQKVGNEFFMLPQEEK
EAYANDPASGKFDGYGTKMTKNHDEKNPPSYREVNEKYNNEMLRVT
DKLLELLSEGLGLDKKVLKSHVGGEQVELEMKINMYPPCPQPQLALG
VEAHTDMSALTLLVSNDVPGLQLWKDDNWVAVNCLPNAVFVHIGDQ
IEVLSNGKFKSILHRSLVNKEHLRMSWAMFIVPPHEAVIGPLPELVNNQ
NPAKYSTKTYAEYRYRKFNSIPQ

RcFLS2 MGVERVQDIASSTLKDTIPAEFIRSENEQPGITTVPGTVLECPTIDFSDPD
EEKLLAQILAASTDWGMYQIVNHDISNEAIAKLQAVGKEFFELPQEEKE
VYAKDPNSKSVEGYGTFLQKELEGKKGWVDHLFHRIWPLSAINYRFW
PKNPASYREANEDYAKNLHKVVEKLFKLLSLGLGLEAQDLKKAVGGD
DLVYLLKINYYPPCPRPDLALGVVAHTDMSALTILVPNNVQGLQACRD
GQWYDVKYIPNALVIHIGDQMEIMSNGKYKAVLHRTTVSKDQTRISW
PVFLEPPQDLEIGPHPKLVDDKENPPKYKTKKYREYVYNKLNKIPQ

RcFLS3 MKIYTDFGEKIRKNQSEAKLLFEGDELLNISPEKGDRGGDLEMWLSFLT
LLLSNRFLVINHGIPLSILKDALSVANEFFNLPIEEKMLLGSDNVYAPVR
YWTSINQVVDSVHFWRDFIKHYSHPISNWIHLWPSNPPSYKEKMGNYA
KAVQALHQQIVELVIESLGLNPNYIHEEVENGSQVMAANFFPKCPEPKL
ALGMPPHSDHGFITILLQSCPGLQIMDQNKNWVSVPDIEGALLVQMGD
QMEVLSNGHYKSVVHRVTVSNECNRLSIASVHNLGLDKKIEPAPLLVD
NEHPKSFKEFSFRDFLDYITSNDNSKGRFIDTLKENPRTDT

RcF3′H1 MEDPLWYSLALIIFILVVIKLFITNTSHKYKNLPPSPPCVPIIGHLHLLRQP
IHRTLESLSANFGKIQLLRWGSRRVLLVSSPSIAEECFTKHDIAFSNRPLL
LAGKHFHYNYTTVVVAPYGDLWRNLRRIMTLEIFSSSRVAISSSIRREQ
VRLLLDQIIKSCNSGTPKVELKSKFMDLAFNVMTMMILGKRYYGEDV
GDDEEASKFREAVRDAVELNASTNLGDFLPFFQWIDVFGTEKKMVRL
MAKMDSFLQAMVSDRRQLLSSNCDQNNTGEVSKLLVDNLLFLQQKEP
ELYTDEIIKGIIVVLLVAGTETVSTTMEWAMSLLLNHPDKLDKVKAEIE
NKVGQERLLDEQDLPNLNYLQNVINETLRLYPPIPLLGAREASEDCVVS

	GFDVPCGTMMLVNAWAIHRNSELWDEPTTFQPERFEGWSGEGPGGYR<br>LIPFGGGRRGCPGAGLANRLIGLVLGSLIQSFEWERISEDKVDMSEGIGL<br>TMPKIKPLEVMCKPRPLMQPSMSK
RcF3′H2	MFLIVVITVLLAVILFRLLFSGKSRRHSLPLPPGPKPWPVVGNLPHLGPF<br>PHHSLADLAKKHGPLMHLRLGYVDVVVAASASVAAQFLKTHDANFSS<br>RPPNSGAKHLAYNYQDLVFAPYGPRWRMFRKISSVHLFSGKALDDLK<br>HVRQEEVAVLAHALANAGSKQVVNLAQQLNLCTVNALGRVMVGRR<br>VFGDGNGGDDRKADEFKSMVVEMMVLAGVFNIGDFIPSVEWLDLQG<br>VASKMKKLHKRFDDFLTAIVEDHKKKMSSGRAEQQVDMLTTLLSLKE<br>DADGEGAKLTDTEIKALLLNMFTAGTDTSSSTVEWALAELIRHPQMLE<br>QVQKELDQFVGRDRLVSESDLPNLAYLQAVIKETFRLHPSTPLSLPRMA<br>AESCEINGYHIPKGSTLLVNVWAISRDPAEWADPLEFRPERFLPGGEKP<br>NVDIRGNDFEVIPFGAGRRICAGMSLGLRMVHLMTATLVHAFDWGLP<br>DGLTPPKLNMDEAYGLTLQRAAPLMVHPRTRLASHAYKASSS
RcF3′H3	MLLPHFSSNDCVVSGFNIPQDTLLLVNAWAIHRDPKLWNEPESFKPERF<br>EGGGKDLAAHKLIPFGLGRRACPGVSLAQRVVGLTLASLIQSFEWERV<br>NEKEVDMTEGTGLTMPKLVPLEAMCKPRSFLNKILH
RcDFR1	MSSVDDLGPRLGPVNWYSSRDLKFRGTTHGEALGDHVMTRKLSLKQT<br>CSTSVLGYLLLEHGYTVRVTLRDPANMNKVKHLLGLPKAATHRTLWK<br>AELAVEGSFDEAIKGCTGVFHVTTPMDFESKDPENEVIKPAINGVLDIM<br>KACLKAKTVRRLVFTSSAGTVNVEEHQKPAYDESNWSDAKFCRKVK<br>MTGWEMKKKYPEYNVPTKFKGIEEKLTKVHFSSKKLLETGFEFKYSLE<br>DMFEGAVDACKAKGLLPPPTEKHDADDMQQRRPCDNILASIARGEGP<br>GDWGRYLAKNSLIVRTRLCDGRCWFCWIMARHETPRAWLHCPGHRA<br>RPWSVRQFYLLINSTAYYVSNMKKVKPLLDLPKAKTHLTLWKSDLDV<br>EGSFDEAIKGCAGVFHVATPMDFESKDPENEVIKPTINGMLDIMKACL<br>KAKTVSRLVFTSSAGTVTAEEHRKSVYDESNWSDIDFCRKVKMTGWM<br>YFVSKTLAEQAAWKFAKENKIDFITIIPTLVIGPFVSPSMPPSLITGLSPIT<br>GNEGHYSIIKQGQFVHLDDLCQAHIFLYEHPKAEGRYICSSHDATIHDV<br>AKLLREKYPEYNVPTKFKGIEDNLENIHFSSKKLIEAGFEFKYSLEDMF<br>VGAVDACKAKGLLPPNSAAHETN
RcDFR2	MGSESESVCVTGAAGFIGSWLVMRLLERNYTVRATNEVIKPTINGVLDI<br>MKACLKAKTVRRLVFTSSAGTVNVEEHQKLAYDESNWSDVEFCRKV<br>KMTGWMYFVSKTLAEQAAWKFAQENNIDFITIIPTLVVGPFLMPAMPP<br>SLITGLSPITGNEGHYSIIKQGQFIHLDDLCQSHIYLYEHPKAEGRYICSS<br>YDATIHDIAKLLREKYPEYNVPTKFKGIEENLTKVHFSSKKLLETGFEF<br>KYSLEDMFVGAVDSCKAKGLLPPPTEKHDADDSNVVHVKLTA
RcANS	MVTAASIGSRVESLASSGISKIPKEYVRPKEELINIGDIFEDEKSTVGPQV<br>PTIDLKEIDSEDIKVREKCREELKKAAVDWGVMHLVNHGISDELMDRV<br>RNAGQAFFDLPIEQKEKYANDQASGKIQGYGSKLANNASGQLEWEDY<br>FFHCVYPEDKLDLSIWPQTPTDYIVATSEYAKELRGLATKIMTILSLGLG<br>LEEGRLEKEVGGLEELLLQMKINYYPKCPQPELALGVEAHTDISALTFI<br>LHNMVPGLQLFYEGKWVTAKCVPNSIVMHIGDTLEILSNGKYKSILHR<br>GLVNREKVRISWAVFCEPPKEKIILKPLLETVSEEEPAIFPPRTFSEHIQH<br>KLFRKSQENLLSTKEAALISTDEAALMSTEEAAIISTNGADLISTKEAALI<br>STKDAA

RcUFGT1 MSRSHSIPLIDTAKLFSSHGAKCTIVTTPLNAPLFSKATQISGIELLLIKFP
STEAGLPPDCESADLITTPDMVEKFVKASILLESQVEQILDEHRPHCLVA
DAFFPWATDVAAKIGIPRLYFHGFGFFPLCASLSVMMNQPHPKLLSSDS
ESFIIPNLPDEIKMTRSRLPVFPILNGDQSEFIKMVKASQESEEKSYGVIV
NSFYELESAYADHYRKVFGRRAWHIGPVSLCNKVAEDKAKRGSMERS
TAEKHECLKWLDSKKPRSVVYVCFGSMVSFADCQLLEIAIGLEASGQE
FIWVVKKVRKEIEEWLPEGFEKRVEGKGLIITDWAPQVLILEHEAIGAF
VTHCGWNSTLEGVSAGVPMITWPLFGEQFYNEKLVTEILRIGVPVGSE
QWVLFVELSKKKEASVKREAIEKAVTRIMVGDEAEEIRSRAIELGKMA
RRAVVEGGSSFLDLTALVKELNHLVDA

RcUFGT2 METKTDRQLHIFFLPYMAQGHTLPLIGIAKLFASRGVKSTIITTPVNEPIF
TKAIQTSQSFSFEIELVVIKFPTAEVGLPEGIESTKLQRTKEMREKFFKAI
TLLEQQVEQILEQHHPHCLVADSLFHWATDVAAKFGIPRLIFHGPGFFP
MCAAISVMRYQPHAKVSSDSEPFVIPGLPDEIKMTRNQLPAFLTLNGKT
ELIKLVKASREAEERSYGIIVNSFYELEPYYADHYRKVLGKKSWHIGPV
SLCNKAEKDKSEGGREGSIIEVNECLNWLNSKKSNSVVYICFGSLTNFS
GCQLVEIALGLEASQQQFIWVVKKEKNDKEEWLPEGFEHRIEGKGLIIR
GWAPQLLILQHEAVGAFVTHCGWNSILEGVSAGVPMITWPVSADQFS
NEKLVTQILGIGVAVGAQKSEDGSMKSEARVKREAIEKAVTELMVGD
EQEEMRSKVSALRGMARSAVEKDGSSFSDLTALIEELRSLRSSFS

RcUFGT3 METKTHQQLNIFFLPYMAQGHTLPLIDIAKLFASHGVKSTIITTPLNAPL
FFKPIQTSKSLGFEIELLIIKFPCIEVGLPEGIESANLTTSREMKEKHFKAT
TLLEPKVEQILDQYRPHCLVADSLFHWATDVAAKFGIPRLIFHGTGFFP
LCASMSVMLYQPQLKVSSDSESFVIPHLPDEIQTTRNQVPAYLNLDGKT
ELIKLVRASREAEERSYGIIVNSFYELEPDYADHYRNVFGRKAWHIGPV
SLCNKAEKDKSERGRENSVDEVHDCFNWLNSKEPNSVVYICFGSLTNF
SDCQLVDIALALEATQQQFIWVVKKEKNDKQEWLPEEFEQRMEGKGL
IIRGWAPQLLILEHEAVGAFMTHCGWNSILEGVAAGVPMITWPVSGEQ
FYNEKLVTELLGIGVAVGAQKWTTFEEESQKTEASVKMEAIEKAVNEI
MVGDEAEEMRNRVKALGEIARRAVEECGSSFSDLTSLIEELRSLRS

RcUFGT4 METKSDRQLHIFFLPYMAQGHTLPLIDIAKLFASRGVKSTIITTPVNAPIF
TKAIQQSQSFGFEIELVVIKLPSAQVGLPEGIESTTKLQKTKEMGKEFIK
AITLLEQQVEQILDQHHPHCLVADSLFHWATDVAAKFGIPRLIFHGPGF
FPLCAIMSVMRYQPHMKVSSDSESFVIPNLPHEIKMTKNELPSFLTQNG
ETDFTKLLKACEEVDERSYGIIINSFYELEPDYADHYRKVFSRKAWHIG
PVSLCNKAEKEKLERGIREGLVDEVHECLNWLNSKKPSSVVYICFGSM
NSFGDCELLEIALGLEASRQQFIWVVKREKNVKGEWLPEGFEQRVEGK
GLIIRGWAPQLLILQHEAVGAFLTHCGWNSILEGVSAGVPMITWPLFED
QFSNEKLVTQILGIGVAVGAQKSEDGRTKSEASVKREAIEKAVTKIMV
GVEQEEMRKKVFALGEIARSTVEEGGSSFTDLTALIEELSTFKSTSVME
EEENVVQLIYKTYKKEHWGFIYLADLCIMLEFTQLRGDDEHVD

RcUFGT5 METKSHQKLHIFFLPFMGRGHTLPLIDIAKLFASRGVKSTMITTPANAPL
FSKAIQTSKSSGLEIELLLIKFPSTEVGLPEGIESTNWAETKEMREKFYK
ALTLLEPQVEQLLDQHRPHCLVASTLFHWTTDVAAKFGIPRLMFHGPG
YFPLCAAMSVRQYQPHMKVSSDSESFVIPNLPHEIKTTRNELPSFVTQN
GETELTKLLKACRETEERSYGTIINSFYELEPDYADHYRKVFGRKSWHI
GPVSLCNMAEKEKLERGREGSVVDQVHDCLNWLNSKKPNSVVYICFG
SINSFSDCELLEIALGLEASRQQFIWVVKREKNDNDEWLPEGFEQRMEG

RGLIIRGWAPQLLILQHEATGAFLTHCGWNSILEGVSAGVPMITWPVFA
DQFNNEKLVTQILGIGVAVGAQKSEDGSMKSEARVKREAIEKAVTEIM
VGDEQEEMRRKVFALAEMARRAVEEGGSSFTNLTALMEELRSFVS

RcUFGT6 MEKPAELVFIPSPGVGHLVSTVEIAKLLVSRDDQLLITVLIMKFPSDTLG
TDAYIESFTHTSNSKRINFINIPQVNVDNIKGNSGSFMIDFIESQQPNVRD
AVTKLAKSETRLAGFVIDMFCTSMIDVANEFGVPTYVFFTSGAATLGL
MFHLQALRDHDGQHMDWTEFKDSDAELLIPSFVNPLPAAKVLPGRLL
GKETANSFLDIIKRFRGTKGIVVNTFTELESHALHALLSDAEIPPVYPVG
PLLNLNSDNNSLDEAKQKSDILKWLDDQPPLSVVFLCFGSMGSFREDQ
VKEIAQALEHVGYRFLWSLRRPPPDGKIGFPSDYDDHRGVLPEGFLERT
ERTGKVIGWAPQNAILAHPSVGGFVSHCGWNSTLESLWHGVPVATWP
LYAEQQLNAFELVKELGLAVEIDMSYRNSNPVPVSAQIIERGIREVMEL
DSDIRRRVREMSVKSKKALMDGGSSYSSLGHFINQIYN

RcUFGT7 MEKPAELVFIPSPGIGHFVSTVEIAKLLVSRDDQLLITVLIMKFPSDTKV
TDAYIESFTHTSNLKRIKFINLPQVNMENTQGNSGSFMIDFVESQQPHV
KNAVTKLAESRTRLAAFVIDMFCVAMVDVANEFGVPTYVFFTSGAAT
LGVMFHLQALRDHDREYKDCIEFKDSDAKLLIPSFVNPLPAAKVLPGG
LFVKETANSLLNILQRFRGTKGILVNTFTELESHALRALLSDAEIPPVYP
PPLSVVFLCFGSMGSFGEDQVKEIAQALEHVGHRFLWSLRRPPPDGKIG
FPSDYDDDKEVLPEGFLDRTEGTGKVIGWAPQNAILAHPSVGGFVSHC
GWNSTLESLWHGVPVATWPLYAEQQLNAFELVKELGLAVEIDMSFRN
SSSSPIPVSAQKIESGIREVMELDSDIRNKVREMRVKSKKALMDGGSSY
SSLGHFINQIYN

RcUFGT8 MKKSAELVFIPSPGIGHLVSTVEIAKLLLSRTDQLLITVLIIKFPFSSDGTD
AYIESVAESSISQRIKFIDLPQKNMDTQDNGTTSFFKFIDAQQTNVKDVV
TKLIESESETRIAGFVIDMFCTSMIDVANEFGVPTYVFFTSGAGALGLMF
HMQQLRDDHNKHCIEFKDSTVELAIPSYANPLPAARVLPNVLFEKDVG
DGFLNFAKRFRDAKGILVNTFTELESYALHSLDSDGKIPPVYPVGPILNI
KNDDSDNQENSKQKSNILTWLDEQPPSSVVFLCFGSMGSFGEDQVKEI
ARALEHGGFRFLWSLRQPAPEGKFGFPSDYADHTRVLPEGFLDRTARI
GKVIGWAPQVTILAHPSVGGFVSHCGWNSTLESLWFGVPVATWPLYA
EQQLNAFELVTELNLAVEIDMSYRKDGPVLVSAQKIERGIKEVMELDS
DIRKKVIKMSENSKKALMEGGSSYSSLGHFIDQI

RcUFGT9 MKKATELIFIPIPGIGHIVSTVEIAKLLLSRDDNLFITILIMKFPFNVDGTD
AYIESLADPCSTLKTQRIKFVSLPQEQFQGNDATGFFTFIDSHKSHVKDA
VTKITESGPETRIAGLVIDMFCTGMIDVADEVGLPTYVFSTSGAANLGL
MFHLQALRDEQNKDCTEFKDSDAELVVPSFVNPLPAARVLPGVLFEKD
SMAGNLFLNFAKRFRETKGILVNTFLELELYALQSLSADGKVPPVYPIG
PILNVKSDDDKVGSEKSKQKSDILKWLDDQPPLSVVFLCFGSLGSFGED
QVKQIAYALEQGGFRFLWSLRQPPQEEAVFPSDYADYSRILPEGFLHRT
AVIGKVIGWAPQVDILAHPAVGGFVSHCGWNSTLESIWYGVPIATWPL
YAEQQVNAFELVKELKLAVEIDIGYRKDSGVVVSSQDIEKGITEVMEQ
DSELRKRVKEMSQMSKKALADDGSSYSSLGRFIDQI

RcUFGT10 MKKATELVFIPAPGIGHIVSAVEMAKLLVARDDQLFITVLIMKLPLDSK
PKGTDDTYSRSERIKFINLPEINIDTHGISPAYFFKLFVESHKPHIKDAVS
KLSQSQSNPLLAGFVIDMFCTRFLGLLFHLQTLSDEQNKDFTDFKDSDD
ELVLPSFVNPLPARALPSVFLDKEYTTIFVNIGRRFRETKGILVNTFMEL

	EAHALHSLSDGKTPPVYPVGPILNITGNENLEDYSDLAKQKTDSTLKW LDDQPPLSVVFLCFGSMGTFEEGQVKEIACALEQSGLRFLWSLREPPAK GKIAYPSDYAGYRGVLPEGFLDRTSGIGKVIGWAPQVAVLAHPAIGGF VSHCGWNSMLESLWYGVPVGTLPMYAEQHLNAFEMARELGLAVEVS MDYRKNSDFVNAEEIQRGIRQVMDHDSDTRKRVKKMSEMSKKALMD GGSSYSSLGRFIEQIFLTS
RcUFGT11	MIFLKVHRQMKKPAELVFIYLRCRCRSFLNSRRSRSPSSSYLETTTSSSP SHRKESILIIKDHQHVFLKVIENEETRRARVHPLRRCRPPHIDGRDRQAP RISRPYVESIEAASQKRIKFINLPEPNLDFTNMTRNEFRIAFMETHKPYV RDAVTELAESTESAPRLAGFVIDMFCTTMIDVANEFGVPTYVFFTSNAG FLGLMFHLQTIHDKHDMDVTELKSLETELVLPSFVNPVPHKALPSTLM DKEGATTFLDYARRFRETKGILVNTFSELESHALRSLSSYGEIPPVYPVG PVLKLKTEDDAHEGSDQATQKTDIIEWLDDQLPSSVVFLCFGSMGSFG EDQVKEIACALEQSGHRFLWSLRRPPPKGKITRPSDYTDPAGVLPDGFL ERTARIGKVIGWAPQVAVLAHPAVGGFVSHCGWNSTLESLWFGVPIAT WPMYAEQQMNAFEMVKELGLAVKISVEYDTAEENKMLLSAEEIERGI RELMEPGSDIRKRVKQMSEMSKTTLLDGGSSYSSLGRFIDQIFL
RcUFGT12	MKKATELVFIPAPGIGHIVSAVEMAKLLVARDDQLFITVLIMKLPLDSK PKGTDDTYSRSERIKFINLPGINIDTQGISPFNFFKLFVESHKPHIKDAVS KLSQSQSQSNPRLAGFVIDMFCTSMIDVANELGVPTYVFFASNAGFLGL VFHLQTLSDEHNKDFTDFKDSDDELVLPGFVNPLPARALPSVFLDKDN TATFVNIGRRFRETKGILVNTFMELEAHALHSLSDGKTPPVYPVGPILNI TTNENHEDYSDLAKQKADSTLKWLDDQPPLSVVFLCFGSMGTFDEDQ VKEIACALEQSGLRFLWSLREPPAEGKIAYPSDYTDYRGVLPEGFLDRT SGIGKVIGWAPQVAVLAHPAIGGFVSHCGWNSMLESLWYGVPVGTLP MYAEQHLNAFEMARELGLAVEVSMDYRKNSDFVNAEEIQRGIRQVM DHDSDTRKRVKEMSELSKKALMDGGSSYSSLGRFIEQIFLTS

Table S6 Correlation between RcBBX26 and anthocyanin biosynthetic genes.

	Correlation coefficient
RcPAL1	-0.4742
RcPAL2	-0.9505
RcC4H	-0.4139
Rc4CL1	-0.4176
Rc4CL2	-0.5314
Rc4CL3	-0.6679
Rc4CL4	0.5479
Rc4CL5	0.6036
Rc4CL6	0.9157
Rc4CL7	0.4187
Rc4CL8	0.5126
Rc4CL9	-0.3761
Rc4CL10	-0.3591
Rc4CL12	0.8950
RcCHS1	-0.5107
RcCHS2	-0.5095
RcCHI1	0.6627
RcCHI2	-0.4134
RcCHI3	-0.5394
RcFLS1	0.7244
RcFLS2	-0.4750
RcFLS3	0.1724
RcFLS4	0.1497
RcF3H1	0.9083
RcF3H2	0.1497
RcF3'H1	-0.0484
RcF3'H2	-0.5130
RcF3'H3	0.8366
RcDFR1	-0.5634
RcDFR2	-0.5070
RcANS	0.8449
RcUFGT1	-0.2783
RcUFGT2	-0.3360
RcUFGT3	-0.3190
RcUFGT4	-0.4387
RcUFGT5	-0.4761
RcUFGT6	-0.4060
RcUFGT7	-0.4188
RcUFGT8	0.6242
RcUFGT9	0.7021
RcUFGT10	-0.3505
RcUFGT11	0.8840
RcUFGT12	-0.4615

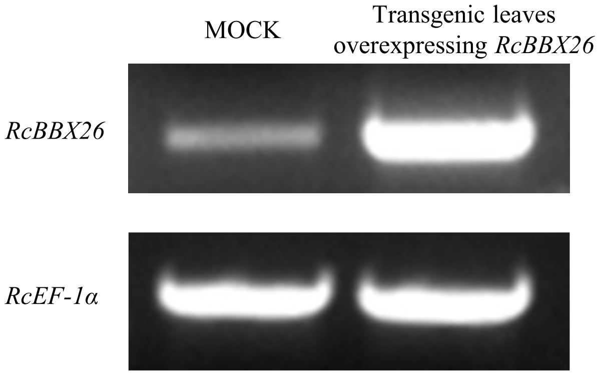
MOCK
Transgenic leaves overexpressing RcBBX26
RcBBX26
RcEF-1α

Figure S1

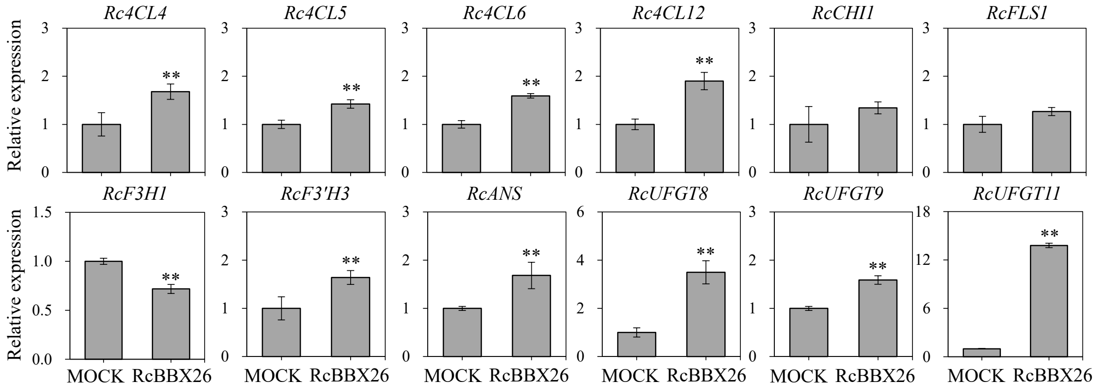
Rc4CL4
Rc4CL5
Rc4CL6
Rc4CL12
RcCHI1
RcFLS1
RcF3H1
RcF3'H3
RcANS
RcUFGT8
RcUFGT9
RcUFGT11
Relative expression
MOCK
RcBBX26
**

Figure S2
